# Classification and clustering on nocturnal polysomnography: distinctions and overlaps between central disorders of hypersomnolence

**DOI:** 10.1093/sleep/zsaf380

**Published:** 2025-12-04

**Authors:** Marta Karas, Yishu Gong, Marco Vilela, Emily Schlafly, Francesco Onorati, Alice Cai, Melissa Naylor, Derek L Buhl, Dmitri Volfson, Brian Tracey, Lucie Barateau, Yves Dauvilliers

**Affiliations:** Takeda Development Center Americas, Inc., Cambridge, MA; Takeda Development Center Americas, Inc., Cambridge, MA; Takeda Development Center Americas, Inc., Cambridge, MA; Takeda Development Center Americas, Inc., Cambridge, MA; Takeda Development Center Americas, Inc., Cambridge, MA; Takeda Development Center Americas, Inc., Cambridge, MA; Takeda Development Center Americas, Inc., Cambridge, MA; Takeda Development Center Americas, Inc., Cambridge, MA; Takeda Development Center Americas, Inc., Cambridge, MA; Takeda Development Center Americas, Inc., Cambridge, MA; Sleep-Wake Disorders Center, Department of Neurology, Gui de Chauliac Hospital, CHU, Montpellier, France; National Reference Network for Narcolepsy, Montpellier, France; Institute for Neurosciences of Montpellier (INM), INSERM, University of Montpellier, Montpellier, France; Sleep-Wake Disorders Center, Department of Neurology, Gui de Chauliac Hospital, CHU, Montpellier, France; National Reference Network for Narcolepsy, Montpellier, France; Institute for Neurosciences of Montpellier (INM), INSERM, University of Montpellier, Montpellier, France

**Keywords:** machine learning, nocturnal polysomnography, narcolepsy, idiopathic hypersomnia

## Abstract

**Study Objectives:**

Differential diagnosis of narcolepsy type 2 (NT2) from type 1 (NT1) and idiopathic hypersomnia (IH) is challenging due to overlapping symptoms. We developed an automated method using nocturnal polysomnography (nPSG) data to differentiate these conditions and clinical controls (CCs), and explored varying sleep phenotypes within NT1, NT2, IH, and CCs.

**Methods:**

We analyzed nPSG data from drug-free individuals with NT1, NT2, and IH, or CCs. Sleep features were derived at whole-night and per-quarter-night levels, including hypnogram, transition probability, hypnodensity, spindle, and quantitative electroencephalogram (qEEG) features. Random forest machine learning models were used for three classification tasks. Within-diagnosis clustering identified potential diagnosis subgroups.

**Results:**

The sample included 350 individuals (52% females; median age 30 years; 114 NT1, 90 NT2, 105 IH, and 41 CCs). Our models achieved area under the receiver operating characteristic curve values of 0.87, 0.79, and 0.82 for distinguishing NT2 from CCs, NT2 from IH, and IH from CCs, with corresponding *F*_1_ scores of 0.74, 0.71, and 0.69, respectively. qEEG features substantially contributed to model performance, distinguishing NT2 from IH. Cluster analysis revealed two NT1 subgroups (one showing more severe sleep disturbances), two NT2 subgroups (one trended toward NT1, the other toward IH), and two IH subgroups with differences in hypnodensity, qEEG, and spindle characteristics.

**Conclusions:**

Our exploratory findings demonstrate strong diagnosis classification performance from nPSG data alone, more easily distinguishing NT2 from CCs than from IH, and IH from CCs. The distinct NT2 subgroups suggest heterogeneity within NT2; further research is warranted to explore these patterns.

**Statement of Significance**

Accurate diagnoses of narcolepsy types 2 (NT2) and 1 and idiopathic hypersomnia (IH) remain challenging due to overlapping symptoms. We developed a machine learning model using drug-free nocturnal polysomnography data to automatically differentiate NT2 from clinical controls, NT2 from IH, and IH from clinical controls, with high accuracy. Our model leverages a rich set of sleep features, including spindle and quantitative electroencephalogram (qEEG) metrics. Furthermore, our analysis revealed distinct sleep phenotypes within each diagnosis, suggesting subtypes with varying levels of sleep disturbance and differences at qEEG and spindle levels. These findings provide a novel approach to classifying central disorders of hypersomnolence and suggest disease heterogeneity, which could lead to more accurate and timely diagnoses and personalized treatment strategies.

## Graphical Abstract

**Graphical Abstract:**
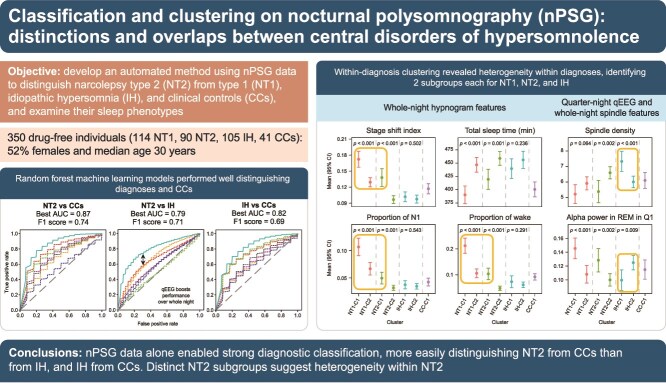

## Introduction

Narcolepsy type 1 (NT1), narcolepsy type 2 (NT2), and idiopathic hypersomnia (IH) are chronic, rare central disorders of hypersomnolence (CDHs), primarily characterized by excessive daytime sleepiness (EDS) [1]. Of these, NT1 is the most clearly defined due to the availability of a specific biomarker, low levels of cerebrospinal fluid (CSF) hypocretin-1/orexin-A (<110 pg/mL), and cataplexy. However, CSF sampling is invasive and not routinely performed, and cataplexy is a symptom (i.e. a sudden loss of muscle tone often triggered by emotion), unpredictable and rarely directly observed by the physician, that can be missed or misdiagnosed. Unlike NT1, the underlying causes of NT2 and IH are not well understood, and both NT2 and IH lack definitive biomarkers. NT2 shares similar neurophysiological diagnostic criteria with NT1 but has two key differences: people with NT2 do not have cataplexy and have normal levels of CSF orexin, if known [2]. Except for the long–sleep-duration phenotype, the only diagnostic feature differentiating NT2 and IH is the number of sleep-onset rapid eye movement (REM) periods during the multiple sleep latency test (MSLT). Diagnosing CDH is challenging due to overlapping clinical features, reliance on self-reporting for cataplexy, and limited reliability of sleep latency and the number of sleep-onset REM periods during the MSLT [3–6]. In recent years, discussions have arisen as to whether NT2 should be regarded as a distinct disease entity [1, 7, 8].

Characterizing sleep of NT1, NT2, and IH using nocturnal polysomnography (nPSG) is an active research area. Several sleep characteristics that distinguish individuals with narcolepsy from healthy controls have been identified, such as decreased REM sleep latency, sleep latency, and sleep efficiency, as well as increased wake after sleep onset duration and number of wake bouts [9–11]. Recent works have explored the potential of diagnosing NT1 through machine learning methods using nPSG-based whole-night sleep features [10, 12, 13], as well as features defined per quarter-night segments [13]. A recent study by Cesari et al. employed a similar approach and yielded similar results for NT1 to those of Vilela et al., also reporting some challenges in differentiating NT2 and IH [13, 14].

In addition, other studies have demonstrated the value of clustering techniques for understanding CDHs [15, 16]. These studies applied clustering to a combined dataset of all individuals and then characterized clusters using summary statistics, such as means and SDs, of sleep features. However, these clustering studies have some limitations, as it remains unclear whether individuals from the same diagnosis group but from different clusters have significantly different sleep features, or if individuals from different diagnosis groups in the same cluster are truly similar.

To address diagnostic challenges in CDHs, particularly in NT2, we aimed to understand how nPSG-derived sleep features vary within and between CDH diagnosis groups. Specifically, we aimed to address two objectives: (1) developing an accurate, automated machine learning method to distinguish individuals with NT2 from those with IH and clinical controls (CCs) (here, we built upon our previous work [Vilela et al. (2024)]), which has already demonstrated the feasibility of distinguishing NT1 from other CDH subtypes, including NT2, using similar machine learning approaches), and (2) understanding how sleep features differ within CDH diagnoses using clustering analysis.

## Methods

### Study design and population

The study population and nPSG data analyzed in this study were previously reported and included individuals with NT1, NT2, and IH and CCs with an EDS complaint but no CDH diagnosis (hypersomnolence not confirmed on tests) [13]. All the participants were referred to the Sleep Disorders Unit and French National Reference Center for Rare Hypersomnias in Montpellier from 2006 to 2020. All the participants were either drug-naïve or had discontinued any CNS drugs affecting sleep at least 3 weeks earlier. Regarding participant diagnoses, all the participants received clinical diagnoses made according to International Classification of Sleep Disorders, Third Edition, criteria based on standardized evaluations [17], which included a clinical interview by a sleep specialist and nPSG followed by the MSLT. The CSF orexin-A levels were measured in a subgroup using a radioimmunoassay kit. The IH subgroup included individuals with and without long sleep duration*,* defined as total sleep time above 19 hours on a controlled 32-hour bed rest polysomnography recording protocol [18]. Clinical controls were individuals with self-reported EDS who had a score greater than 10 on the Epworth Sleepiness Scale, who did not show abnormal MSLT features (i.e. sleep latency > 8 minutes or ≥2 sleep-onset REM periods [SOREMPs]) or evidence of cataplexy or excessive quantity of sleep, and who had adequate sleep efficiency (≥70%), low indices of sleep apnea (<15 events per hour), and low periodic leg movements (<15 events per hour). All the CCs tested had normal CSF orexin-A levels. None of the CCs had a SOREMP on the nPSG.

The study received approval from the institutional ethics committees (Comité de Protection des Personnes, France) under the protocol “Constitution of a cohort and of a clinical, neurophysiological, and biological bank of rare hypersomnolence disorders” (SOMNOBANK). Written informed consent was obtained from all the participants.

### Derivation of sleep features from nPSG data

Six sets of sleep features were derived from nPSG data: whole-night hypnogram features, whole-night spindle features, quarter-night hypnogram features, quarter-night stage transition probability features, quarter-night quantitative electroencephalogram (qEEG) features, and quarter-night hypnodensity features. These feature sets were described previously (see table 1 in Vilela et al. [13]) except for spindle features, added here because prior research indicates there may be differences in sleep spindles in different CDHs [10, 19–21] and because features of spindles are stable from one night to the next [22, 23], reviewed in Fernandez and Luthi [24], suggesting potential utility as a clinical biomarker.

The whole-night hypnogram features set consisted of standard sleep metrics measured across the entire nPSG period (defined from persistent sleep onset to final sleep epoch, following Vilela et al. [13], Morin et al. [25], and Smits et al. [26]) and included total sleep time, REM sleep onset, the stage shift index (SSI; number of transitions between sleep stages per hour), and proportion of time spent in each stage (N1, N2, N3, REM, and wake). Whole-night spindle features characterized sleep spindles (brief bursts of brain activity [0.5–3.0 s] during manually scored N2 sleep). Spindles were detected using the Luna toolbox for Python (https://zzz.bwh.harvard.edu/luna) [27]. The spindle features analyzed included the total count, density (number of spindles per minute of N2 sleep), average frequency (number of zero-crossings divided by spindle duration), average amplitude (the largest peak-to-peak amplitude), average duration, spindle–slow oscillation (SO) coupling proportion (proportion of spindles overlapping a detected SO), spindle–SO coupling angle (circular mean of SO phase at spindle peak), spindle–SO coupling phase locking (ranging from 0 to 1, with higher values indicating greater consistency in the spindle–SO coupling angles), spindle dispersion (measure of variability in spindle number across sleep epochs), and average spindle sigma-band isolation (sigma-band power enrichment relative to power enrichment in other limited bands). A more detailed description of the method and spindle features is included in Supplementary Material 1.

The quarter-night features captured time-resolved aspects of sleep by quarter night, with each participant’s night divided into four quarters from sleep onset to awakening (Q1–Q4). The quarter-night hypnogram features are similar to the whole-night set, but without REM sleep onset. Stage transition probability features describe the probability of transitioning between sleep stages. qEEG features were computed as described in Vilela et al. [13], using the C3 channel filtered to the range 0.5–47 Hz, and were analyzed using multitaper analysis into 2-s windows. EEG signals automatically screened for flat-line artifacts, saturation, and high-slew-rate events, and multitaper windows containing artifacts were discarded from subsequent analysis. Band power features were then extracted by measuring the normalized average band power for standard EEG bands—delta (0.5–4 Hz), theta (4–8 Hz), alpha (8–12 Hz), sigma (12–16 Hz), beta (16–30 Hz), and gamma (30–47 Hz)—in each quarter. Finally, the quarter-night hypnodensity features, derived from an established deep learning model trained to mimic human sleep stage scoring [12], represent the probability of each epoch belonging to a sleep stage or a mix of two stages; the entropy of these probabilities was also computed. Hypnodensity features capturing mixed states are particularly relevant to NT1, for which REM/wake mixture characteristics are considered to be a manifestation of the disease [28]. In total, we derived 330 sleep features across six feature sets.

### Machine learning framework for diagnosis classification

Our goal was to assess the predictive power of six sleep feature sets across three classification tasks: distinguishing (1) NT2 from CCs, (2) NT2 from IH, and (3) IH from CCs. We implemented a machine learning framework, following the methodology of Vilela et al. [13], which utilized a random forest (RF) classification model due to its strong predictive power and interpretability through feature importance metrics [29]. The framework’s outer loop involved repeatedly splitting the entire dataset via a Monte Carlo stratified split into a 70% training set and a 30% test set for *R* = 200 runs. Within each run, the 70% training set was used to find optimal RF hyperparameters through a 10-fold cross-validation process. For each fold of the cross-validation, the nine training portions were balanced using the synthetic minority oversampling technique (SMOTE) [30] before a model was trained and then validated on the remaining untouched portion. After the optimal hyperparameters were identified, a final RF model was trained on the *entire* 70% training set *without* using SMOTE. This final model was then used to generate performance metrics on the held-out 30% test set and to obtain feature rankings from the model trained on the 70% data. Feature importance was computed as the mean decrease in impurity, with interpretation based on relative ranking, as there is no standardized cutoff for importance values [31]. The performance metrics and feature rankings were then averaged across all 200 runs to yield the final results. This evaluation framework was applied separately for each of the three classification tasks and six sleep feature sets. A diagram of the machine learning framework is presented in Supplementary Figure S1.

Next, we assessed the benefits of combining eight “top features” across the six datasets into one model, including separate sensitivity analyses to evaluate the added contributions of qEEG and spindle feature sets. qEEG features were included in sensitivity analyses because they showed relatively high importance for distinguishing NT2 from IH in this study, while spindle features had not been explored in our earlier work [13] and were newly evaluated here. We also assessed the impact of preselecting eight “top features” during model training. Specifically, we compared the model performance under the following conditions: (1) using the eight “top features” from each feature set combined; (2) using all features from all feature sets combined; and (3) variations of condition (1) with specific features excluded—either excluding qEEG top features, spindle top features, or both. For these experiments, the machine learning evaluation framework used *R* = 500 outer-loop runs to ensure robust estimates of performance metrics such as area under the receiver operating characteristic (ROC) curve (AUC) and *F*_1_ score [32]. In the feature-exclusion experiments (condition 3), we also expanded the top feature list in a balanced manner across the retained sets as needed to maintain an equal number of features as in the model from condition (1) (i.e. 48 = 6 sets × 8 “top features”).

We present the results using ROC curves and report AUC and *F*_1_ score values aggregated (mean [SD]) across *R* runs. We also visualize the relative importance of the 10 top-ranked features from the best-performing model. Additionally, we quantify the difference in means between diagnosis class pairs (NT2 vs CCs, NT2 vs IH, IH vs CCs), expressed as a percentage relative to the second group in each pair, using a *t*-test for demographic and sleep features. In our report, original *t*-test *p*-values < .05 are marked with “^*^” in the table reports, and those that remained <.05 after Benjamini–Hochberg (BH) multiplicity correction for the false discovery rate (FDR) are additionally marked with “^”. The BH correction was applied across all tests across all pairwise comparisons simultaneously, as those comparisons involved overlapping subsets of participants. Given the exploratory nature of this study aimed at hypothesis generation rather than confirmatory testing, our results focus on findings based on uncorrected *p*-values. Throughout this work, *p*-values were evaluated against a significance level of .05. Additionally, demographic and sleep feature aggregates (mean [SD]) across diagnosis groups are reported.

### Clustering analysis framework

We conducted unsupervised participant clustering using nPSG data for each diagnosis group separately (further referred to as “within-diagnosis” clustering). The clustering utilized all 330 sleep features from the combined feature sets. The input preparation involved preprocessing steps that were conducted on the dataset that included all the participants. These steps first involved imputing missing data (up to 2%) with the mean, removing features with near-zero variance, and standardizing all features to have a mean of 0 and a variance of 1. Next, we performed a principal components analysis and defined a feature space as the smallest number of first principal components that accounted for at least 90% of the cumulative variance. Using this feature space, we conducted *k*-means clustering separately within each diagnosis group. The number of clusters was determined for each group by minimizing the Calinski–Harabasz index [33] over a range of values from 2 to 10. Next, we conducted *t*-tests to compare the means of 330 original sleep features, along with demographics and orexin-A levels, between the resulting clusters within each diagnostic group. We report original (uncorrected) *t*-test *p*-values; those < .05 are marked with “^*^” in the table reports, and those that remained < .05 after BH correction for the FDR are additionally marked with “^”. The BH correction was applied separately within each diagnosis group (NT1, NT2, and IH), as these comparisons involved nonoverlapping subsets of participants. For the supplemental analyses of across-diagnosis clustering, further method details are provided in Supplementary Material 1.

## Results

### Participant characteristics

The study population included 350 participants: 114 with NT1, 90 with NT2, 105 with IH (87 [82.9%] with a long sleep phenotype), and 41 CCs. Demographic and clinical characteristics are summarized across the four clinical diagnosis groups (NT1, NT2, IH, and CCs) and a combined sample (Table 1). Sex distribution varied, with the IH group having the highest percentage of females (76%) and the combined sample consisting of 52% females. Median age ranged from 26 to 35 years (median age 30 years) across the diagnosis groups. Body mass index (BMI) had comparable medians and ranges across diagnosis groups, with BMI data available for 74% of cases. The CSF orexin-A levels were measured in 208 (59%) participants using a radioimmunoassay kit, including 100 with NT1, 61 with NT2, 40 with IH, and 7 CCs. Among the participants who had a lumbar puncture to test their CSF orexin-A levels, 98 participants with NT1 had low CSF orexin-A levels (≤110 pg/mL) and two had intermediate levels (111–200 pg/mL); 10 participants with NT2 had intermediate levels and 51 had normal levels (>200 pg/mL); and four participants with IH had intermediate levels and 36 had normal levels. The NT1 group had a median orexin-A level of 19 pg/mL, compared with a range of 255–314 pg/mL for the other groups.

**Table 1:** Demographic and clinical characteristics across four diagnosis groups (NT1, NT2, IH, and clinical controls) and a combined sample

**Characteristic**	**NT1** ***n* = 114**	**NT2** ***n* = 90**	**IH** ***n* = 105**	**Clinical controls** ***n* = 41**	**Combined** ***N* = 350**
Sex, *n* (%)					
Female	45 (39)	39 (43)	80 (76)	19 (46)	183 (52)
Male	69 (61)	51 (57)	25 (24)	20 (49)	165 (47)
Age, years					
Mean (SD)	37 (15)	30 (11)	30 (11)	34 (12)	33 (13)
Median (min, max)	35 (18, 79)	26 (18, 63)	26 (18, 60)	31 (22, 67)	30 (18, 79)
BMI					
Mean (SD)	26 (5)	24 (5)	23 (4)	25 (7)	24 (5)
Median (min, max)	26 (17, 43)	23 (17, 41)	23 (17, 39)	22 (18, 40)	24 (17, 43)
Orexin-A, pg/mL					
Measurement available, *n* (%)	100 (88)	61 (68)	40 (38)	7 (17)	208 (59)
Mean (SD)	29 (27)	295 (90)	285 (89)	356 (102)	167 (150)
Median (min, max)	19 (0, 161)	284 (141, 508)	255 (125, 537)	314 (266, 552)	159 (0, 552)

Abbreviations: BMI, body mass index; IH, idiopathic hypersomnia; max, maximum; min, minimum; NT1, narcolepsy type 1; NT2, narcolepsy type 2.

### Diagnosis classifier results

**Figure 1:**
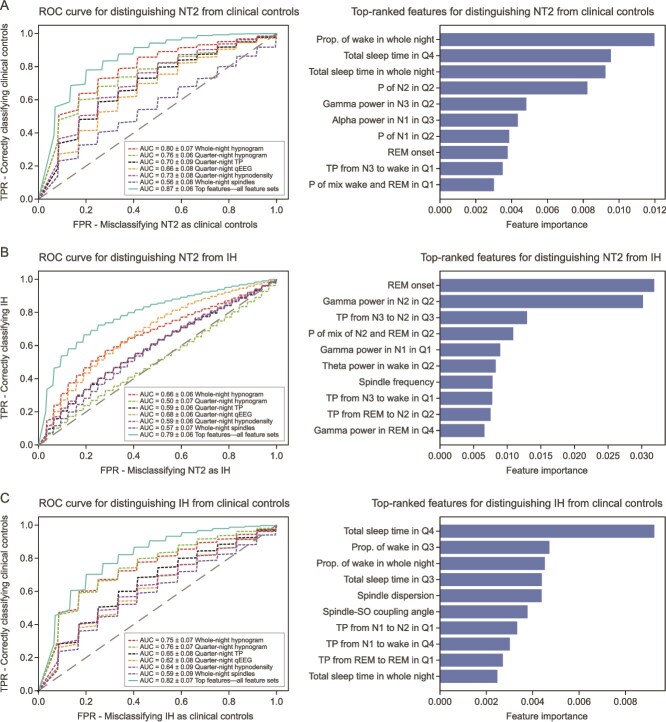
Model results distinguishing (A) narcolepsy type 2 (NT2) from clinical controls, (B) NT2 from idiopathic hypersomnia (IH), and (C) IH from clinical controls. The left-hand column plots show the performance of classification models for three tasks, with the lines representing receiver operating characteristic (ROC) curves showing the true positive rate (TPR; *y*-axis) versus the false positive rate (FPR; *x*-axis). Each ROC curve corresponds to a model trained on a different set of sleep features (solid lines for experiments with *R* = 500 runs of the machine learning framework outer loop, and dashed lines for *R* = 200). The legend at the bottom right of each plot shows the mean ± SD area under the ROC curve (AUC) across multiple experiment runs. The right-hand column plots show the 10 top-ranked features for the model with the highest AUC in a given classification task. Prop, proportion; P, probability; Q1/2/3/4, quarter 1/2/3/4 of the night; qEEG, quantitative electroencephalogram; REM, rapid eye movement; SO, slow oscillation; TP, transition probability.

We evaluated the predictive power of the six sleep feature sets across three classification tasks: distinguishing participants with (1) NT2 from CCs; (2) NT2 from IH; and (3) IH from CCs. ROC curves for models using different sleep feature sets showed the best performance was achieved when combining features from each feature set together (after feature selection), yielding AUC values of 0.87, 0.79, and 0.82, respectively, and *F*_1_ scores of 0.74, 0.71, and 0.69, respectively, for the three classification tasks (Figure 1 and Supplementary Table S1). Feature selection was important; across all three classification tasks, preselecting top features from individual feature sets consistently led to better performance than combining all feature sets into one large dataset for a single model. The latter approach resulted in AUC values up to 0.09 lower across the three classification tasks (Supplementary Figure S2). For reference, demographic and sleep feature aggregates (mean [SD]) across diagnosis groups are reported (Supplementary Table S2).

Our results suggest strong classification performance, with distinguishing participants with NT2 from CCs being easier than distinguishing participants with NT2 from those with IH and IH from CCs. Including quarter-night qEEG features substantially contributed to the best model performance in distinguishing NT2 from IH. Specifically, excluding quarter-night qEEG features reduced the AUC from 0.79 to 0.72 (Supplementary Figure S2 and Supplementary Table S1). In contrast, excluding the spindle features slightly reduced the AUC from 0.79 to 0.77 (Supplementary Figure S2 and Supplementary Table S1).

**Figure 2:**
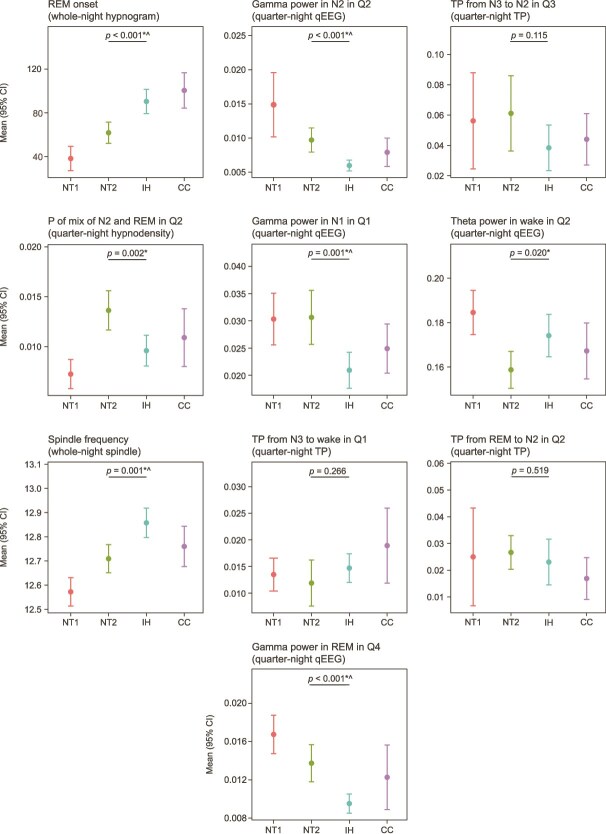
Means (95% CIs) for the 10 top-ranked features in the classification task distinguishing narcolepsy type 2 (NT2) from idiopathic hypersomnia (IH). Data for narcolepsy type 1 (NT1) and clinical controls (CCs) are shown. For each feature, the *p*-value from a *t*-test comparing the mean between NT2 and IH is displayed above the horizontal line indicating the comparison. ^*^p < .05; ^*p* < .05 after Benjamini–Hochberg correction for the false discovery rate. The plot titles indicate the feature name and the feature set from which they originate. P, probability; Q1/2/3/4, quarter 1/2/3/4 of the night; qEEG, quantitative electroencephalogram; REM, rapid eye movement; TP, transition probability.

The 10 top-ranked features from the best AUC model across the three classification tasks are listed (Figure 1, right plot column). Importance values are model-specific and thus should not be compared across the three plots, only within plots. Given the exploratory nature of this work, the pairwise between-diagnosis findings reported below are based on uncorrected *p*-values; postcorrection significance is provided in Supplementary Table S3, which includes *t*-test results for each pair of diagnoses across all sleep features and demographics (across all tests, of the 184 *p*-values < .05, 26 [14.1%] remained <.05 after BH multiplicity correction). For distinguishing NT2 from IH, the 10 highest-ranked features came from qEEG, transition probability, hypnodensity, spindle, and hypnogram feature sets, highlighting the value of deriving a rich set of sleep characteristics in the model classification performance. The distribution of the 10 top-ranked features (mean and 95% CI) across the diagnosis groups showed that compared with IH, the participants with NT2 had significantly lower REM sleep onset, higher gamma power in N1 in Q1, in N2 in Q2, and in REM sleep in Q4; lower theta power in wake in Q2, higher probability of mixed N2 and REM sleep in Q2, and lower spindle frequency (Figure 2). The remaining highly ranked features, though not individually significant in distinguishing NT2 from IH, may still aid classification. Because the RF model forms nonlinear combinations of input features, a feature can have high model importance even if it does not exhibit large between-group mean differences on its own. These features may contribute meaningfully at later splits in the decision tree, helping to refine classification in the context of interactions with other predictors.

For distinguishing NT2 from CCs, the 10 highest-ranked features came from hypnogram, hypnodensity, qEEG, and transition probability feature sets (Supplementary Figure S3). Among these, NT2 exhibited significantly higher total sleep time in Q4, total whole-night sleep time, and probability of mixed wake–REM sleep in Q1 compared with CCs. In contrast, NT2 showed significantly lower values for the proportion of wake in whole night, probability of N2 in Q2, alpha power in N1 in Q3, and REM onset.

For distinguishing IH from CCs, the 10 highest-ranked features came from hypnogram, spindle, and transition probability feature sets. The distribution of the 10 top-ranked features across the diagnosis groups showed that compared with CCs, participants with IH had significantly higher total sleep time in Q3, Q4, and whole night, and higher transition probability from REM sleep to REM sleep in Q1 (i.e. representing higher REM sleep stability) (Supplementary Figure S4).

### Participant clustering results

**Figure 3:**
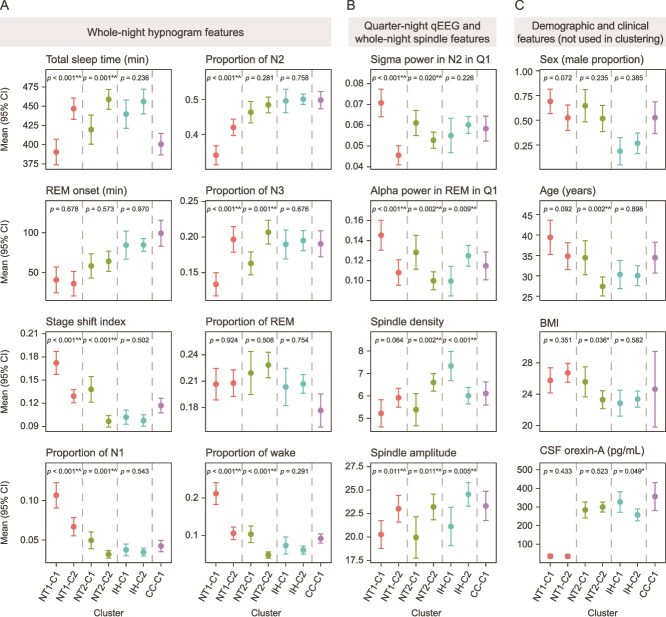
Characteristics of within-diagnosis clusters shown for selected sleep features. (A) Whole-night hypnogram features, (B) quarter-night quantitative electroencephalogram (qEEG) features and whole-night spindle features, and (C) demographic and clinical characteristics (not used in clustering but evaluated for the resulting clusters). Data were aggregated (mean [95% CI]) across seven out of nine identified clusters (clusters with <10 participants were excluded). Points indicate the clinical diagnosis within each cluster (narcolepsy type 1 [NT1], narcolepsy type 2 [NT2], idiopathic hypersomnia [IH], and clinical controls [CCs]), and dashed vertical lines separate clusters from the same diagnosis to aid comparison. *P-*values from a *t*-test comparing a feature value between clusters within diagnosis groups are shown. ^*^p < .05; ^*p* < .05 after Benjamini–Hochberg correction for the false discovery rate. BMI, body mass index; CSF, cerebrospinal fluid; Q1, quarter 1 of the night; REM, rapid eye movement.

In the within-diagnosis clustering analysis, we identified the following clusters: two in the NT1 group (denoted NT1-C1 and NT1-C2; sample sizes *n =* 55 and *n =* 59), two in the NT2 group (NT2-C1 and NT2-C2; *n =* 34 and *n =* 56), three in the IH group (IH-C1, IH-C2, and IH-C3; *n =* 32, *n =* 71, and *n =* 2), and two in the CC group (CC-C1 and CC-C2; *n =* 40 and *n =* 1). After excluding clusters with sample sizes of *n =* 1 and *n =* 2, two clusters remained in each of the NT1, NT2, and IH groups (NT1-C1 and NT1-C2, NT2-C1 and NT2-C2, and IH-C1 and IH-C2), and one cluster remained in the CC group (CC-C1). All the derived sleep features, demographics, and CSF orexin-A levels were compared between clusters within diagnosis groups using *t*-tests, indicating heterogeneity within the diagnosis groups (Supplementary Table S4). The selected sleep features across the clusters are shown in Figure 3. Although demographics and CSF orexin-A levels were not used in clustering, they were examined when comparing the resulting clusters. Given the exploratory nature of this work, the results below are based on uncorrected *p*-values; postcorrection significance is provided in Supplementary Table S4. Overall, of the 540 *p*-values < .05, 492 (91.1%) remained <.05 after BH multiplicity correction.

In the NT1 group, cluster NT1-C1 showed more pronounced disrupted nighttime sleep (DNS) characteristics compared with cluster NT1-C2, as indicated by whole-night hypnogram features. Specifically, cluster NT1-C1 had significantly lower total sleep time and proportion of time spent in N2 and N3, and higher SSI and proportion of time spent in N1 and in wake (Figure 3 and Supplementary Table S4). Additionally, cluster NT1-C1 had significantly higher power in the middle or higher EEG bands (alpha, beta, gamma, and sigma bands), and significantly lower power in the delta band across most sleep stages and quarters of the night, consistent with a higher level of overall cortical arousal. Among spindle features, cluster NT1-C1 had significantly lower spindle count, duration, amplitude, and dispersion, and significantly higher spindle–SO coupling angle and spindle–SO coupling phase locking. Few other significant differences in nPSG features were observed (Supplementary Table S4). There were no statistically significant differences between clusters NT1-C1 and NT1-C2 in age, BMI, sex, or CSF orexin-A levels (Figure 3 and Supplementary Table S4).

In NT2, cluster NT2-C1 exhibited more pronounced DNS characteristics compared with cluster NT2-C2, including significantly lower total sleep time and proportion of time spent in N3, along with higher SSI and a greater proportion of time spent in N1 and wake states (Figure 3 and Supplementary Table S4). Although less extreme than the NT1 clusters, these whole-night hypnogram features in cluster NT2-C1 showed a trend toward NT1 characteristics, whereas cluster NT2-C2 aligned more with IH clusters (Figure 3). At the qEEG level and similar to the pattern seen in the NT1 group, cluster NT2-C1, with higher DNS, also showed significantly greater power in the alpha, beta, gamma, and sigma bands and lower power in the delta band across most sleep stages and quarters of the night. Among spindle features, cluster NT2-C1 had significantly lower spindle count, density, amplitude, duration, and spindle–SO coupling phase locking. Few other significant differences in nPSG features were observed (Supplementary Table S4). Participants in cluster NT2-C1 also had a significantly higher mean age (Figure 3 and Supplementary Table S4). Orexin-A levels were comparable between the two NT2 clusters. There were also no significant differences in sex.

In IH, clusters IH-C1 and IH-C2 were quite similar in terms of whole-night hypnogram features (Figure 3 and Supplementary Table S4). However, significant differences were observed in the qEEG domain: cluster IH-C1 had significantly lower power in the alpha band, higher power in the delta band, and lower power in the theta band across most sleep stages and quarters of the night. Additionally, among spindle features, cluster IH-C1 had significantly lower spindle–SO coupling phase locking and higher density. Other significant differences in nPSG features were also observed, primarily in hypnodensity features. Cluster IH-C1 showed higher entropy across all night quarters and a higher probability of mixed sleep states (captured by pairwise probabilities) across most sleep stages and quarters of the night (Supplementary Table S4). Overall, these findings suggest potential heterogeneity within the IH group captured by hypnodensity, qEEG, and spindle features, which was not evident in traditional whole-night hypnogram features. There were no statistically significant differences between clusters IH-C1 and IH-C2 in age, BMI, or sex. Cluster IH-C1 had significantly higher mean CSF orexin-A levels (326.8 vs 264.8 pg/mL); however, mean values for both clusters were well within normal CSF orexin-A levels (Figure 3 and Supplementary Table S4).

To complement the within-diagnosis clustering approach, we also performed *k*-means clustering of all the participants combined (“across-diagnosis” clustering). Results are provided in Supplementary Figures S5–S7. This analysis yielded four clusters that did not clearly separate the four diagnoses; however, interpretation was limited, as some observed cluster characteristics were not consistent across diagnostic groups comprising a cluster and instead appeared to be driven by a single diagnosis group within a cluster. Accordingly, we did not place further emphasis on this analysis.

## Discussion

In our study, we developed an RF machine learning model using nPSG data to automatically distinguish NT2 from CCs, NT2 from IH, and IH from CCs, achieving AUC scores of 0.87, 0.79, and 0.82, respectively, and *F*_1_ scores of 0.74, 0.71, and 0.69, respectively. These results indicate strong classification performance, where distinguishing participants with NT2 from CCs was easier than distinguishing NT2 from IH and IH from CCs. Additionally, unsupervised clustering within diagnosis groups revealed two distinct clusters in NT1, which differed in DNS severity based on hypnogram features, as well as in qEEG, spindle, and other features. In NT2, two clusters emerged: one resembling NT1 and the second resembling IH based on hypnogram features, with further distinctions in qEEG, spindle, and additional features. No significant differences in orexin-A levels were observed between clusters identified in the NT1 or NT2 diagnoses. Similarly, two clusters were identified in IH, primarily differentiated by hypnodensity, qEEG, and spindle characteristics, but not by more commonly used hypnogram-based measures.

Our machine learning model performance analyses showed the benefit of using a rich set of nPSG features, including both whole-night and quarter-night sleep metrics, in the diagnosis classification tasks. Notably, we observed that the addition of qEEG features substantially contributed to model performance in distinguishing NT2 from IH (AUC values of 0.79 and 0.72 for the best model with and without qEEG, respectively). Analysis of the 10 top-ranked features showed that gamma power was higher in NT2 across multiple sleep stages and quarters of the night, with additional differences seen in theta power, and spindle frequency was higher in IH. Other features highly ranked for differentiating NT2 and IH included REM sleep onset, transition probabilities (from N3 to N2, from N3 to wake, and from REM to N3 across different quarters of the night), and hypnodensity features (probability of a mix of N2 and REM in Q2).

The AUC value of 0.79 and *F*_1_ score of 0.71 for separating NT2 versus IH data were in line with the *F*_1_ performance measures of 0.70 previously reported in a different dataset [14]. Although our work included qEEG features not examined by Cesari et al. [14], their work contained more detailed hypnodensity-based measures. The difficulty in separating NT2 from IH in both studies underscores challenges in separating these conditions based on nPSG data, which may reflect the presence of a complex, nonlinear feature structure as well as overlapping sleep phenotypes between these groups. This interpretation is supported by our clustering analysis. The across-diagnosis clustering, optimized for linear separability, did not clearly separate the four diagnoses, whereas the RF classifier, effective at capturing nonlinear decision boundaries and feature interactions, showed good discriminative performance, suggesting that the relevant diagnostic information likely resides in a complex, nonlinear structure of nPSG features. Additionally, the within-diagnosis clustering revealed two distinct subgroups in NT2: one showing sleep patterns similar to NT1 across all eight whole-night hypnogram features, reflecting a more severe DNS phenotype, and another displaying milder sleep disturbances and sleep characteristics more closely resembling IH, including comparable mean values for wake proportion and the SSI. This heterogeneity within NT2 may have contributed to the RF model's difficulty in clearly differentiating NT2 from IH.

Our results highlight several qEEG and spindle features that help to discriminate between CDHs, particularly IH and NT2, but more work is needed to validate these findings in additional datasets. As noted above, we found that gamma band power (30–47 Hz as described above) is higher in NT2 than in IH. Increased gamma band power can be interpreted as representing cortical arousal; for example, gamma power across both non-REM and REM sleep was found to be higher in participants with insomnia [34]. Thus, the lower gamma band power in IH may be consistent with lower neuronal excitability in this population [35]. However, higher-frequency EEG may be subject to contamination (including electromyogram or electrocardiogram) that is imperfectly removed by our artifact handling, so future work to confirm these gamma band findings is warranted.

Compared with qEEG features, spindle features were less valuable in discriminating between diagnoses. However, we also found higher spindle frequency in IH compared with NT2. Spindle frequency has not been well characterized across CDHs, especially in IH and NT2, so further investigation to validate these findings in additional datasets and to build upon work linking spindles to memory consolidation in these disorders is needed [36]. Although previous research indicates that spindle density is higher in individuals diagnosed with IH compared with individuals with narcolepsy [19, 20], our results did not identify spindle density as a top discriminating factor for IH.

In our analyses, some of the top features from the RF feature importance rankings (Figure 2) were not significant in univariate tests comparing the diagnoses, and, conversely, some significant features were not ranked at the top by the RF classification model. This discrepancy between RF feature importance and univariate significance is expected, as the two methods capture different aspects of the data [37]. The RF model can detect nonlinear relationships and interaction effects that an isolated univariate test cannot. Conversely, features significant in univariate tests may be downweighted in the model if their predictive information is redundant with other, more informative variables.

We acknowledge several strengths and limitations of our study. A key strength is the large sample of 350 drug-free individuals, all evaluated using the same procedures in a single lab and diagnosed according to International Classification of Sleep Disorders, Third Edition, criteria in a reference center by sleep experts. A large proportion of participants had CSF orexin-A measurements, which supported their diagnosis categories, especially for NT2. Additionally, our use of CCs, rather than healthy controls, makes our phenotyping tasks more representative of a typical sleep clinic scenario.

Methodologically, an important contribution is our approach of clustering within diagnosis groups, followed by formal statistical testing for differences between clusters. With the alternative across-diagnosis clustering approach, we found it challenging to draw conclusions about the similarities between diagnosis groups based solely on the characteristics of the formed clusters. This is because the cluster differences were often driven by participants from a single diagnosis class, rather than reflecting uniform differences across all diagnosis classes within the cluster. For classification results, we found that preselecting top features from individual feature sets and combining those together consistently led to better performance than using a large dataset combining all feature sets together, without the preselecting step. This is consistent with our previous results on distinguishing NT1 from NT2, IH, and CCs [13]. Indeed, feature preselection may help improve classifier performance by reducing redundancy, minimizing overfitting, and enhancing generalization to new data.

Regarding limitations, all the participants were diagnosed and had data collected at a single reference center, which may limit the generalizability of our findings. Verifying these findings with data from a larger, diverse, multisite dataset is an important next step. Additionally, the nPSG was performed in a sleep lab, where the “hospital effect” may differ from PSGs conducted at home in a more natural setting. The relatively small CC group may limit the statistical power of our analysis and the generalizability of our findings. Future studies with larger, more balanced cohorts are warranted to confirm these results.

To address class imbalance in the training set, we employed SMOTE (a technique previously applied in our work), which generates synthetic minority-class samples via interpolation. We selected SMOTE over a potential alternative, RF class weighting, based on prior evidence that resampling approaches can outperform class weighting when data are both imbalanced and complex [38, 39]. SMOTE can also expand the minority decision region and improve generalization, as shown by Chawla et al. [30], providing a more diverse representation during parameter optimization. Omitting SMOTE led to significantly biased classifiers [13]. SMOTE weighting is applied uniformly across all features each time a new synthetic sample is generated via interpolation (not extrapolation), which allows preservation of the physical and time-resolved structure, and the linear relationships intrinsic to the data. However, SMOTE has several limitations. Because it relies on linear interpolation, it cannot preserve nonlinear or physiological constraints and may produce implausible synthetic samples [30, 40]. It also assumes that Euclidean distance accurately reflects similarity, which may not hold in high-dimensional or structured data [41]. Additionally, SMOTE can lead to overfitting or increased class overlap, especially when applied to small datasets or when synthetic points are generated near decision boundaries [24, 30]. Synthetic data may also fail to capture true biological variability and cannot generate patterns beyond those present in the original dataset. Therefore, expanding the dataset with more real-world data would be preferable. To mitigate these limitations, we applied SMOTE only within the cross-validation folds during model tuning. The final model was then trained on the original, unaltered training data, with no synthetic samples included, thereby preserving the integrity of the reported performance and feature rankings.

A limitation of our spindle analysis is that the Luna detector has not been validated in CDHs. It is therefore unclear how these detections compare to manually annotated sleep spindles in CDH populations. For example, if spindles are altered in CDHs, different detection thresholds might be appropriate. Alternatively, signal contamination—for example, by microarousals [42, 43]—may be more common in CDHs. We selected Luna because it is a well-documented and freely available toolbox with a wavelet-based approach [44] that has been widely applied across healthy and clinical populations [45–50]. However, no studies have yet compared automated and human spindle detections in CDHs, highlighting an important direction for future validation.

Furthermore, while we sought to explore a wide range of features capturing multiple aspects of sleep, future research should explore additional sleep features. For example, muscle tone during REM has been shown to differ between NT1 and NT2 [51] and REM without atonia has been designated a biomarker of pediatric narcolepsy [52], suggesting features related to REM muscle tone and tonic/phasic REM substates could be of interest [53–56]. In addition, there are microstructural events besides sleep spindles shown to differ in CDHs, including slow waves [57–59], microarousals [42, 60, 61], or more broadly cyclic alternating pattern analysis, which encompasses a variety of phasic events against background activity [21, 62].

Our findings, along with those from our previous work [13], suggest that it is increasingly feasible to extract meaningful diagnostic information from nPSG alone. In this study, we intentionally prioritized interpretable features and models (e.g. RF) to support eventual clinical translation. While this may have constrained our ability to maximize predictive performance, we believe it enhances the practical relevance of our results. For example, sleep feature findings from Vilela et al. [13] were incorporated into our recent work on the effects of TAK-861 (oveporexton), an orexin receptor 2–selective agonist, on nocturnal sleep in individuals with NT1 [63]. Continued multicenter efforts, including those focused on optimizing predictive performance without regard to interpretability [12], will be essential to determine whether PSG-based approaches can reliably replace the MSLT in the diagnosis of CDHs.

While our results contribute to understanding central hypersomnolence disorder heterogeneity, the observed differences between diagnoses and within diagnoses should be considered exploratory and hypothesis-generating. Further confirmatory studies are warranted to validate these observations. Indeed, while the majority of within-diagnosis between-cluster comparisons remained statistically significant after BH correction for multiple comparisons (90%), only a small proportion remained statistically significant in the pairwise between-diagnosis comparisons (14%), underscoring the need for cautions interpretation.

In conclusion, our results suggest strong diagnosis classification performance based on nPSG data alone, with NT2 more easily distinguished from CCs than from IH, and IH from CCs. The difficulty in separating NT2 from IH, shown in our clustering results as two NT2 subgroups, with one resembling NT1 and the other closer to IH, suggests heterogeneity within the NT2 phenotype.

## Supplementary Material

Machine_Learning_NT1_NT2_IH_Supplementary_Material_25Nov2025_zsaf380

## Data Availability

The datasets and analytical code supporting this analysis will be shared on reasonable request to the corresponding author.
