# Supplementary material for: Classification and clustering on nocturnal polysomnography: distinctions and overlaps between central disorders of hypersomnolence
Source: Sleep. 2025 Dec 4;49(3):zsaf380. doi: 10.1093/sleep/zsaf380 (PMC13017669; doi:10.1093/sleep/zsaf380)
Supplement: Machine_Learning_NT1_NT2_IH_Supplementary_Material_25Nov2025_zsaf380 [file machine_learning_nt1_nt2_ih_supplementary_material_25nov2025_zsaf380.docx]

# Supplementary Material

# Classification and clustering on nocturnal polysomnography: distinctions and overlaps between central disorders of hypersomnolence

Marta Karas^1^, Yishu Gong^1^, Marco Vilela^1^, Emily Schlafly^1^, Francesco Onorati^1^, Alice Cai^1^, Melissa Naylor^1^, Derek L. Buhl^1,†^, Dmitri Volfson^1^, Brian Tracey^1^, Lucie Barateau^2–4^ and Yves Dauvilliers^2–4,*^

^1^Takeda Development Center Americas, Inc., Cambridge, MA, USA

^2^Sleep-Wake Disorders Center, Department of Neurology, Gui de Chauliac Hospital, CHU Montpellier, France

^3^National Reference Network for Narcolepsy, Montpellier, France

^4^Institute for Neurosciences of Montpellier (INM), INSERM, University of Montpellier, France

^†^At the time the study was conducted

*****Corresponding author. Yves Dauvilliers, National Reference Centre for Narcolepsy, CHU Gui de Chauliac, Montpellier, France; 80, avenue Augustin Fliche, 34295 Montpellier Cedex 5, France. Phone: +33467337478. Email: [ydauvilliers@yahoo.fr](mailto:ydauvilliers@yahoo.fr)

## Sleep spindle detection using Luna

Using the Luna package for Python (<https://zzz.bwh.harvard.edu/luna>; Purcell et al. [2017]) [1], we analyzed recordings from electroencephalogram (EEG) channel C3 during stage N2 sleep. Briefly, Luna transforms the EEG using a Morlet wavelet transform with center frequency 13.5 Hz. Putative spindles are identified by thresholding the wavelet coefficients (5 times the median). Candidates are then eliminated if the duration is not between 0.5 and 3.0 s; spindles occurring within 0.5 s of each other are merged unless the resulting spindle is longer than 3 s.

Prior to spindle detection, we used Luna's ARTIFACTS method to identify and exclude artifact epochs (30 s). We then used default parameters to Luna's SPINDLES method, except we set the detection threshold to 5 times the median. We used the median instead of the mean because the median is less sensitive to large fluctuations in amplitude as might occur with artifacts not caught by the artifact detector. In addition, we instructed Luna to detect slow oscillations (SO) and report coupling metrics using an amplitude threshold of 1.5 times the median and frequencies between 0.5–1.5 Hz, where parameters were selected based on prior work characterizing spindle-SO coupling [2-4].

To validate our thresholds, we built a custom dashboard in Python to visually inspect detections. We also compared statistics of our detections, including spindle density (6.04 /min ± 1.89), average amplitude (22.6 uV ± 5.87), average duration (1.07 s ± 0.17) and average frequency (13.3 Hz ± 0.39) to previous reports [1,5-8].

For each participant, we incorporated the following spindle characteristics into the feature set (the names in parentheses in all-capital letters refer to the parameter names in Luna):

- Count (*N*): total number of spindles detected (count)
- Density (*DENS*): number of spindles per minute of stage 2 sleep (count per minute)
- Average frequency (*FRQ*): intra-spindle frequency, computed by counting the number of zero-crossings and dividing by spindle duration (Hz), averaged over all detected spindles
- Average amplitude (*AMP*): the largest peak-to-peak amplitude in the bandpass filtered (11–15 Hz) signal (*μ*V), averaged over all detected spindles
- Average duration (*FWHM*): measured as the full-width-at-half-max (FWHM) of the peak in the wavelet transform (s), averaged over all detected spindles
- Coupling proportion (*COUPL_OVERLAP_PCT*): proportion of spindles coinciding with slow oscillations (SOs; ±3 s; unitless).
- Coupling angle (*COUPL_ANGLE*): for each spindle coinciding with an SO, Luna computes the mean phase of the SO across the spindle peaks $\theta_{i}$; for each patient, we report the angle $\theta=Arg(m)$ of the first circular moment $m=\frac{1}{N}\sum_{i} \frac{\theta_{i}}{\left| \theta_{i} \right|}$ (degrees)
- Phase-locking (*COUPL_MAG*): measured as intra-trial phase coherence, or the amplitude of the first circular moment $\left| m \right|$. Values range from 0 to 1, with higher values indicating greater consistency in the spindle-SO coupling angles (unitless).
- Dispersion (*DISPERSION*): variance in the number of spindles per epoch divided by mean number of spindles per epoch (unitless).
- Average sigma-band isolation (*Q*): sigma-band power enrichment relative to power enrichment in other limited bands (unitless), averaged over all detected spindles. Luna calculates the baseline power *P*^band^ in five fixed bands (delta 0.5–4 Hz; theta 4–8 Hz; slow-sigma 10–13.5 Hz; fast-sigma 13.5–16 Hz; beta 20–30 Hz). For the interval spanning the *i*-th spindle, the band power *P_i_*^band^ is calculated, and relative enrichment is computed on a log scale: $E_{i}^{\text{band}}=\log_{10} P_{i}^{\text{band}}-\log_{10} P^{\text{band}}$.

The Q-score is then computed as the difference between the maximum enrichment in the sigma bands and the maximum enrichment in the non-sigma bands:

$$Q_{i}=\max\left( E_{i}^{\text{slow-sigma}}, E_{i}^{\text{fast-sigma}} \right)-\max\left( E_{i}^{\text{delta}}, E_{i}^{\text{theta}}, E_{i}^{\text{beta}}, \right).$$

## Across-diagnosis clustering

To complement the within-diagnosis clustering approach, we utilized the feature space obtained from the principal components analysis in the within-clustering analysis to perform k-mean clustering of all participants combined (further referred to as "across-diagnosis" clustering). The number of clusters for each group was determined by minimizing the Calinski-Harabasz index over a range of values from 4 to 10, with the minimum value of 4 chosen to align with a previous report [9].

The analysis resulted in four clusters, denoted All-C1, All-C2, All-C3, and All-C4; cluster All-C4 was small, with only one participant (Supplementary Figure S4). Cluster All-C3 emerged as the second-largest cluster, predominantly consisting of participants with NT1 (64%). The two other large clusters, All-C1 and All-C2, did not have a dominant class, with NT1, NT2, and IH being more evenly distributed.

Both All-C1 and All-C2 clusters showed similar mean values for whole-night hypnogram features, indicated by substantial overlap in the 95% CIs of the mean, contrasting with All-C3 (Supplementary Figure S5). Specifically, clusters All-C1 and All-C2 had higher mean values for total sleep time, and the proportions of N2, N3, and REM sleep. Conversely, they had lower mean values for SSI and the proportion of N1 sleep and wake. However, when these features were examined in a further split by diagnosis groups (Supplementary Figure S6), limitations in interpretation arose. For instance, All-C3 had a lower proportion of N2 sleep compared with All-C1 and All-C2, indicated by nonoverlapping 95% CIs; however, this difference was mainly driven by participants with NT1 and was not observed in participants with NT2 (Supplementary Figures S5 and S6). Similar patterns can be seen for other sleep features. Therefore, it is challenging to draw conclusions about the similarities between diagnosis groups based solely on the characteristics of clusters formed in an across-diagnosis clustering approach; thus, we did not further pursue this analysis.

## Supplementary References

1. Purcell SM, Manoach DS, Demanuele C*, et al.* Characterizing sleep spindles in 11,630 individuals from the National Sleep Research Resource. *Nat Commun.* 2017;**8**:15930. doi:10.1038/ncomms15930

2. Winer JR, Mander BA, Helfrich RF*, et al.* Sleep as a potential biomarker of tau and β-amyloid burden in the human brain. *J Neurosci.* 2019;**39**(32):6315–6324. doi:10.1523/JNEUROSCI.0503-19.2019

3. Mylonas D, Machado S, Larson O*, et al.* Dyscoordination of non-rapid eye movement sleep oscillations in autism spectrum disorder. *Sleep.* 2022;**45**(3):zsac010. doi:10.1093/sleep/zsac010

4. Chen S, He M, Brown RE, Eden UT, Prerau MJ. Individualized temporal patterns dominate cortical upstate and sleep depth in driving human sleep spindle timing. *bioRxiv.* Published online February 27, 2024. doi:10.1101/2024.02.22.581592

5. Lacourse K, Yetton B, Mednick S, Warby SC. Massive online data annotation, crowdsourcing to generate high quality sleep spindle annotations from EEG data. *Sci Data.* 2020;**7**(1):190. doi:10.1038/s41597-020-0533-4

6. Christensen JAE, Kempfner L, Leonthin HL*, et al.* Novel method for evaluation of eye movements in patients with narcolepsy. *Sleep Med.* 2017;**33**:171-180. doi:10.1016/j.sleep.2016.10.016

7. Nicolas A, Petit D, Rompre S, Montplaisir J. Sleep spindle characteristics in healthy subjects of different age groups. *Clin Neurophysiol.* 2001;**112**(3):521-527. doi:10.1016/s1388-2457(00)00556-3

8. Dimitrov T, He M, Stickgold R, Prerau MJ. Sleep spindles comprise a subset of a broader class of electroencephalogram events. *Sleep.* 2021;**44**(9). doi:10.1093/sleep/zsab099

9. Šonka K, Šusta M, Billiard M. Narcolepsy with and without cataplexy, idiopathic hypersomnia with and without long sleep time: a cluster analysis. *Sleep Med.* 2015;**16**(2):225–231. doi:10.1016/j.sleep.2014.09.016

**
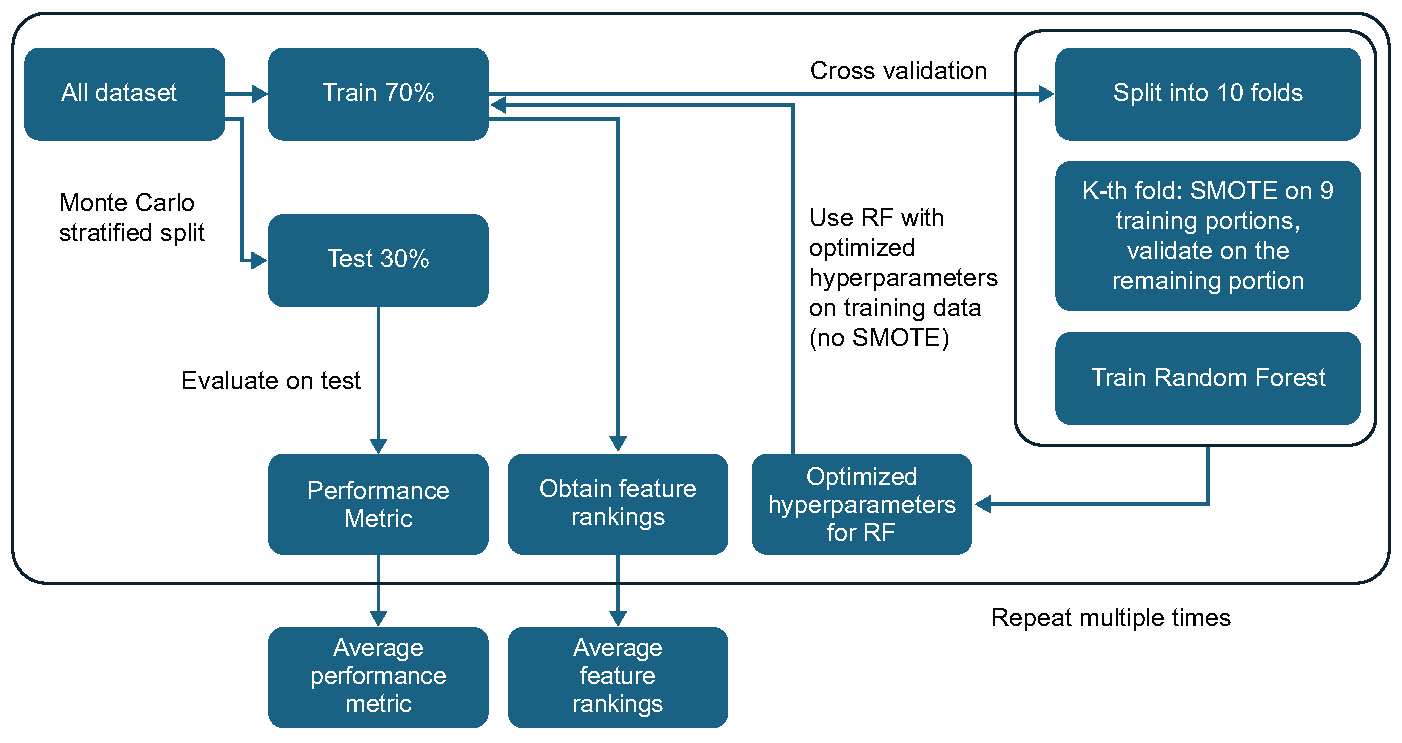
Supplementary Figure S1**. Diagram of the machine learning framework for diagnosis classification. RF, random forest; SMOTE, Synthetic Minority Oversampling Technique.


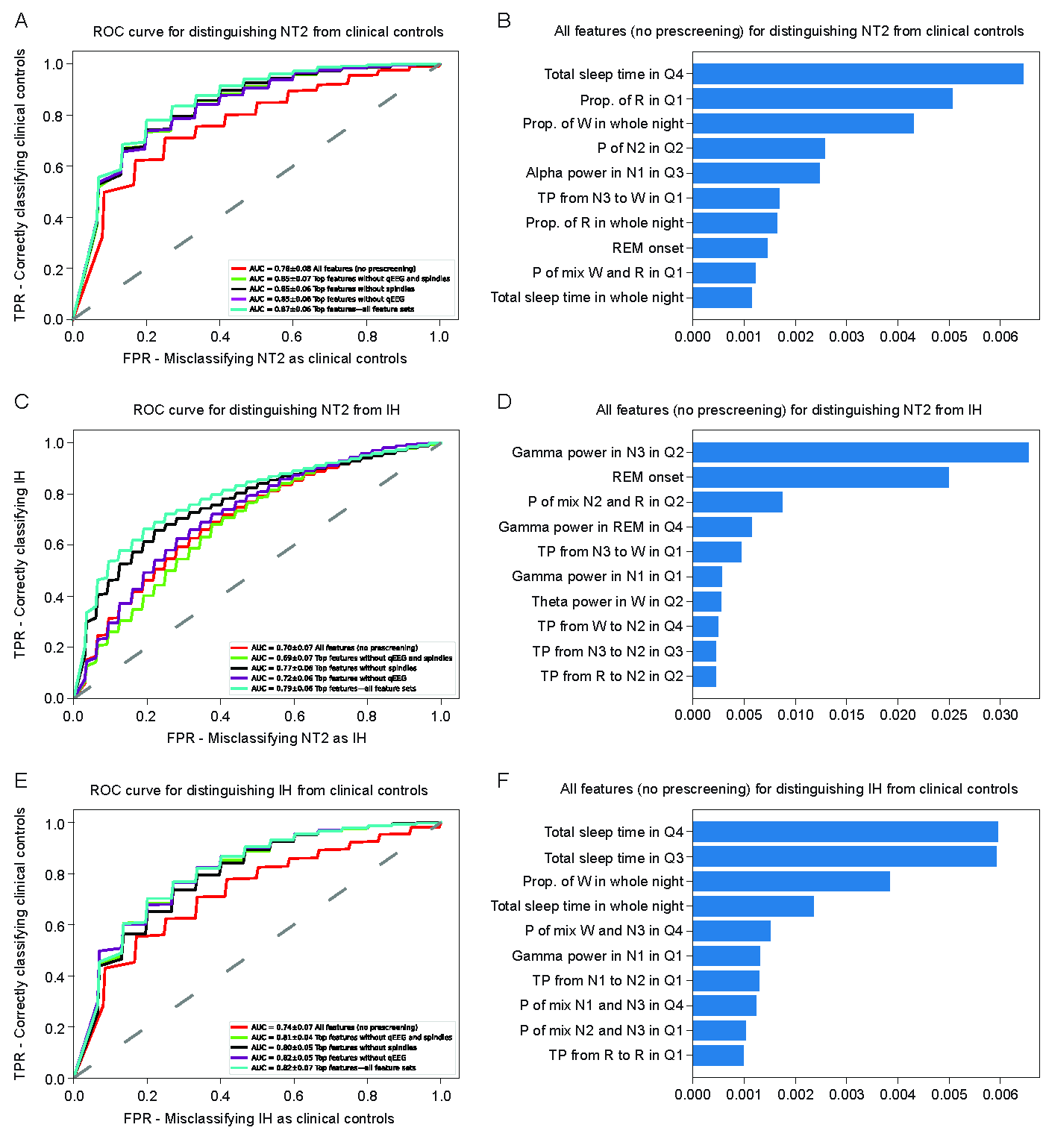


**Supplementary Figure S2**. Classification model results for the three tasks distinguishing (A) narcolepsy type 2 (NT2) versus clinical controls, (B) NT2 versus idiopathic hypersomnia (IH), and (C) IH versus clinical controls. The left column plots show the performance of classification models for three tasks, with the colored lines representing receiver operating characteristic (ROC) curves showing the true positive rate (TPR; y-axis) versus the false positive rate (FPR; x-axis). Models using different feature sets are compared: all features from all feature sets combined without features prescreening ("All features—no prescreening"), eight top features from each feature set combined ("Top features—all feature sets"), and variations of the latter—either excluding quantitative electroencephalogram (qEEG) top features, excluding spindle top features, or excluding both qEEG and spindle top features. The legend in the bottom-right of each plot shows the mean AUC value across multiple random forest repetitions (± SD). The right column plots show the 10 top-ranked features for the model using all features from all feature sets combined without features prescreening ("All features—no prescreening"). AUC, area under the receiver operating characteristic curve; FPR, false positive rate; TPR, true positive rate.

**
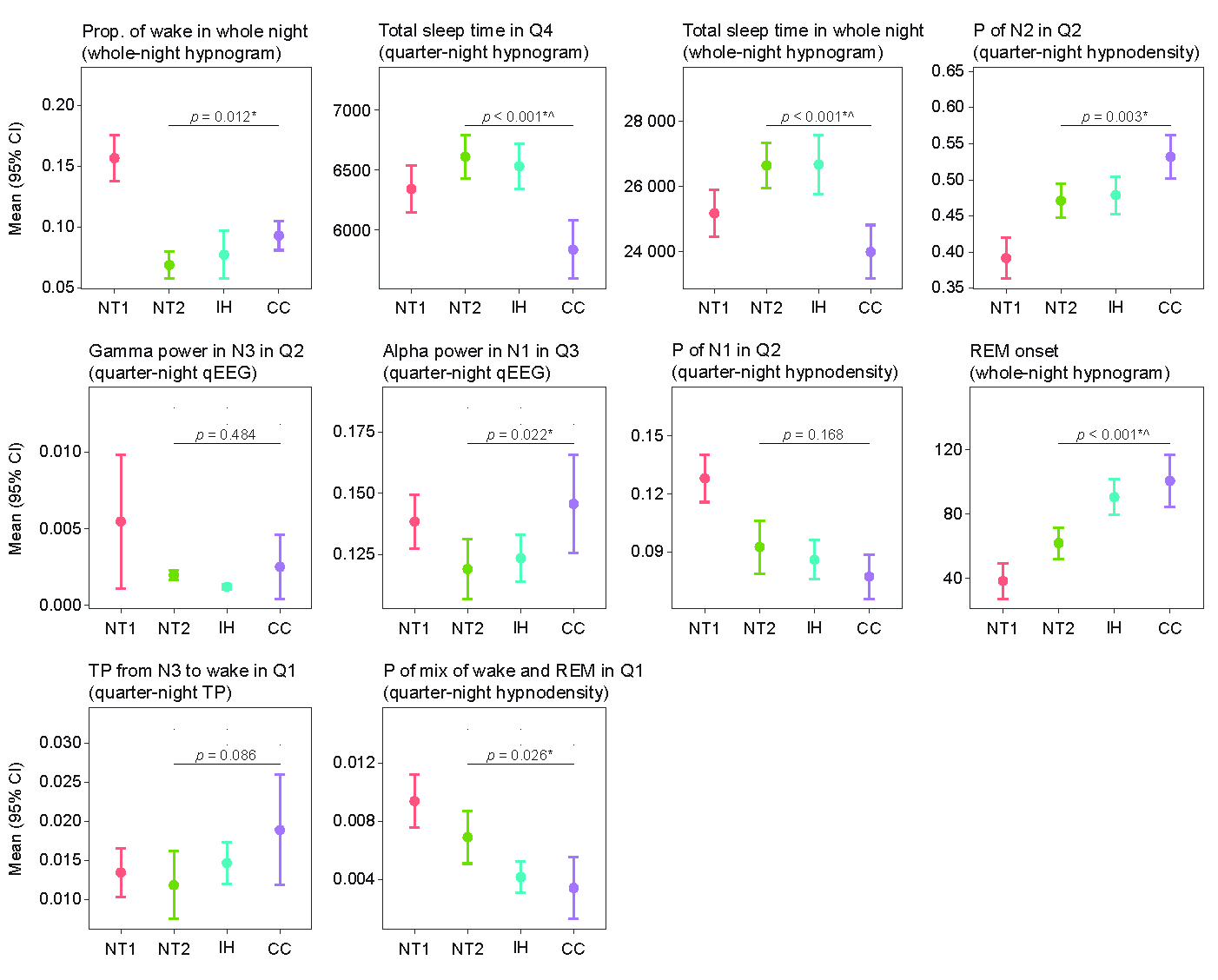
**

**Supplementary Figure S3**. Means (95% CIs) for the 10 top-ranked features in the classification task distinguishing narcolepsy type 2 (NT2) from clinical controls (CCs). For each feature, the *p*-value from a *t*-test comparing the mean between NT2 and CCs is displayed above the horizontal line indicating the comparison. **p*<0.05; ^*p*<0.05 after Benjamini-Hochberg correction for false discovery rate. The plot title indicates the feature name and the feature set from which it originates. NT1, narcolepsy type 1; P, probability; Prop., proportion; Q1/2/3/4, quarter 1/2/3/4 of the night; qEEG, quantitative electroencephalogram; REM, rapid eye movement; TP, transition probability.


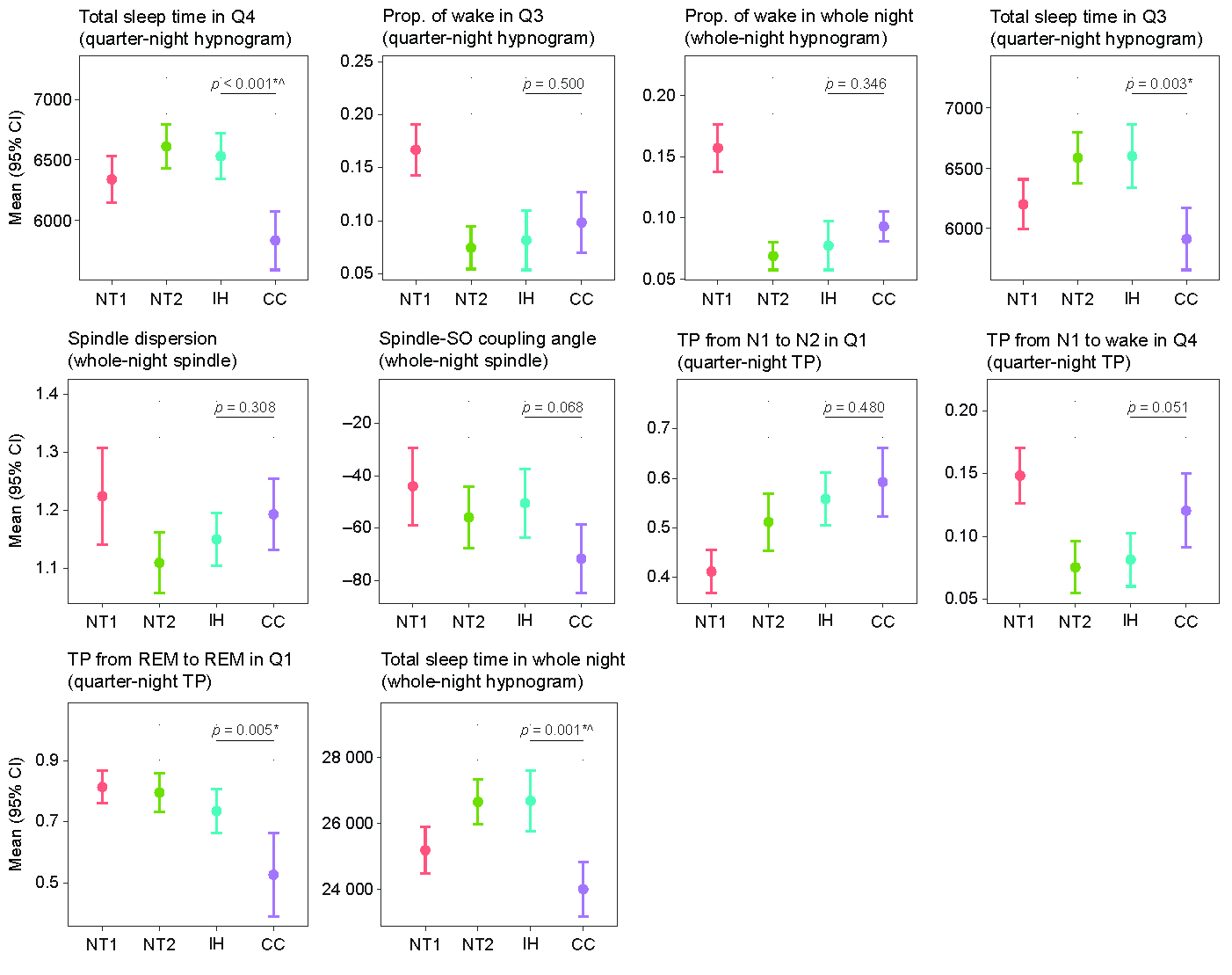


**Supplementary Figure S4**. Mean (95% CIs) for the 10 top-ranked features in the classification task distinguishing idiopathic hypersomnia (IH) from clinical controls (CCs). For each feature, the *p*-value from a *t*-test comparing the mean between IH and CCs is displayed above the horizontal line indicating the comparison. **p*<0.05; ^*p*<0.05 after Benjamini-Hochberg correction for false discovery rate. The plot title indicates the feature name and the feature set from which it originates. Prop., proportion; Q1/2/3/4, quarter 1/2/3/4 of the night; REM, rapid eye movement; SO, slow oscillation; TP, transition probability.

**
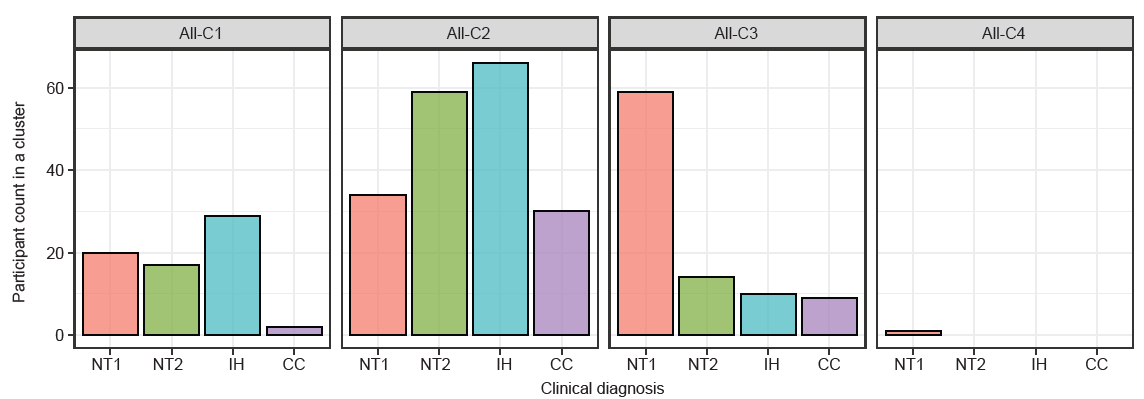
**

**Supplementary Figure S5**. Cluster composition for participants by diagnosis class cluster (narcolepsy type 1 [NT1], narcolepsy type 2 [NT2], idiopathic hypersomnia [IH], and clinical controls [CCs]) in across-diagnosis clustering.


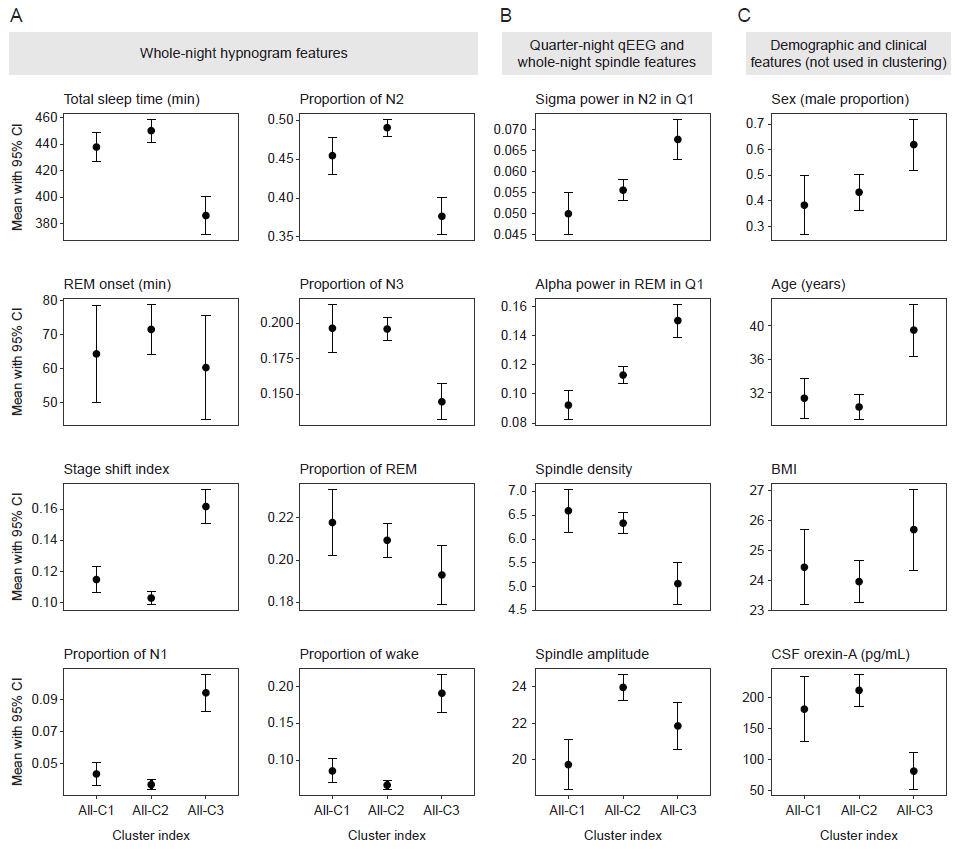


**Supplementary Figure S6**. Characteristics of across-diagnosis clusters shown for selected sleep features. (A) Whole-night hypnogram features, (B) quarter-night quantitative electroencephalogram (qEEG) features and whole-night spindle features, and (C) demographic and clinical characteristics (not used in clustering but evaluated for the resulting clusters). Data were aggregated (mean [95% CI]) across three out of four identified clusters (cluster with one participant only was excluded). BMI, body mass index; CSF, cerebrospinal fluid; Q1, quarter 1 of the night; REM, rapid eye movement.


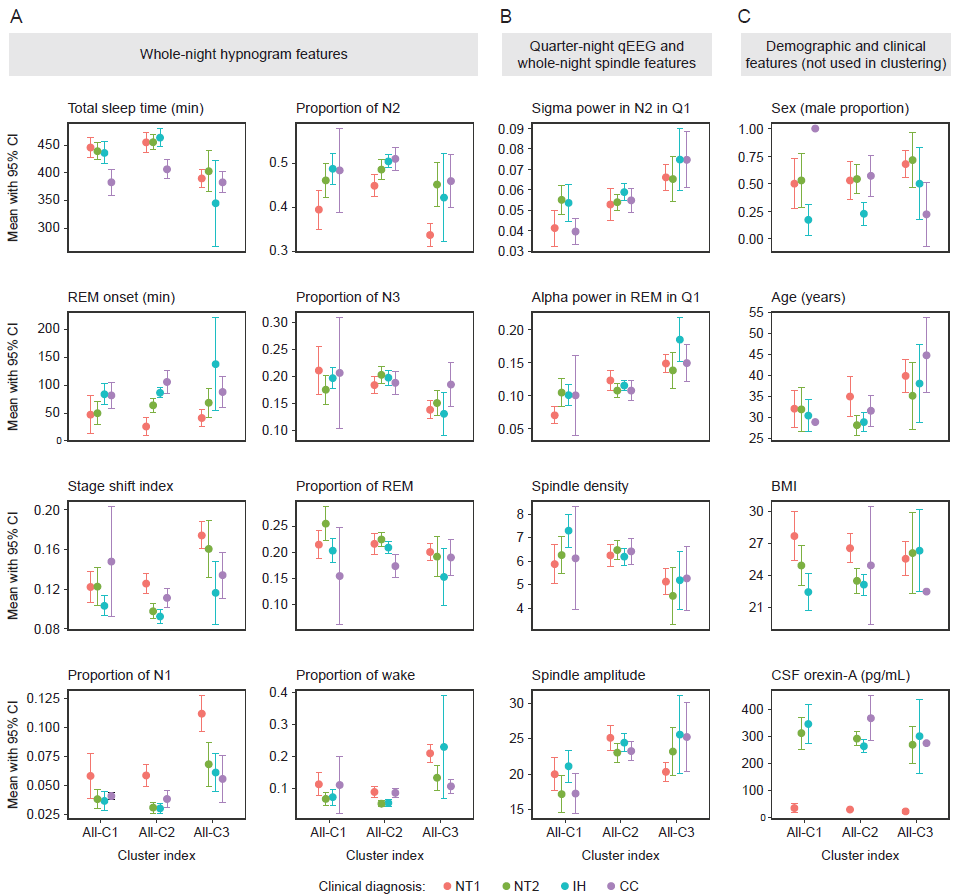


**Supplementary Figure S7**. Characteristics of across-diagnosis clusters shown for selected sleep features split by diagnosis group. (A) Whole-night hypnogram features, (B) quarter-night quantitative electroencephalogram (qEEG) features and whole-night spindle features, and (C) demographic and clinical characteristics (not used in clustering but evaluated for the resulting clusters). Data were aggregated (mean [95% CI]) across three out of four identified clusters (cluster with one individual only was excluded) and by diagnosis group (narcolepsy type 1 [NT1], narcolepsy type 2 [NT2], idiopathic hypersomnia [IH], and clinical controls [CCs]). BMI, body mass index; CSF, cerebrospinal fluid; Q1, quarter 1 of the night; REM, rapid eye movement.

**Supplementary Table S1.** Classification model performance statistics across models with different sleep feature sets used for three tasks: (1) distinguishing NT2 versus clinical controls, (2) NT2 versus IH, and (3) IH versus clinical controls

| **Sleep feature set(s) used in a model** | **Classification task** | **AUC, mean (SD)** | **F1 score, mean (SD)** | **R runs** |
| --- | --- | --- | --- | --- |
| Whole-night hypnogram | NT2 vs clinical controls | 0.80 (0.07) | 0.76 (0.06) | 200 |
| Whole-night hypnogram | NT2 vs IH | 0.66 (0.06) | 0.60 (0.06) | 200 |
| Whole-night hypnogram | IH vs clinical controls | 0.75 (0.07) | 0.72 (0.08) | 200 |
| Quarter-night hypnogram | NT2 vs clinical controls | 0.76 (0.06) | 0.74 (0.05) | 200 |
| Quarter-night hypnogram | NT2 vs IH | 0.50 (0.07) | 0.46 (0.07) | 200 |
| Quarter-night hypnogram | IH vs clinical controls | 0.76 (0.07) | 0.75 (0.06) | 200 |
| Quarter-night TP | NT2 vs clinical controls | 0.70 (0.09) | 0.72 (0.07) | 200 |
| Quarter-night TP | NT2 vs IH | 0.59 (0.06) | 0.53 (0.06) | 200 |
| Quarter-night TP | IH vs clinical controls | 0.65 (0.08) | 0.63 (0.08) | 200 |
| Quarter-night qEEG | NT2 vs clinical controls | 0.66 (0.08) | 0.72 (0.07) | 200 |
| Quarter-night qEEG | NT2 vs IH | 0.68 (0.06) | 0.61 (0.06) | 200 |
| Quarter-night qEEG | IH vs clinical controls | 0.62 (0.08) | 0.70 (0.06) | 200 |
| Quarter-night hypnodensity | NT2 vs clinical controls | 0.73 (0.08) | 0.76 (0.06) | 200 |
| Quarter-night hypnodensity | NT2 vs IH | 0.59 (0.06) | 0.54 (0.06) | 200 |
| Quarter-night hypnodensity | IH vs clinical controls | 0.64 (0.09) | 0.70 (0.08) | 200 |
| Whole-night spindle | NT2 vs clinical controls | 0.56 (0.08) | 0.63 (0.07) | 200 |
| Whole-night spindle | NT2 vs IH | 0.57 (0.07) | 0.54 (0.07) | 200 |
| Whole-night spindle | IH vs clinical controls | 0.59 (0.09) | 0.68 (0.06) | 200 |
| Top features—all feature sets | NT2 vs clinical controls | 0.87 (0.06) | 0.74 (0.07) | 500 |
| Top features—all feature sets | NT2 vs IH | 0.79 (0.06) | 0.71 (0.05) | 500 |
| Top features—all feature sets | IH vs clinical controls | 0.82 (0.07) | 0.69 (0.07) | 500 |
| All features (no prescreening) | NT2 vs clinical controls | 0.78 (0.08) | 0.78 (0.06) | 500 |
| All features (no prescreening) | NT2 vs IH | 0.70 (0.07) | 0.62 (0.07) | 500 |
| All features (no prescreening) | IH vs clinical controls | 0.74 (0.07) | 0.73 (0.05) | 500 |
| Top features without qEEG and spindle | NT2 vs clinical controls | 0.85 (0.07) | 0.74 (0.07) | 500 |
| Top features without qEEG and spindle | NT2 vs IH | 0.69 (0.07) | 0.63 (0.07) | 500 |
| Top features without qEEG and spindle | IH vs clinical controls | 0.81 (0.04) | 0.69 (0.05) | 500 |
| Top features without spindle | NT2 vs clinical controls | 0.85 (0.06) | 0.74 (0.06) | 500 |
| Top features without spindle | NT2 vs IH | 0.77 (0.06) | 0.68 (0.05) | 500 |
| Top features without spindle | IH vs clinical controls | 0.80 (0.05) | 0.69 (0.06) | 500 |
| Top features without qEEG | NT2 vs clinical controls | 0.85 (0.06) | 0.72 (0.06) | 500 |
| Top features without qEEG | NT2 vs IH | 0.72 (0.06) | 0.65 (0.06) | 500 |
| Top features without qEEG | IH vs clinical controls | 0.82 (0.05) | 0.69 (0.07) | 500 |

AUC, area under the receiver operating characteristic curve; IH, idiopathic hypersomnia; NT2, narcolepsy type 2; qEEG, quantitative electroencephalogram; TP, transition probability.

The AUC and F1 score values were aggregated (mean [SD]) across R = 200 or R = 500 runs of the outer loop in the machine learning framework.

**Supplementary Table S2.** Demographic and sleep feature characteristics aggregated (mean [SD]) across diagnosis groups

| **Feature name** | **Mean (SD)** | | | |
| --- | --- | --- | --- | --- |
|  | **NT1** | **NT2** | **IH** | **Clinical controls** |
| Age (not used in diagnosis classification) | 37.0 (14.5) | 30.1 (10.8) | 30.3 (10.6) | 34.4 (12.0) |
| BMI (not used in diagnosis classification) | 26.3 (4.65) | 24.2 (4.71) | 23.2 (4.31) | 24.6 (6.96) |
| Sex (prop. male) (not used in diagnosis classification) | 0.605 (0.491) | 0.567 (0.498) | 0.238 (0.428) | 0.513 (0.506) |
| Orexin-A (pg/mL) (not used in diagnosis classification) | 28.5 (27.3) | 295 (90.5) | 285 (88.6) | 356 (102) |
| wnSM_REMonSet | 38.2 (60.3) | 61.8 (47.1) | 90.4 (57.9) | 101 (52.8) |
| wnSM_propN1_all | 0.086 (0.057) | 0.038 (0.026) | 0.035 (0.022) | 0.043 (0.023) |
| wnSM_propN2_all | 0.382 (0.105) | 0.477 (0.088) | 0.493 (0.089) | 0.499 (0.078) |
| wnSM_propN3_all | 0.166 (0.073) | 0.190 (0.062) | 0.192 (0.061) | 0.189 (0.059) |
| wnSM_propR_all | 0.207 (0.063) | 0.225 (0.062) | 0.202 (0.058) | 0.176 (0.060) |
| wnSM_propW_all | 0.157 (0.105) | 0.069 (0.054) | 0.077 (0.103) | 0.093 (0.039) |
| wnSM_ssi_all | 0.150 (0.051) | 0.112 (0.042) | 0.098 (0.031) | 0.118 (0.031) |
| wnSM_tst_all | 420 (64.8) | 444 (55.3) | 445 (79.3) | 400 (44.8) |
| qnSM_propN1_Q1 | 0.064 (0.059) | 0.040 (0.033) | 0.039 (0.036) | 0.040 (0.033) |
| qnSM_propN1_Q2 | 0.081 (0.071) | 0.031 (0.031) | 0.027 (0.026) | 0.035 (0.030) |
| qnSM_propN1_Q3 | 0.095 (0.071) | 0.036 (0.032) | 0.033 (0.029) | 0.042 (0.029) |
| qnSM_propN1_Q4 | 0.104 (0.077) | 0.047 (0.037) | 0.041 (0.031) | 0.054 (0.035) |
| qnSM_propN2_Q1 | 0.363 (0.140) | 0.411 (0.118) | 0.425 (0.131) | 0.458 (0.125) |
| qnSM_propN2_Q2 | 0.413 (0.156) | 0.476 (0.136) | 0.495 (0.145) | 0.515 (0.124) |
| qnSM_propN2_Q3 | 0.415 (0.148) | 0.512 (0.140) | 0.542 (0.145) | 0.508 (0.157) |
| qnSM_propN2_Q4 | 0.339 (0.153) | 0.510 (0.127) | 0.511 (0.123) | 0.516 (0.154) |
| qnSM_propN3_Q1 | 0.288 (0.165) | 0.368 (0.143) | 0.370 (0.152) | 0.350 (0.139) |
| qnSM_propN3_Q2 | 0.153 (0.124) | 0.211 (0.143) | 0.219 (0.136) | 0.203 (0.134) |
| qnSM_propN3_Q3 | 0.114 (0.114) | 0.115 (0.100) | 0.103 (0.092) | 0.135 (0.117) |
| qnSM_propN3_Q4 | 0.110 (0.099) | 0.066 (0.083) | 0.075 (0.084) | 0.068 (0.079) |
| qnSM_propR_Q1 | 0.152 (0.103) | 0.122 (0.084) | 0.100 (0.084) | 0.077 (0.105) |
| qnSM_propR_Q2 | 0.173 (0.094) | 0.216 (0.102) | 0.187 (0.092) | 0.168 (0.079) |
| qnSM_propR_Q3 | 0.209 (0.110) | 0.262 (0.116) | 0.239 (0.106) | 0.217 (0.114) |
| qnSM_propR_Q4 | 0.295 (0.130) | 0.300 (0.108) | 0.282 (0.119) | 0.244 (0.126) |
| qnSM_propW_Q1 | 0.133 (0.141) | 0.058 (0.081) | 0.066 (0.123) | 0.076 (0.072) |
| qnSM_propW_Q2 | 0.179 (0.151) | 0.066 (0.072) | 0.071 (0.141) | 0.080 (0.061) |
| qnSM_propW_Q3 | 0.167 (0.130) | 0.075 (0.097) | 0.082 (0.146) | 0.099 (0.093) |
| qnSM_propW_Q4 | 0.149 (0.114) | 0.076 (0.070) | 0.090 (0.107) | 0.118 (0.091) |
| qnSM_ssi_Q1 | 0.130 (0.058) | 0.107 (0.051) | 0.097 (0.045) | 0.110 (0.053) |
| qnSM_ssi_Q2 | 0.153 (0.071) | 0.113 (0.050) | 0.097 (0.043) | 0.120 (0.039) |
| qnSM_ssi_Q3 | 0.150 (0.057) | 0.111 (0.053) | 0.094 (0.042) | 0.115 (0.053) |
| qnSM_ssi_Q4 | 0.165 (0.068) | 0.116 (0.054) | 0.101 (0.041) | 0.126 (0.041) |
| qnSM_tst_Q1 | 6456 (1176) | 6721 (914) | 6758 (1438) | 6113 (905) |
| qnSM_tst_Q2 | 6103 (1272) | 6637 (871) | 6701 (1517) | 6051 (785) |
| qnSM_tst_Q3 | 6201 (1125) | 6586 (1023) | 6599 (1374) | 5913 (836) |
| qnSM_tst_Q4 | 6340 (1054) | 6611 (880) | 6531 (987) | 5835 (794) |
| qnTM_N1toN1_Q1 | 0.381 (0.200) | 0.356 (0.259) | 0.368 (0.255) | 0.318 (0.208) |
| qnTM_N1toN1_Q2 | 0.395 (0.208) | 0.234 (0.234) | 0.235 (0.223) | 0.187 (0.201) |
| qnTM_N1toN1_Q3 | 0.447 (0.189) | 0.263 (0.236) | 0.265 (0.247) | 0.319 (0.216) |
| qnTM_N1toN1_Q4 | 0.437 (0.207) | 0.307 (0.238) | 0.314 (0.207) | 0.310 (0.180) |
| qnTM_N1toN2_Q1 | 0.412 (0.237) | 0.512 (0.279) | 0.558 (0.278) | 0.593 (0.226) |
| qnTM_N1toN2_Q2 | 0.368 (0.221) | 0.502 (0.356) | 0.499 (0.339) | 0.522 (0.315) |
| qnTM_N1toN2_Q3 | 0.308 (0.193) | 0.500 (0.301) | 0.528 (0.320) | 0.546 (0.245) |
| qnTM_N1toN2_Q4 | 0.284 (0.196) | 0.471 (0.290) | 0.513 (0.262) | 0.432 (0.211) |
| qnTM_N1toN3_Q1 | 0.004 (0.028) | 0.001 (0.011) | 0.000 (0.000) | 0.000 (0.000) |
| qnTM_N1toN3_Q2 | 0.000 (0.000) | 0.000 (0.000) | 0.003 (0.021) | 0.000 (0.000) |
| qnTM_N1toN3_Q3 | 0.000 (0.000) | 0.000 (0.000) | 0.000 (0.000) | 0.000 (0.000) |
| qnTM_N1toN3_Q4 | 0.000 (0.000) | 0.001 (0.008) | 0.000 (0.000) | 0.000 (0.000) |
| qnTM_N1toR_Q1 | 0.082 (0.130) | 0.055 (0.120) | 0.022 (0.069) | 0.015 (0.045) |
| qnTM_N1toR_Q2 | 0.069 (0.091) | 0.064 (0.168) | 0.043 (0.085) | 0.085 (0.154) |
| qnTM_N1toR_Q3 | 0.086 (0.095) | 0.123 (0.227) | 0.070 (0.124) | 0.067 (0.122) |
| qnTM_N1toR_Q4 | 0.112 (0.112) | 0.090 (0.121) | 0.091 (0.173) | 0.089 (0.124) |
| qnTM_N1toW_Q1 | 0.103 (0.123) | 0.054 (0.096) | 0.052 (0.092) | 0.050 (0.077) |
| qnTM_N1toW_Q2 | 0.141 (0.138) | 0.078 (0.150) | 0.087 (0.171) | 0.084 (0.131) |
| qnTM_N1toW_Q3 | 0.133 (0.095) | 0.070 (0.109) | 0.062 (0.110) | 0.068 (0.103) |
| qnTM_N1toW_Q4 | 0.148 (0.121) | 0.075 (0.100) | 0.081 (0.112) | 0.120 (0.096) |
| qnTM_N2toN1_Q1 | 0.028 (0.061) | 0.015 (0.020) | 0.010 (0.016) | 0.013 (0.018) |
| qnTM_N2toN1_Q2 | 0.027 (0.044) | 0.012 (0.018) | 0.006 (0.013) | 0.010 (0.014) |
| qnTM_N2toN1_Q3 | 0.030 (0.046) | 0.010 (0.015) | 0.008 (0.017) | 0.013 (0.015) |
| qnTM_N2toN1_Q4 | 0.034 (0.046) | 0.012 (0.018) | 0.009 (0.015) | 0.012 (0.016) |
| qnTM_N2toN2_Q1 | 0.869 (0.109) | 0.898 (0.053) | 0.912 (0.039) | 0.904 (0.043) |
| qnTM_N2toN2_Q2 | 0.887 (0.066) | 0.909 (0.050) | 0.911 (0.133) | 0.917 (0.027) |
| qnTM_N2toN2_Q3 | 0.891 (0.067) | 0.920 (0.055) | 0.926 (0.115) | 0.919 (0.044) |
| qnTM_N2toN2_Q4 | 0.873 (0.082) | 0.930 (0.037) | 0.937 (0.037) | 0.925 (0.036) |
| qnTM_N2toN3_Q1 | 0.039 (0.042) | 0.039 (0.036) | 0.035 (0.021) | 0.036 (0.019) |
| qnTM_N2toN3_Q2 | 0.020 (0.025) | 0.025 (0.032) | 0.019 (0.021) | 0.024 (0.021) |
| qnTM_N2toN3_Q3 | 0.014 (0.020) | 0.017 (0.018) | 0.011 (0.011) | 0.013 (0.013) |
| qnTM_N2toN3_Q4 | 0.016 (0.022) | 0.009 (0.013) | 0.007 (0.008) | 0.009 (0.012) |
| qnTM_N2toR_Q1 | 0.012 (0.016) | 0.019 (0.020) | 0.012 (0.010) | 0.010 (0.014) |
| qnTM_N2toR_Q2 | 0.009 (0.010) | 0.020 (0.022) | 0.014 (0.011) | 0.011 (0.010) |
| qnTM_N2toR_Q3 | 0.010 (0.013) | 0.019 (0.020) | 0.014 (0.016) | 0.016 (0.015) |
| qnTM_N2toR_Q4 | 0.022 (0.050) | 0.017 (0.013) | 0.015 (0.010) | 0.018 (0.018) |
| qnTM_N2toW_Q1 | 0.052 (0.059) | 0.029 (0.031) | 0.030 (0.032) | 0.037 (0.030) |
| qnTM_N2toW_Q2 | 0.058 (0.042) | 0.035 (0.025) | 0.031 (0.025) | 0.038 (0.024) |
| qnTM_N2toW_Q3 | 0.055 (0.037) | 0.034 (0.041) | 0.032 (0.047) | 0.039 (0.031) |
| qnTM_N2toW_Q4 | 0.054 (0.042) | 0.032 (0.021) | 0.032 (0.025) | 0.037 (0.026) |
| qnTM_N3toN1_Q1 | 0.003 (0.025) | 0.001 (0.004) | 0.002 (0.016) | 0.004 (0.015) |
| qnTM_N3toN1_Q2 | 0.000 (0.003) | 0.001 (0.006) | 0.001 (0.004) | 0.000 (0.000) |
| qnTM_N3toN1_Q3 | 0.001 (0.006) | 0.001 (0.004) | 0.001 (0.011) | 0.002 (0.008) |
| qnTM_N3toN1_Q4 | 0.003 (0.020) | 0.001 (0.006) | 0.000 (0.000) | 0.003 (0.018) |
| qnTM_N3toN2_Q1 | 0.040 (0.099) | 0.028 (0.038) | 0.025 (0.029) | 0.022 (0.023) |
| qnTM_N3toN2_Q2 | 0.031 (0.054) | 0.045 (0.077) | 0.037 (0.053) | 0.071 (0.177) |
| qnTM_N3toN2_Q3 | 0.056 (0.173) | 0.061 (0.120) | 0.038 (0.079) | 0.044 (0.055) |
| qnTM_N3toN2_Q4 | 0.049 (0.134) | 0.061 (0.141) | 0.042 (0.125) | 0.038 (0.062) |
| qnTM_N3toN3_Q1 | 0.908 (0.200) | 0.948 (0.109) | 0.939 (0.138) | 0.955 (0.028) |
| qnTM_N3toN3_Q2 | 0.755 (0.376) | 0.843 (0.279) | 0.897 (0.209) | 0.842 (0.272) |
| qnTM_N3toN3_Q3 | 0.690 (0.417) | 0.724 (0.381) | 0.709 (0.396) | 0.759 (0.359) |
| qnTM_N3toN3_Q4 | 0.684 (0.413) | 0.513 (0.450) | 0.558 (0.455) | 0.550 (0.450) |
| qnTM_N3toR_Q1 | 0.000 (0.003) | 0.000 (0.002) | 0.001 (0.004) | 0.000 (0.002) |
| qnTM_N3toR_Q2 | 0.000 (0.002) | 0.001 (0.005) | 0.001 (0.004) | 0.001 (0.003) |
| qnTM_N3toR_Q3 | 0.000 (0.001) | 0.000 (0.003) | 0.000 (0.003) | 0.001 (0.008) |
| qnTM_N3toR_Q4 | 0.000 (0.002) | 0.023 (0.148) | 0.001 (0.008) | 0.000 (0.000) |
| qnTM_N3toW_Q1 | 0.013 (0.017) | 0.012 (0.021) | 0.015 (0.014) | 0.019 (0.023) |
| qnTM_N3toW_Q2 | 0.020 (0.035) | 0.021 (0.033) | 0.018 (0.020) | 0.038 (0.065) |
| qnTM_N3toW_Q3 | 0.024 (0.070) | 0.013 (0.026) | 0.023 (0.038) | 0.023 (0.079) |
| qnTM_N3toW_Q4 | 0.028 (0.101) | 0.025 (0.109) | 0.018 (0.041) | 0.018 (0.057) |
| qnTM_RtoN1_Q1 | 0.022 (0.036) | 0.007 (0.018) | 0.010 (0.031) | 0.009 (0.021) |
| qnTM_RtoN1_Q2 | 0.022 (0.031) | 0.010 (0.023) | 0.009 (0.023) | 0.016 (0.031) |
| qnTM_RtoN1_Q3 | 0.033 (0.078) | 0.013 (0.024) | 0.010 (0.018) | 0.014 (0.025) |
| qnTM_RtoN1_Q4 | 0.034 (0.054) | 0.013 (0.020) | 0.010 (0.015) | 0.012 (0.022) |
| qnTM_RtoN2_Q1 | 0.019 (0.031) | 0.049 (0.080) | 0.029 (0.048) | 0.051 (0.125) |
| qnTM_RtoN2_Q2 | 0.025 (0.100) | 0.027 (0.031) | 0.023 (0.045) | 0.017 (0.026) |
| qnTM_RtoN2_Q3 | 0.012 (0.022) | 0.032 (0.107) | 0.016 (0.026) | 0.010 (0.015) |
| qnTM_RtoN2_Q4 | 0.013 (0.020) | 0.015 (0.024) | 0.012 (0.019) | 0.012 (0.022) |
| qnTM_RtoN3_Q1 | 0.000 (0.000) | 0.000 (0.000) | 0.001 (0.011) | 0.000 (0.000) |
| qnTM_RtoN3_Q2 | 0.000 (0.000) | 0.000 (0.000) | 0.000 (0.000) | 0.000 (0.000) |
| qnTM_RtoN3_Q3 | 0.001 (0.007) | 0.000 (0.000) | 0.000 (0.000) | 0.000 (0.000) |
| qnTM_RtoN3_Q4 | 0.000 (0.000) | 0.000 (0.001) | 0.000 (0.001) | 0.000 (0.000) |
| qnTM_RtoR_Q1 | 0.814 (0.287) | 0.795 (0.310) | 0.735 (0.376) | 0.526 (0.443) |
| qnTM_RtoR_Q2 | 0.846 (0.241) | 0.927 (0.054) | 0.908 (0.167) | 0.840 (0.283) |
| qnTM_RtoR_Q3 | 0.873 (0.191) | 0.924 (0.109) | 0.917 (0.162) | 0.916 (0.152) |
| qnTM_RtoR_Q4 | 0.894 (0.075) | 0.923 (0.108) | 0.927 (0.134) | 0.859 (0.248) |
| qnTM_RtoW_Q1 | 0.040 (0.051) | 0.026 (0.037) | 0.025 (0.051) | 0.024 (0.064) |
| qnTM_RtoW_Q2 | 0.045 (0.046) | 0.036 (0.043) | 0.031 (0.042) | 0.029 (0.030) |
| qnTM_RtoW_Q3 | 0.055 (0.065) | 0.031 (0.039) | 0.029 (0.024) | 0.035 (0.037) |
| qnTM_RtoW_Q4 | 0.059 (0.056) | 0.037 (0.033) | 0.032 (0.028) | 0.043 (0.028) |
| qnTM_WtoN1_Q1 | 0.223 (0.194) | 0.232 (0.218) | 0.236 (0.219) | 0.195 (0.164) |
| qnTM_WtoN1_Q2 | 0.242 (0.196) | 0.215 (0.179) | 0.278 (0.222) | 0.212 (0.171) |
| qnTM_WtoN1_Q3 | 0.246 (0.152) | 0.300 (0.228) | 0.311 (0.246) | 0.278 (0.227) |
| qnTM_WtoN1_Q4 | 0.272 (0.163) | 0.308 (0.191) | 0.339 (0.221) | 0.311 (0.238) |
| qnTM_WtoN2_Q1 | 0.178 (0.225) | 0.303 (0.314) | 0.335 (0.310) | 0.324 (0.321) |
| qnTM_WtoN2_Q2 | 0.124 (0.166) | 0.308 (0.246) | 0.302 (0.274) | 0.300 (0.276) |
| qnTM_WtoN2_Q3 | 0.086 (0.111) | 0.234 (0.251) | 0.236 (0.239) | 0.183 (0.200) |
| qnTM_WtoN2_Q4 | 0.073 (0.114) | 0.175 (0.175) | 0.145 (0.181) | 0.104 (0.157) |
| qnTM_WtoN3_Q1 | 0.023 (0.104) | 0.019 (0.118) | 0.024 (0.109) | 0.000 (0.000) |
| qnTM_WtoN3_Q2 | 0.003 (0.022) | 0.006 (0.034) | 0.018 (0.111) | 0.000 (0.000) |
| qnTM_WtoN3_Q3 | 0.009 (0.094) | 0.001 (0.013) | 0.000 (0.000) | 0.002 (0.013) |
| qnTM_WtoN3_Q4 | 0.000 (0.000) | 0.003 (0.026) | 0.000 (0.003) | 0.000 (0.000) |
| qnTM_WtoR_Q1 | 0.056 (0.126) | 0.056 (0.134) | 0.024 (0.070) | 0.008 (0.052) |
| qnTM_WtoR_Q2 | 0.047 (0.082) | 0.084 (0.161) | 0.056 (0.135) | 0.037 (0.087) |
| qnTM_WtoR_Q3 | 0.051 (0.080) | 0.105 (0.162) | 0.112 (0.151) | 0.062 (0.140) |
| qnTM_WtoR_Q4 | 0.101 (0.111) | 0.116 (0.159) | 0.101 (0.137) | 0.068 (0.118) |
| qnTM_WtoW_Q1 | 0.519 (0.287) | 0.346 (0.283) | 0.334 (0.288) | 0.473 (0.300) |
| qnTM_WtoW_Q2 | 0.583 (0.263) | 0.377 (0.258) | 0.327 (0.269) | 0.451 (0.254) |
| qnTM_WtoW_Q3 | 0.608 (0.221) | 0.360 (0.277) | 0.341 (0.286) | 0.475 (0.291) |
| qnTM_WtoW_Q4 | 0.554 (0.213) | 0.398 (0.263) | 0.415 (0.279) | 0.517 (0.283) |
| qnHYPN_N1N2_Q1 | 0.030 (0.017) | 0.029 (0.018) | 0.030 (0.016) | 0.027 (0.013) |
| qnHYPN_N1N2_Q2 | 0.036 (0.017) | 0.030 (0.021) | 0.028 (0.017) | 0.025 (0.013) |
| qnHYPN_N1N2_Q3 | 0.038 (0.018) | 0.031 (0.021) | 0.032 (0.018) | 0.027 (0.013) |
| qnHYPN_N1N2_Q4 | 0.040 (0.019) | 0.034 (0.022) | 0.037 (0.017) | 0.032 (0.019) |
| qnHYPN_N1N3_Q1 | 0.001 (0.001) | 0.001 (0.001) | 0.001 (0.001) | 0.001 (0.001) |
| qnHYPN_N1N3_Q2 | 0.001 (0.002) | 0.001 (0.001) | 0.001 (0.001) | 0.000 (0.000) |
| qnHYPN_N1N3_Q3 | 0.001 (0.001) | 0.000 (0.001) | 0.001 (0.001) | 0.000 (0.000) |
| qnHYPN_N1N3_Q4 | 0.000 (0.001) | 0.000 (0.000) | 0.000 (0.000) | 0.000 (0.000) |
| qnHYPN_N1R_Q1 | 0.013 (0.010) | 0.012 (0.009) | 0.009 (0.008) | 0.008 (0.009) |
| qnHYPN_N1R_Q2 | 0.013 (0.010) | 0.017 (0.011) | 0.014 (0.010) | 0.017 (0.011) |
| qnHYPN_N1R_Q3 | 0.016 (0.011) | 0.019 (0.013) | 0.019 (0.012) | 0.020 (0.014) |
| qnHYPN_N1R_Q4 | 0.024 (0.014) | 0.024 (0.015) | 0.022 (0.012) | 0.023 (0.016) |
| qnHYPN_N1_Q1 | 0.107 (0.058) | 0.089 (0.055) | 0.093 (0.054) | 0.081 (0.045) |
| qnHYPN_N1_Q2 | 0.128 (0.067) | 0.092 (0.066) | 0.086 (0.053) | 0.077 (0.037) |
| qnHYPN_N1_Q3 | 0.138 (0.063) | 0.099 (0.067) | 0.101 (0.057) | 0.086 (0.038) |
| qnHYPN_N1_Q4 | 0.171 (0.081) | 0.121 (0.078) | 0.123 (0.062) | 0.110 (0.056) |
| qnHYPN_N2N3_Q1 | 0.039 (0.027) | 0.050 (0.023) | 0.047 (0.024) | 0.054 (0.021) |
| qnHYPN_N2N3_Q2 | 0.028 (0.026) | 0.038 (0.025) | 0.042 (0.025) | 0.044 (0.026) |
| qnHYPN_N2N3_Q3 | 0.021 (0.022) | 0.024 (0.018) | 0.025 (0.020) | 0.026 (0.018) |
| qnHYPN_N2N3_Q4 | 0.018 (0.017) | 0.015 (0.016) | 0.018 (0.017) | 0.017 (0.016) |
| qnHYPN_N2R_Q1 | 0.006 (0.007) | 0.011 (0.011) | 0.007 (0.006) | 0.008 (0.008) |
| qnHYPN_N2R_Q2 | 0.007 (0.008) | 0.014 (0.010) | 0.010 (0.008) | 0.011 (0.009) |
| qnHYPN_N2R_Q3 | 0.008 (0.007) | 0.014 (0.012) | 0.010 (0.008) | 0.012 (0.009) |
| qnHYPN_N2R_Q4 | 0.010 (0.008) | 0.015 (0.013) | 0.012 (0.010) | 0.014 (0.010) |
| qnHYPN_N2_Q1 | 0.369 (0.143) | 0.414 (0.125) | 0.421 (0.136) | 0.470 (0.110) |
| qnHYPN_N2_Q2 | 0.392 (0.156) | 0.471 (0.112) | 0.479 (0.135) | 0.532 (0.099) |
| qnHYPN_N2_Q3 | 0.402 (0.145) | 0.485 (0.145) | 0.520 (0.147) | 0.526 (0.147) |
| qnHYPN_N2_Q4 | 0.333 (0.142) | 0.472 (0.134) | 0.489 (0.120) | 0.508 (0.133) |
| qnHYPN_N3R_Q1 | 0.000 (0.000) | 0.001 (0.003) | 0.000 (0.000) | 0.000 (0.001) |
| qnHYPN_N3R_Q2 | 0.000 (0.000) | 0.000 (0.001) | 0.000 (0.000) | 0.000 (0.000) |
| qnHYPN_N3R_Q3 | 0.000 (0.000) | 0.000 (0.001) | 0.000 (0.000) | 0.000 (0.000) |
| qnHYPN_N3R_Q4 | 0.000 (0.000) | 0.000 (0.000) | 0.000 (0.000) | 0.000 (0.000) |
| qnHYPN_N3_Q1 | 0.213 (0.175) | 0.276 (0.163) | 0.286 (0.168) | 0.275 (0.152) |
| qnHYPN_N3_Q2 | 0.114 (0.127) | 0.156 (0.123) | 0.171 (0.131) | 0.166 (0.118) |
| qnHYPN_N3_Q3 | 0.082 (0.097) | 0.075 (0.082) | 0.075 (0.082) | 0.095 (0.095) |
| qnHYPN_N3_Q4 | 0.069 (0.090) | 0.041 (0.059) | 0.047 (0.060) | 0.049 (0.063) |
| qnHYPN_R_Q1 | 0.102 (0.085) | 0.108 (0.097) | 0.073 (0.068) | 0.061 (0.076) |
| qnHYPN_R_Q2 | 0.112 (0.088) | 0.175 (0.112) | 0.136 (0.087) | 0.135 (0.065) |
| qnHYPN_R_Q3 | 0.137 (0.096) | 0.220 (0.113) | 0.177 (0.104) | 0.184 (0.100) |
| qnHYPN_R_Q4 | 0.196 (0.127) | 0.249 (0.126) | 0.206 (0.129) | 0.211 (0.107) |
| qnHYPN_WN1_Q1 | 0.026 (0.022) | 0.017 (0.016) | 0.018 (0.015) | 0.016 (0.013) |
| qnHYPN_WN1_Q2 | 0.032 (0.024) | 0.016 (0.017) | 0.017 (0.017) | 0.012 (0.008) |
| qnHYPN_WN1_Q3 | 0.032 (0.022) | 0.018 (0.019) | 0.018 (0.018) | 0.013 (0.008) |
| qnHYPN_WN1_Q4 | 0.037 (0.026) | 0.020 (0.019) | 0.021 (0.017) | 0.017 (0.009) |
| qnHYPN_WN2_Q1 | 0.019 (0.018) | 0.017 (0.019) | 0.019 (0.021) | 0.011 (0.007) |
| qnHYPN_WN2_Q2 | 0.022 (0.019) | 0.018 (0.021) | 0.023 (0.027) | 0.011 (0.006) |
| qnHYPN_WN2_Q3 | 0.020 (0.016) | 0.016 (0.019) | 0.020 (0.024) | 0.011 (0.009) |
| qnHYPN_WN2_Q4 | 0.017 (0.015) | 0.014 (0.015) | 0.017 (0.017) | 0.010 (0.005) |
| qnHYPN_WN3_Q1 | 0.010 (0.024) | 0.006 (0.013) | 0.012 (0.026) | 0.002 (0.005) |
| qnHYPN_WN3_Q2 | 0.007 (0.018) | 0.004 (0.007) | 0.008 (0.016) | 0.001 (0.002) |
| qnHYPN_WN3_Q3 | 0.004 (0.009) | 0.002 (0.004) | 0.003 (0.007) | 0.001 (0.002) |
| qnHYPN_WN3_Q4 | 0.003 (0.008) | 0.001 (0.004) | 0.002 (0.007) | 0.001 (0.001) |
| qnHYPN_WR_Q1 | 0.009 (0.010) | 0.007 (0.009) | 0.004 (0.006) | 0.003 (0.007) |
| qnHYPN_WR_Q2 | 0.011 (0.010) | 0.009 (0.008) | 0.007 (0.007) | 0.006 (0.006) |
| qnHYPN_WR_Q3 | 0.014 (0.010) | 0.013 (0.015) | 0.009 (0.009) | 0.007 (0.006) |
| qnHYPN_WR_Q4 | 0.018 (0.012) | 0.013 (0.012) | 0.011 (0.009) | 0.009 (0.007) |
| qnHYPN_W_Q1 | 0.210 (0.179) | 0.113 (0.116) | 0.127 (0.153) | 0.112 (0.106) |
| qnHYPN_W_Q2 | 0.254 (0.193) | 0.105 (0.109) | 0.128 (0.165) | 0.090 (0.074) |
| qnHYPN_W_Q3 | 0.241 (0.182) | 0.122 (0.133) | 0.127 (0.159) | 0.109 (0.106) |
| qnHYPN_W_Q4 | 0.230 (0.173) | 0.117 (0.105) | 0.135 (0.123) | 0.121 (0.088) |
| qnHYPN_entropy_Q1 | 0.551 (0.170) | 0.536 (0.178) | 0.531 (0.194) | 0.465 (0.129) |
| qnHYPN_entropy_Q2 | 0.571 (0.170) | 0.537 (0.190) | 0.545 (0.223) | 0.467 (0.116) |
| qnHYPN_entropy_Q3 | 0.561 (0.168) | 0.508 (0.196) | 0.508 (0.201) | 0.438 (0.130) |
| qnHYPN_entropy_Q4 | 0.612 (0.165) | 0.506 (0.187) | 0.520 (0.185) | 0.456 (0.146) |
| qnQEEG_mean_alpha_N1_Q1 | 0.142 (0.069) | 0.148 (0.070) | 0.150 (0.066) | 0.156 (0.047) |
| qnQEEG_mean_alpha_N1_Q2 | 0.137 (0.058) | 0.115 (0.055) | 0.125 (0.056) | 0.146 (0.070) |
| qnQEEG_mean_alpha_N1_Q3 | 0.138 (0.059) | 0.119 (0.058) | 0.123 (0.048) | 0.146 (0.065) |
| qnQEEG_mean_alpha_N1_Q4 | 0.141 (0.062) | 0.126 (0.055) | 0.136 (0.060) | 0.159 (0.063) |
| qnQEEG_mean_alpha_N2_Q1 | 0.097 (0.040) | 0.090 (0.029) | 0.093 (0.031) | 0.105 (0.033) |
| qnQEEG_mean_alpha_N2_Q2 | 0.103 (0.041) | 0.090 (0.033) | 0.090 (0.029) | 0.101 (0.035) |
| qnQEEG_mean_alpha_N2_Q3 | 0.105 (0.044) | 0.093 (0.034) | 0.096 (0.028) | 0.108 (0.033) |
| qnQEEG_mean_alpha_N2_Q4 | 0.107 (0.045) | 0.097 (0.034) | 0.102 (0.033) | 0.111 (0.037) |
| qnQEEG_mean_alpha_N3_Q1 | 0.048 (0.023) | 0.048 (0.023) | 0.045 (0.024) | 0.056 (0.026) |
| qnQEEG_mean_alpha_N3_Q2 | 0.055 (0.030) | 0.045 (0.022) | 0.044 (0.021) | 0.057 (0.027) |
| qnQEEG_mean_alpha_N3_Q3 | 0.054 (0.035) | 0.055 (0.024) | 0.056 (0.029) | 0.056 (0.027) |
| qnQEEG_mean_alpha_N3_Q4 | 0.050 (0.030) | 0.054 (0.028) | 0.059 (0.028) | 0.065 (0.028) |
| qnQEEG_mean_alpha_REM_Q1 | 0.126 (0.054) | 0.111 (0.041) | 0.117 (0.041) | 0.115 (0.035) |
| qnQEEG_mean_alpha_REM_Q2 | 0.128 (0.056) | 0.109 (0.040) | 0.121 (0.042) | 0.123 (0.035) |
| qnQEEG_mean_alpha_REM_Q3 | 0.124 (0.054) | 0.108 (0.036) | 0.117 (0.041) | 0.122 (0.033) |
| qnQEEG_mean_alpha_REM_Q4 | 0.126 (0.053) | 0.107 (0.033) | 0.117 (0.042) | 0.117 (0.037) |
| qnQEEG_mean_alpha_W_Q1 | 0.122 (0.066) | 0.095 (0.051) | 0.098 (0.055) | 0.111 (0.058) |
| qnQEEG_mean_alpha_W_Q2 | 0.137 (0.067) | 0.095 (0.049) | 0.099 (0.047) | 0.117 (0.052) |
| qnQEEG_mean_alpha_W_Q3 | 0.140 (0.070) | 0.108 (0.050) | 0.109 (0.050) | 0.144 (0.068) |
| qnQEEG_mean_alpha_W_Q4 | 0.138 (0.069) | 0.117 (0.053) | 0.128 (0.061) | 0.152 (0.074) |
| qnQEEG_mean_beta_N1_Q1 | 0.081 (0.043) | 0.093 (0.045) | 0.089 (0.042) | 0.095 (0.035) |
| qnQEEG_mean_beta_N1_Q2 | 0.075 (0.039) | 0.073 (0.045) | 0.068 (0.035) | 0.075 (0.033) |
| qnQEEG_mean_beta_N1_Q3 | 0.077 (0.039) | 0.072 (0.035) | 0.067 (0.034) | 0.084 (0.044) |
| qnQEEG_mean_beta_N1_Q4 | 0.076 (0.036) | 0.075 (0.032) | 0.082 (0.061) | 0.080 (0.028) |
| qnQEEG_mean_beta_N2_Q1 | 0.042 (0.023) | 0.043 (0.019) | 0.040 (0.020) | 0.043 (0.025) |
| qnQEEG_mean_beta_N2_Q2 | 0.045 (0.037) | 0.036 (0.016) | 0.030 (0.014) | 0.036 (0.019) |
| qnQEEG_mean_beta_N2_Q3 | 0.045 (0.031) | 0.037 (0.018) | 0.033 (0.013) | 0.038 (0.014) |
| qnQEEG_mean_beta_N2_Q4 | 0.049 (0.037) | 0.040 (0.016) | 0.041 (0.033) | 0.042 (0.018) |
| qnQEEG_mean_beta_N3_Q1 | 0.010 (0.009) | 0.010 (0.012) | 0.008 (0.004) | 0.016 (0.041) |
| qnQEEG_mean_beta_N3_Q2 | 0.015 (0.033) | 0.008 (0.004) | 0.007 (0.003) | 0.012 (0.022) |
| qnQEEG_mean_beta_N3_Q3 | 0.011 (0.008) | 0.011 (0.006) | 0.011 (0.012) | 0.012 (0.019) |
| qnQEEG_mean_beta_N3_Q4 | 0.014 (0.035) | 0.012 (0.007) | 0.012 (0.007) | 0.017 (0.035) |
| qnQEEG_mean_beta_REM_Q1 | 0.074 (0.038) | 0.062 (0.029) | 0.059 (0.028) | 0.062 (0.030) |
| qnQEEG_mean_beta_REM_Q2 | 0.067 (0.036) | 0.058 (0.027) | 0.055 (0.023) | 0.056 (0.021) |
| qnQEEG_mean_beta_REM_Q3 | 0.067 (0.035) | 0.060 (0.027) | 0.057 (0.023) | 0.056 (0.021) |
| qnQEEG_mean_beta_REM_Q4 | 0.066 (0.032) | 0.060 (0.024) | 0.056 (0.021) | 0.062 (0.025) |
| qnQEEG_mean_beta_W_Q1 | 0.066 (0.041) | 0.058 (0.043) | 0.052 (0.046) | 0.061 (0.046) |
| qnQEEG_mean_beta_W_Q2 | 0.073 (0.045) | 0.054 (0.034) | 0.048 (0.042) | 0.063 (0.037) |
| qnQEEG_mean_beta_W_Q3 | 0.078 (0.046) | 0.062 (0.037) | 0.061 (0.071) | 0.070 (0.034) |
| qnQEEG_mean_beta_W_Q4 | 0.076 (0.045) | 0.067 (0.037) | 0.071 (0.074) | 0.085 (0.087) |
| qnQEEG_mean_delta_N1_Q1 | 0.487 (0.127) | 0.481 (0.112) | 0.477 (0.112) | 0.464 (0.096) |
| qnQEEG_mean_delta_N1_Q2 | 0.501 (0.131) | 0.549 (0.109) | 0.544 (0.101) | 0.520 (0.130) |
| qnQEEG_mean_delta_N1_Q3 | 0.488 (0.119) | 0.535 (0.103) | 0.540 (0.096) | 0.488 (0.112) |
| qnQEEG_mean_delta_N1_Q4 | 0.485 (0.114) | 0.521 (0.093) | 0.505 (0.105) | 0.473 (0.102) |
| qnQEEG_mean_delta_N2_Q1 | 0.592 (0.096) | 0.608 (0.059) | 0.601 (0.067) | 0.589 (0.078) |
| qnQEEG_mean_delta_N2_Q2 | 0.578 (0.105) | 0.624 (0.072) | 0.627 (0.063) | 0.607 (0.079) |
| qnQEEG_mean_delta_N2_Q3 | 0.565 (0.099) | 0.606 (0.076) | 0.602 (0.058) | 0.578 (0.067) |
| qnQEEG_mean_delta_N2_Q4 | 0.552 (0.100) | 0.582 (0.066) | 0.570 (0.074) | 0.556 (0.071) |
| qnQEEG_mean_delta_N3_Q1 | 0.791 (0.068) | 0.796 (0.060) | 0.805 (0.056) | 0.767 (0.089) |
| qnQEEG_mean_delta_N3_Q2 | 0.763 (0.098) | 0.806 (0.061) | 0.809 (0.052) | 0.771 (0.077) |
| qnQEEG_mean_delta_N3_Q3 | 0.772 (0.096) | 0.766 (0.066) | 0.764 (0.076) | 0.767 (0.083) |
| qnQEEG_mean_delta_N3_Q4 | 0.763 (0.104) | 0.752 (0.086) | 0.737 (0.078) | 0.719 (0.098) |
| qnQEEG_mean_delta_REM_Q1 | 0.493 (0.093) | 0.534 (0.080) | 0.522 (0.079) | 0.526 (0.092) |
| qnQEEG_mean_delta_REM_Q2 | 0.515 (0.109) | 0.546 (0.083) | 0.530 (0.078) | 0.520 (0.077) |
| qnQEEG_mean_delta_REM_Q3 | 0.513 (0.097) | 0.542 (0.075) | 0.528 (0.082) | 0.519 (0.073) |
| qnQEEG_mean_delta_REM_Q4 | 0.511 (0.094) | 0.539 (0.065) | 0.524 (0.076) | 0.517 (0.072) |
| qnQEEG_mean_delta_W_Q1 | 0.556 (0.144) | 0.618 (0.129) | 0.628 (0.129) | 0.605 (0.149) |
| qnQEEG_mean_delta_W_Q2 | 0.519 (0.136) | 0.623 (0.114) | 0.617 (0.112) | 0.575 (0.115) |
| qnQEEG_mean_delta_W_Q3 | 0.504 (0.132) | 0.572 (0.107) | 0.574 (0.111) | 0.526 (0.108) |
| qnQEEG_mean_delta_W_Q4 | 0.504 (0.129) | 0.546 (0.095) | 0.532 (0.107) | 0.489 (0.123) |
| qnQEEG_mean_gamma_N1_Q1 | 0.030 (0.026) | 0.031 (0.024) | 0.021 (0.017) | 0.025 (0.015) |
| qnQEEG_mean_gamma_N1_Q2 | 0.028 (0.020) | 0.028 (0.035) | 0.020 (0.018) | 0.023 (0.017) |
| qnQEEG_mean_gamma_N1_Q3 | 0.028 (0.021) | 0.026 (0.028) | 0.019 (0.018) | 0.028 (0.041) |
| qnQEEG_mean_gamma_N1_Q4 | 0.028 (0.021) | 0.025 (0.019) | 0.022 (0.018) | 0.021 (0.010) |
| qnQEEG_mean_gamma_N2_Q1 | 0.014 (0.014) | 0.011 (0.008) | 0.008 (0.005) | 0.009 (0.007) |
| qnQEEG_mean_gamma_N2_Q2 | 0.015 (0.026) | 0.010 (0.008) | 0.006 (0.004) | 0.008 (0.007) |
| qnQEEG_mean_gamma_N2_Q3 | 0.015 (0.023) | 0.010 (0.011) | 0.006 (0.004) | 0.008 (0.004) |
| qnQEEG_mean_gamma_N2_Q4 | 0.015 (0.025) | 0.010 (0.008) | 0.007 (0.005) | 0.008 (0.005) |
| qnQEEG_mean_gamma_N3_Q1 | 0.003 (0.006) | 0.002 (0.005) | 0.001 (0.001) | 0.004 (0.014) |
| qnQEEG_mean_gamma_N3_Q2 | 0.005 (0.021) | 0.002 (0.001) | 0.001 (0.001) | 0.003 (0.007) |
| qnQEEG_mean_gamma_N3_Q3 | 0.004 (0.005) | 0.002 (0.002) | 0.003 (0.007) | 0.002 (0.005) |
| qnQEEG_mean_gamma_N3_Q4 | 0.005 (0.025) | 0.003 (0.002) | 0.002 (0.002) | 0.003 (0.008) |
| qnQEEG_mean_gamma_REM_Q1 | 0.017 (0.012) | 0.012 (0.009) | 0.008 (0.006) | 0.009 (0.005) |
| qnQEEG_mean_gamma_REM_Q2 | 0.015 (0.011) | 0.012 (0.011) | 0.009 (0.006) | 0.008 (0.003) |
| qnQEEG_mean_gamma_REM_Q3 | 0.016 (0.011) | 0.013 (0.009) | 0.009 (0.005) | 0.009 (0.004) |
| qnQEEG_mean_gamma_REM_Q4 | 0.017 (0.011) | 0.014 (0.009) | 0.010 (0.005) | 0.012 (0.011) |
| qnQEEG_mean_gamma_W_Q1 | 0.030 (0.029) | 0.022 (0.024) | 0.013 (0.012) | 0.021 (0.020) |
| qnQEEG_mean_gamma_W_Q2 | 0.034 (0.037) | 0.021 (0.022) | 0.013 (0.014) | 0.022 (0.021) |
| qnQEEG_mean_gamma_W_Q3 | 0.036 (0.034) | 0.022 (0.025) | 0.015 (0.016) | 0.023 (0.020) |
| qnQEEG_mean_gamma_W_Q4 | 0.032 (0.032) | 0.025 (0.023) | 0.018 (0.015) | 0.022 (0.019) |
| qnQEEG_mean_sigma_N1_Q1 | 0.054 (0.026) | 0.056 (0.021) | 0.060 (0.025) | 0.069 (0.025) |
| qnQEEG_mean_sigma_N1_Q2 | 0.052 (0.025) | 0.053 (0.022) | 0.050 (0.022) | 0.058 (0.023) |
| qnQEEG_mean_sigma_N1_Q3 | 0.054 (0.023) | 0.052 (0.022) | 0.055 (0.021) | 0.063 (0.022) |
| qnQEEG_mean_sigma_N1_Q4 | 0.050 (0.022) | 0.053 (0.022) | 0.053 (0.024) | 0.067 (0.029) |
| qnQEEG_mean_sigma_N2_Q1 | 0.058 (0.025) | 0.056 (0.017) | 0.059 (0.021) | 0.058 (0.019) |
| qnQEEG_mean_sigma_N2_Q2 | 0.062 (0.027) | 0.060 (0.021) | 0.062 (0.023) | 0.062 (0.023) |
| qnQEEG_mean_sigma_N2_Q3 | 0.066 (0.028) | 0.065 (0.023) | 0.072 (0.028) | 0.068 (0.022) |
| qnQEEG_mean_sigma_N2_Q4 | 0.063 (0.029) | 0.070 (0.024) | 0.075 (0.029) | 0.073 (0.023) |
| qnQEEG_mean_sigma_N3_Q1 | 0.025 (0.014) | 0.023 (0.011) | 0.024 (0.013) | 0.027 (0.014) |
| qnQEEG_mean_sigma_N3_Q2 | 0.028 (0.018) | 0.023 (0.012) | 0.022 (0.009) | 0.028 (0.019) |
| qnQEEG_mean_sigma_N3_Q3 | 0.028 (0.019) | 0.030 (0.013) | 0.033 (0.017) | 0.028 (0.018) |
| qnQEEG_mean_sigma_N3_Q4 | 0.032 (0.020) | 0.034 (0.020) | 0.040 (0.022) | 0.035 (0.018) |
| qnQEEG_mean_sigma_REM_Q1 | 0.047 (0.020) | 0.047 (0.018) | 0.045 (0.017) | 0.048 (0.023) |
| qnQEEG_mean_sigma_REM_Q2 | 0.045 (0.021) | 0.042 (0.018) | 0.042 (0.015) | 0.048 (0.018) |
| qnQEEG_mean_sigma_REM_Q3 | 0.044 (0.021) | 0.040 (0.016) | 0.041 (0.015) | 0.046 (0.018) |
| qnQEEG_mean_sigma_REM_Q4 | 0.042 (0.018) | 0.041 (0.015) | 0.041 (0.015) | 0.047 (0.017) |
| qnQEEG_mean_sigma_W_Q1 | 0.048 (0.021) | 0.047 (0.024) | 0.047 (0.024) | 0.051 (0.030) |
| qnQEEG_mean_sigma_W_Q2 | 0.052 (0.024) | 0.048 (0.020) | 0.049 (0.022) | 0.056 (0.023) |
| qnQEEG_mean_sigma_W_Q3 | 0.052 (0.022) | 0.053 (0.023) | 0.053 (0.022) | 0.061 (0.023) |
| qnQEEG_mean_sigma_W_Q4 | 0.049 (0.021) | 0.055 (0.019) | 0.057 (0.025) | 0.062 (0.027) |
| qnQEEG_mean_theta_N1_Q1 | 0.206 (0.063) | 0.191 (0.046) | 0.203 (0.054) | 0.192 (0.041) |
| qnQEEG_mean_theta_N1_Q2 | 0.207 (0.066) | 0.183 (0.044) | 0.193 (0.057) | 0.179 (0.056) |
| qnQEEG_mean_theta_N1_Q3 | 0.215 (0.059) | 0.195 (0.050) | 0.195 (0.050) | 0.191 (0.039) |
| qnQEEG_mean_theta_N1_Q4 | 0.219 (0.053) | 0.200 (0.045) | 0.201 (0.051) | 0.201 (0.043) |
| qnQEEG_mean_theta_N2_Q1 | 0.198 (0.046) | 0.193 (0.035) | 0.199 (0.035) | 0.196 (0.034) |
| qnQEEG_mean_theta_N2_Q2 | 0.197 (0.051) | 0.181 (0.033) | 0.186 (0.035) | 0.186 (0.033) |
| qnQEEG_mean_theta_N2_Q3 | 0.203 (0.045) | 0.188 (0.037) | 0.192 (0.036) | 0.201 (0.028) |
| qnQEEG_mean_theta_N2_Q4 | 0.214 (0.042) | 0.201 (0.033) | 0.204 (0.035) | 0.210 (0.033) |
| qnQEEG_mean_theta_N3_Q1 | 0.124 (0.034) | 0.121 (0.031) | 0.118 (0.030) | 0.131 (0.029) |
| qnQEEG_mean_theta_N3_Q2 | 0.135 (0.045) | 0.116 (0.033) | 0.116 (0.033) | 0.130 (0.029) |
| qnQEEG_mean_theta_N3_Q3 | 0.132 (0.045) | 0.137 (0.037) | 0.133 (0.037) | 0.135 (0.036) |
| qnQEEG_mean_theta_N3_Q4 | 0.136 (0.044) | 0.146 (0.047) | 0.150 (0.042) | 0.160 (0.038) |
| qnQEEG_mean_theta_REM_Q1 | 0.242 (0.057) | 0.234 (0.044) | 0.247 (0.049) | 0.240 (0.037) |
| qnQEEG_mean_theta_REM_Q2 | 0.230 (0.048) | 0.233 (0.041) | 0.243 (0.044) | 0.246 (0.037) |
| qnQEEG_mean_theta_REM_Q3 | 0.236 (0.044) | 0.237 (0.039) | 0.247 (0.046) | 0.248 (0.037) |
| qnQEEG_mean_theta_REM_Q4 | 0.239 (0.044) | 0.239 (0.039) | 0.253 (0.040) | 0.244 (0.042) |
| qnQEEG_mean_theta_W_Q1 | 0.178 (0.056) | 0.161 (0.045) | 0.162 (0.050) | 0.151 (0.046) |
| qnQEEG_mean_theta_W_Q2 | 0.185 (0.054) | 0.159 (0.040) | 0.174 (0.049) | 0.167 (0.041) |
| qnQEEG_mean_theta_W_Q3 | 0.189 (0.052) | 0.182 (0.040) | 0.187 (0.046) | 0.176 (0.039) |
| qnQEEG_mean_theta_W_Q4 | 0.202 (0.052) | 0.189 (0.043) | 0.195 (0.049) | 0.191 (0.044) |
| wnSPIND_spindle_W_AMP_all | 21.7 (5.75) | 22.0 (5.94) | 23.7 (6.10) | 23.4 (5.00) |
| wnSPIND_spindle_W_COUPL_ANGLE_all | –44.000 (80.0) | –55.859 (56.3) | –50.390 (68.3) | –71.495 (42.9) |
| wnSPIND_spindle_W_COUPL_MAG_all | 0.223 (0.136) | 0.228 (0.104) | 0.216 (0.105) | 0.238 (0.108) |
| wnSPIND_spindle_W_COUPL_OVERLAP_PCT_all | 0.331 (0.070) | 0.334 (0.072) | 0.340 (0.066) | 0.342 (0.063) |
| wnSPIND_spindle_W_DENS_all | 5.58 (2.01) | 6.14 (1.85) | 6.41 (1.78) | 6.16 (1.68) |
| wnSPIND_spindle_W_DISPERSION_all | 1.22 (0.457) | 1.11 (0.254) | 1.15 (0.239) | 1.19 (0.201) |
| wnSPIND_spindle_W_FRQ_all | 12.6 (0.320) | 12.7 (0.282) | 12.9 (0.317) | 12.8 (0.272) |
| wnSPIND_spindle_W_FWHM_all | 0.419 (0.045) | 0.426 (0.049) | 0.421 (0.045) | 0.418 (0.034) |
| wnSPIND_spindle_W_N_all | 1082 (495) | 1405 (542) | 1539 (587) | 1424 (497) |
| wnSPIND_spindle_W_Q_all | 0.593 (0.058) | 0.595 (0.049) | 0.595 (0.056) | 0.608 (0.066) |

BMI, body mass index; IH, idiopathic hypersomnia; NT1, narcolepsy type 1; NT2, narcolepsy type 2; Prop, proportion; REM, rapid eye movement; ssi, stage shift index; tst, total sleep time (min); "XtoY", transition from stage X to stage Y. The sleep feature names are abbreviated as follows: the prefix represents the feature set that the feature originates from: wnSM is whole-night hypnogram features; qnSM is quarter-night hypnogram features; qnTP is quarter-night stage transition probability features; qnHYPN is quarter-night hypnodensity features; qnQEEG is quarter-night quantitative electroencephalogram features; wnSPIND is whole-night spindle features. The suffix represents the time resolution of the feature: all is whole night; Q1/2/3/4 is quarter 1/2/3/4 of the night.

**Supplementary Table S3.** Difference in means for pairs of diagnoses (NT2 versus clinical controls, NT2 versus IH, IH versus clinical controls), expressed as a percentage relative to the second diagnosis in the pair, along with the original (uncorrected) *p-*values from a *t*-test on the difference, for demographics and sleep features.

| **Feature name** | **Difference in means, expressed as a percentage relative to the second diagnosis in each pair (*t*-test *p-*value)** | | |
| --- | --- | --- | --- |
|  | **NT2 vs Clinical controls** | **NT2 vs IH** | **IH vs Clinical controls** |
| Age (not used in diagnosis classification) | -13% (4.1e-02*) | -1% (8.9e-01) | -12% (4.4e-02*) |
| BMI (not used in diagnosis classification) | -2% (8.0e-01) | 4% (1.5e-01) | -6% (3.8e-01) |
| Sex (prop. male) (not used in diagnosis classification) | 11% (5.8e-01) | 138% (1.6e-06*^) | -54% (1.4e-03*) |
| Orexin-A (pg/mL) (not used in diagnosis classification) | -17% (9.7e-02) | 4% (5.8e-01) | -20% (6.0e-02) |
| wnSM_REMonSet | -38% (5.0e-05*^) | -32% (2.4e-04*^) | -10% (3.3e-01) |
| wnSM_propN1_all | -10% (3.8e-01) | 9% (3.5e-01) | -17% (7.2e-02) |
| wnSM_propN2_all | -4% (1.7e-01) | -3% (2.1e-01) | -1% (7.1e-01) |
| wnSM_propN3_all | 1% (9.2e-01) | -1% (8.7e-01) | 1% (8.1e-01) |
| wnSM_propR_all | 28% (5.2e-05*^) | 11% (8.2e-03*) | 15% (1.8e-02*) |
| wnSM_propW_all | -26% (1.2e-02*) | -11% (4.8e-01) | -17% (3.5e-01) |
| wnSM_ssi_all | -5% (4.3e-01) | 15% (6.8e-03*) | -17% (5.2e-04*^) |
| wnSM_tst_all | 11% (1.6e-05*^) | -0% (9.6e-01) | 11% (8.9e-04*^) |
| qnSM_propN1_Q1 | 1% (9.5e-01) | 2% (8.8e-01) | -1% (9.6e-01) |
| qnSM_propN1_Q2 | -12% (4.7e-01) | 12% (4.2e-01) | -21% (1.3e-01) |
| qnSM_propN1_Q3 | -15% (2.7e-01) | 7% (5.8e-01) | -21% (9.8e-02) |
| qnSM_propN1_Q4 | -12% (3.5e-01) | 16% (1.9e-01) | -24% (3.4e-02*) |
| qnSM_propN2_Q1 | -10% (4.0e-02*) | -3% (4.5e-01) | -7% (1.6e-01) |
| qnSM_propN2_Q2 | -8% (1.2e-01) | -4% (3.5e-01) | -4% (4.5e-01) |
| qnSM_propN2_Q3 | 1% (8.8e-01) | -6% (1.4e-01) | 7% (2.1e-01) |
| qnSM_propN2_Q4 | -1% (8.0e-01) | -0% (9.4e-01) | -1% (8.3e-01) |
| qnSM_propN3_Q1 | 5% (5.0e-01) | -0% (9.5e-01) | 6% (4.7e-01) |
| qnSM_propN3_Q2 | 4% (7.6e-01) | -4% (6.9e-01) | 8% (5.2e-01) |
| qnSM_propN3_Q3 | -15% (3.2e-01) | 11% (3.9e-01) | -24% (8.7e-02) |
| qnSM_propN3_Q4 | -3% (9.1e-01) | -11% (4.8e-01) | 10% (6.6e-01) |
| qnSM_propR_Q1 | 59% (9.1e-03*) | 22% (6.9e-02) | 30% (1.6e-01) |
| qnSM_propR_Q2 | 29% (8.6e-03*) | 15% (4.1e-02*) | 12% (2.4e-01) |
| qnSM_propR_Q3 | 21% (3.8e-02*) | 10% (1.5e-01) | 10% (2.7e-01) |
| qnSM_propR_Q4 | 23% (1.1e-02*) | 7% (2.6e-01) | 15% (9.6e-02) |
| qnSM_propW_Q1 | -23% (2.3e-01) | -12% (6.0e-01) | -13% (6.3e-01) |
| qnSM_propW_Q2 | -17% (3.1e-01) | -7% (7.8e-01) | -11% (7.1e-01) |
| qnSM_propW_Q3 | -24% (1.9e-01) | -9% (7.0e-01) | -17% (5.0e-01) |
| qnSM_propW_Q4 | -35% (4.8e-03*) | -16% (2.9e-01) | -23% (1.5e-01) |
| qnSM_ssi_Q1 | -2% (7.9e-01) | 10% (1.5e-01) | -11% (1.5e-01) |
| qnSM_ssi_Q2 | -6% (4.3e-01) | 17% (1.3e-02*) | -20% (2.5e-03*) |
| qnSM_ssi_Q3 | -4% (6.9e-01) | 17% (1.8e-02*) | -18% (1.6e-02*) |
| qnSM_ssi_Q4 | -8% (3.0e-01) | 15% (2.9e-02*) | -20% (1.2e-03*^) |
| qnSM_tst_Q1 | 10% (5.5e-04*^) | -1% (8.3e-01) | 11% (8.5e-03*) |
| qnSM_tst_Q2 | 10% (3.5e-04*^) | -1% (7.2e-01) | 11% (1.0e-02*) |
| qnSM_tst_Q3 | 11% (3.3e-04*^) | -0% (9.4e-01) | 12% (3.3e-03*) |
| qnSM_tst_Q4 | 13% (4.0e-06*^) | 1% (5.5e-01) | 12% (9.1e-05*^) |
| qnTM_N1toN1_Q1 | 12% (4.2e-01) | -3% (7.5e-01) | 15% (2.7e-01) |
| qnTM_N1toN1_Q2 | 25% (2.7e-01) | -0% (9.7e-01) | 25% (2.4e-01) |
| qnTM_N1toN1_Q3 | -18% (1.9e-01) | -1% (9.6e-01) | -17% (2.1e-01) |
| qnTM_N1toN1_Q4 | -1% (9.4e-01) | -2% (8.2e-01) | 1% (9.1e-01) |
| qnTM_N1toN2_Q1 | -14% (1.1e-01) | -8% (2.5e-01) | -6% (4.8e-01) |
| qnTM_N1toN2_Q2 | -4% (7.6e-01) | 1% (9.6e-01) | -4% (7.1e-01) |
| qnTM_N1toN2_Q3 | -8% (3.9e-01) | -5% (5.3e-01) | -3% (7.4e-01) |
| qnTM_N1toN2_Q4 | 9% (4.4e-01) | -8% (2.9e-01) | 19% (7.8e-02) |
| qnTM_N1toN3_Q1 | n/a | n/a | n/a |
| qnTM_N1toN3_Q2 | n/a | -100% (2.2e-01) | n/a |
| qnTM_N1toN3_Q3 | n/a | n/a | n/a |
| qnTM_N1toN3_Q4 | n/a | n/a | n/a |
| qnTM_N1toR_Q1 | 273% (3.8e-02*) | 152% (1.6e-02*) | 48% (5.5e-01) |
| qnTM_N1toR_Q2 | -24% (5.2e-01) | 50% (2.5e-01) | -49% (4.0e-02*) |
| qnTM_N1toR_Q3 | 83% (1.4e-01) | 76% (4.1e-02*) | 4% (9.1e-01) |
| qnTM_N1toR_Q4 | 1% (9.6e-01) | -1% (9.6e-01) | 3% (9.4e-01) |
| qnTM_N1toW_Q1 | 8% (8.2e-01) | 3% (9.1e-01) | 5% (8.8e-01) |
| qnTM_N1toW_Q2 | -8% (8.1e-01) | -10% (7.0e-01) | 3% (9.3e-01) |
| qnTM_N1toW_Q3 | 4% (8.9e-01) | 13% (6.0e-01) | -8% (7.8e-01) |
| qnTM_N1toW_Q4 | -37% (1.7e-02*) | -7% (6.9e-01) | -32% (5.1e-02) |
| qnTM_N2toN1_Q1 | 16% (5.8e-01) | 45% (7.1e-02) | -20% (3.8e-01) |
| qnTM_N2toN1_Q2 | 20% (5.5e-01) | 94% (1.2e-02*) | -38% (1.3e-01) |
| qnTM_N2toN1_Q3 | -18% (4.2e-01) | 37% (2.3e-01) | -40% (9.3e-02) |
| qnTM_N2toN1_Q4 | 2% (9.5e-01) | 26% (3.0e-01) | -20% (4.1e-01) |
| qnTM_N2toN2_Q1 | -1% (5.1e-01) | -2% (3.4e-02*) | 1% (2.9e-01) |
| qnTM_N2toN2_Q2 | -1% (3.4e-01) | -0% (9.0e-01) | -1% (7.7e-01) |
| qnTM_N2toN2_Q3 | 0% (9.5e-01) | -1% (6.3e-01) | 1% (7.0e-01) |
| qnTM_N2toN2_Q4 | 1% (4.5e-01) | -1% (1.9e-01) | 1% (7.2e-02) |
| qnTM_N2toN3_Q1 | 11% (5.4e-01) | 11% (3.3e-01) | -1% (9.4e-01) |
| qnTM_N2toN3_Q2 | 1% (9.7e-01) | 27% (1.8e-01) | -20% (2.0e-01) |
| qnTM_N2toN3_Q3 | 29% (2.3e-01) | 58% (3.6e-03*) | -19% (2.5e-01) |
| qnTM_N2toN3_Q4 | -0% (1.0e+00) | 27% (2.1e-01) | -21% (2.7e-01) |
| qnTM_N2toR_Q1 | 85% (1.7e-02*) | 60% (2.2e-03*) | 16% (4.6e-01) |
| qnTM_N2toR_Q2 | 81% (1.4e-02*) | 41% (1.8e-02*) | 29% (1.1e-01) |
| qnTM_N2toR_Q3 | 15% (4.7e-01) | 34% (6.4e-02) | -14% (4.3e-01) |
| qnTM_N2toR_Q4 | -3% (8.6e-01) | 18% (1.0e-01) | -18% (1.9e-01) |
| qnTM_N2toW_Q1 | -22% (1.7e-01) | -5% (7.3e-01) | -17% (2.7e-01) |
| qnTM_N2toW_Q2 | -8% (5.1e-01) | 14% (2.4e-01) | -19% (1.1e-01) |
| qnTM_N2toW_Q3 | -12% (5.2e-01) | 7% (7.2e-01) | -18% (3.9e-01) |
| qnTM_N2toW_Q4 | -14% (2.4e-01) | -0% (9.7e-01) | -13% (3.0e-01) |
| qnTM_N3toN1_Q1 | -82% (7.5e-02) | -68% (4.2e-01) | -43% (5.9e-01) |
| qnTM_N3toN1_Q2 | n/a | 43% (6.8e-01) | n/a |
| qnTM_N3toN1_Q3 | -65% (2.9e-01) | -44% (7.0e-01) | -38% (7.3e-01) |
| qnTM_N3toN1_Q4 | -69% (2.6e-01) | n/a | -100% (4.7e-02*) |
| qnTM_N3toN2_Q1 | 28% (3.4e-01) | 14% (4.9e-01) | 13% (5.8e-01) |
| qnTM_N3toN2_Q2 | -36% (2.4e-01) | 24% (3.5e-01) | -49% (7.3e-02) |
| qnTM_N3toN2_Q3 | 39% (3.9e-01) | 59% (1.2e-01) | -13% (6.8e-01) |
| qnTM_N3toN2_Q4 | 60% (3.3e-01) | 43% (3.4e-01) | 12% (8.3e-01) |
| qnTM_N3toN3_Q1 | -1% (6.7e-01) | 1% (6.2e-01) | -2% (4.5e-01) |
| qnTM_N3toN3_Q2 | 0% (9.8e-01) | -6% (1.2e-01) | 7% (1.9e-01) |
| qnTM_N3toN3_Q3 | -5% (6.2e-01) | 2% (7.8e-01) | -7% (4.7e-01) |
| qnTM_N3toN3_Q4 | -7% (6.6e-01) | -8% (4.9e-01) | 1% (9.2e-01) |
| qnTM_N3toR_Q1 | 105% (4.9e-01) | -10% (8.9e-01) | 129% (5.9e-01) |
| qnTM_N3toR_Q2 | 37% (7.0e-01) | 117% (2.9e-01) | -37% (6.1e-01) |
| qnTM_N3toR_Q3 | -66% (3.8e-01) | 42% (7.7e-01) | -76% (3.0e-01) |
| qnTM_N3toR_Q4 | n/a | 2556% (1.2e-01) | n/a |
| qnTM_N3toW_Q1 | -37% (8.6e-02) | -19% (2.7e-01) | -22% (1.8e-01) |
| qnTM_N3toW_Q2 | -45% (5.4e-02) | 18% (4.2e-01) | -53% (5.7e-03*) |
| qnTM_N3toW_Q3 | -41% (3.1e-01) | -42% (4.4e-02*) | 1% (9.8e-01) |
| qnTM_N3toW_Q4 | 34% (7.3e-01) | 39% (5.5e-01) | -4% (9.4e-01) |
| qnTM_RtoN1_Q1 | -14% (7.5e-01) | -23% (5.5e-01) | 12% (8.4e-01) |
| qnTM_RtoN1_Q2 | -37% (2.1e-01) | 9% (7.9e-01) | -43% (1.4e-01) |
| qnTM_RtoN1_Q3 | -11% (7.5e-01) | 24% (4.2e-01) | -28% (3.0e-01) |
| qnTM_RtoN1_Q4 | 10% (7.6e-01) | 38% (1.5e-01) | -20% (4.4e-01) |
| qnTM_RtoN2_Q1 | -4% (9.1e-01) | 66% (3.9e-02*) | -42% (1.3e-01) |
| qnTM_RtoN2_Q2 | 58% (7.7e-02) | 16% (5.2e-01) | 36% (4.1e-01) |
| qnTM_RtoN2_Q3 | 208% (2.0e-01) | 102% (1.3e-01) | 52% (2.1e-01) |
| qnTM_RtoN2_Q4 | 21% (5.7e-01) | 20% (4.3e-01) | 1% (9.8e-01) |
| qnTM_RtoN3_Q1 | n/a | -100% (3.6e-01) | n/a |
| qnTM_RtoN3_Q2 | n/a | n/a | n/a |
| qnTM_RtoN3_Q3 | n/a | n/a | n/a |
| qnTM_RtoN3_Q4 | n/a | -14% (9.2e-01) | n/a |
| qnTM_RtoR_Q1 | 51% (1.1e-04*^) | 8% (2.3e-01) | 40% (4.9e-03*) |
| qnTM_RtoR_Q2 | 10% (5.8e-03*) | 2% (3.2e-01) | 8% (7.4e-02) |
| qnTM_RtoR_Q3 | 1% (7.3e-01) | 1% (7.3e-01) | 0% (9.7e-01) |
| qnTM_RtoR_Q4 | 7% (4.0e-02*) | -0% (8.3e-01) | 8% (3.5e-02*) |
| qnTM_RtoW_Q1 | 10% (7.9e-01) | 5% (8.6e-01) | 5% (9.1e-01) |
| qnTM_RtoW_Q2 | 25% (3.4e-01) | 19% (3.4e-01) | 5% (8.4e-01) |
| qnTM_RtoW_Q3 | -11% (5.9e-01) | 10% (5.3e-01) | -19% (2.0e-01) |
| qnTM_RtoW_Q4 | -14% (3.0e-01) | 18% (2.0e-01) | -27% (2.2e-02*) |
| qnTM_WtoN1_Q1 | 19% (3.4e-01) | -2% (9.0e-01) | 21% (2.9e-01) |
| qnTM_WtoN1_Q2 | 1% (9.5e-01) | -23% (3.2e-02*) | 31% (9.2e-02) |
| qnTM_WtoN1_Q3 | 8% (6.0e-01) | -3% (7.7e-01) | 12% (4.6e-01) |
| qnTM_WtoN1_Q4 | -1% (9.5e-01) | -9% (3.0e-01) | 9% (5.0e-01) |
| qnTM_WtoN2_Q1 | -7% (7.2e-01) | -10% (4.7e-01) | 4% (8.4e-01) |
| qnTM_WtoN2_Q2 | 3% (8.7e-01) | 2% (8.8e-01) | 1% (9.7e-01) |
| qnTM_WtoN2_Q3 | 28% (2.5e-01) | -1% (9.5e-01) | 29% (2.1e-01) |
| qnTM_WtoN2_Q4 | 69% (2.7e-02*) | 21% (2.3e-01) | 39% (2.1e-01) |
| qnTM_WtoN3_Q1 | n/a | -18% (7.9e-01) | n/a |
| qnTM_WtoN3_Q2 | n/a | -66% (3.3e-01) | n/a |
| qnTM_WtoN3_Q3 | -32% (8.0e-01) | n/a | -100% (1.1e-01) |
| qnTM_WtoN3_Q4 | n/a | 746% (3.5e-01) | n/a |
| qnTM_WtoR_Q1 | 586% (3.0e-02*) | 134% (3.5e-02*) | 194% (1.9e-01) |
| qnTM_WtoR_Q2 | 127% (8.2e-02) | 49% (2.0e-01) | 53% (3.9e-01) |
| qnTM_WtoR_Q3 | 68% (1.5e-01) | -6% (7.5e-01) | 79% (7.2e-02) |
| qnTM_WtoR_Q4 | 71% (8.5e-02) | 15% (4.7e-01) | 49% (1.8e-01) |
| qnTM_WtoW_Q1 | -27% (2.1e-02*) | 4% (7.7e-01) | -29% (1.0e-02*) |
| qnTM_WtoW_Q2 | -16% (1.3e-01) | 15% (1.9e-01) | -27% (1.2e-02*) |
| qnTM_WtoW_Q3 | -24% (3.1e-02*) | 5% (6.5e-01) | -28% (1.2e-02*) |
| qnTM_WtoW_Q4 | -23% (2.0e-02*) | -4% (6.5e-01) | -20% (5.0e-02*) |
| qnHYPN_N1N2_Q1 | 6% (6.2e-01) | -5% (5.0e-01) | 12% (2.6e-01) |
| qnHYPN_N1N2_Q2 | 20% (1.5e-01) | 7% (4.5e-01) | 12% (3.0e-01) |
| qnHYPN_N1N2_Q3 | 15% (2.7e-01) | -3% (7.1e-01) | 19% (1.1e-01) |
| qnHYPN_N1N2_Q4 | 8% (5.2e-01) | -7% (3.7e-01) | 16% (1.3e-01) |
| qnHYPN_N1N3_Q1 | 53% (1.2e-01) | -15% (3.6e-01) | 80% (1.6e-02*) |
| qnHYPN_N1N3_Q2 | 71% (4.3e-02*) | -27% (8.6e-02) | 134% (3.8e-03*) |
| qnHYPN_N1N3_Q3 | 90% (6.2e-02) | -22% (2.9e-01) | 144% (2.2e-02*) |
| qnHYPN_N1N3_Q4 | 48% (2.0e-01) | -16% (3.5e-01) | 77% (3.0e-02*) |
| qnHYPN_N1R_Q1 | 39% (6.6e-02) | 24% (6.4e-02) | 12% (5.0e-01) |
| qnHYPN_N1R_Q2 | 1% (9.5e-01) | 17% (1.1e-01) | -14% (2.2e-01) |
| qnHYPN_N1R_Q3 | -1% (9.3e-01) | 3% (7.3e-01) | -4% (7.2e-01) |
| qnHYPN_N1R_Q4 | 1% (9.1e-01) | 9% (3.1e-01) | -7% (4.9e-01) |
| qnHYPN_N1_Q1 | 10% (4.3e-01) | -5% (5.9e-01) | 15% (2.1e-01) |
| qnHYPN_N1_Q2 | 20% (1.7e-01) | 8% (4.4e-01) | 11% (3.3e-01) |
| qnHYPN_N1_Q3 | 15% (2.5e-01) | -3% (7.4e-01) | 18% (1.0e-01) |
| qnHYPN_N1_Q4 | 10% (4.2e-01) | -2% (8.1e-01) | 12% (2.3e-01) |
| qnHYPN_N2N3_Q1 | -7% (3.6e-01) | 7% (3.7e-01) | -13% (1.0e-01) |
| qnHYPN_N2N3_Q2 | -14% (2.0e-01) | -10% (2.7e-01) | -5% (6.3e-01) |
| qnHYPN_N2N3_Q3 | -9% (4.9e-01) | -4% (7.0e-01) | -5% (7.1e-01) |
| qnHYPN_N2N3_Q4 | -11% (5.3e-01) | -15% (2.6e-01) | 5% (8.0e-01) |
| qnHYPN_N2R_Q1 | 36% (1.3e-01) | 54% (2.0e-03*) | -12% (4.3e-01) |
| qnHYPN_N2R_Q2 | 25% (1.3e-01) | 42% (1.6e-03*) | -12% (4.0e-01) |
| qnHYPN_N2R_Q3 | 18% (3.0e-01) | 40% (6.1e-03*) | -16% (2.2e-01) |
| qnHYPN_N2R_Q4 | 11% (5.3e-01) | 26% (5.8e-02) | -13% (3.7e-01) |
| qnHYPN_N2_Q1 | -12% (1.5e-02*) | -2% (7.1e-01) | -10% (4.1e-02*) |
| qnHYPN_N2_Q2 | -11% (3.4e-03*) | -2% (6.7e-01) | -10% (2.3e-02*) |
| qnHYPN_N2_Q3 | -8% (1.3e-01) | -7% (9.2e-02) | -1% (8.2e-01) |
| qnHYPN_N2_Q4 | -7% (1.5e-01) | -3% (3.5e-01) | -4% (4.0e-01) |
| qnHYPN_N3R_Q1 | 107% (4.9e-01) | 430% (7.1e-02) | -61% (1.3e-01) |
| qnHYPN_N3R_Q2 | 119% (3.0e-01) | 140% (7.8e-02) | -9% (7.3e-01) |
| qnHYPN_N3R_Q3 | 128% (1.1e-01) | 93% (5.0e-02) | 18% (6.6e-01) |
| qnHYPN_N3R_Q4 | 39% (3.7e-01) | 5% (8.8e-01) | 32% (5.3e-01) |
| qnHYPN_N3_Q1 | 0% (9.8e-01) | -3% (6.8e-01) | 4% (7.3e-01) |
| qnHYPN_N3_Q2 | -6% (6.7e-01) | -9% (4.1e-01) | 3% (8.2e-01) |
| qnHYPN_N3_Q3 | -21% (2.1e-01) | 0% (9.9e-01) | -22% (1.9e-01) |
| qnHYPN_N3_Q4 | -17% (4.5e-01) | -14% (4.6e-01) | -5% (8.4e-01) |
| qnHYPN_R_Q1 | 77% (7.3e-03*) | 47% (4.1e-03*) | 20% (3.5e-01) |
| qnHYPN_R_Q2 | 30% (3.6e-02*) | 28% (7.2e-03*) | 1% (9.4e-01) |
| qnHYPN_R_Q3 | 20% (8.3e-02) | 24% (6.5e-03*) | -4% (7.2e-01) |
| qnHYPN_R_Q4 | 18% (9.7e-02) | 21% (1.9e-02*) | -2% (8.2e-01) |
| qnHYPN_WN1_Q1 | 7% (6.8e-01) | -6% (6.4e-01) | 14% (4.1e-01) |
| qnHYPN_WN1_Q2 | 36% (1.2e-01) | -3% (8.5e-01) | 40% (8.9e-02) |
| qnHYPN_WN1_Q3 | 38% (1.3e-01) | -2% (9.0e-01) | 40% (7.8e-02) |
| qnHYPN_WN1_Q4 | 17% (3.5e-01) | -5% (7.1e-01) | 23% (1.7e-01) |
| qnHYPN_WN2_Q1 | 52% (6.7e-02) | -15% (3.3e-01) | 78% (1.4e-02*) |
| qnHYPN_WN2_Q2 | 63% (4.0e-02*) | -20% (1.9e-01) | 104% (7.5e-03*) |
| qnHYPN_WN2_Q3 | 39% (1.4e-01) | -20% (2.0e-01) | 73% (2.8e-02*) |
| qnHYPN_WN2_Q4 | 44% (7.4e-02) | -17% (2.0e-01) | 75% (7.1e-03*) |
| qnHYPN_WN3_Q1 | 211% (4.4e-02*) | -46% (6.5e-02) | 481% (1.5e-02*) |
| qnHYPN_WN3_Q2 | 175% (3.6e-02*) | -54% (2.6e-02*) | 493% (1.3e-02*) |
| qnHYPN_WN3_Q3 | 104% (1.8e-01) | -40% (1.5e-01) | 241% (5.6e-02) |
| qnHYPN_WN3_Q4 | 170% (1.8e-01) | -42% (2.2e-01) | 365% (7.8e-02) |
| qnHYPN_WR_Q1 | 102% (2.6e-02*) | 65% (9.1e-03*) | 22% (4.9e-01) |
| qnHYPN_WR_Q2 | 43% (5.3e-02) | 20% (1.6e-01) | 19% (3.5e-01) |
| qnHYPN_WR_Q3 | 78% (2.5e-02*) | 36% (5.4e-02) | 31% (1.5e-01) |
| qnHYPN_WR_Q4 | 47% (5.1e-02) | 15% (2.8e-01) | 27% (1.5e-01) |
| qnHYPN_W_Q1 | 1% (9.7e-01) | -11% (4.9e-01) | 13% (5.8e-01) |
| qnHYPN_W_Q2 | 17% (4.1e-01) | -18% (2.7e-01) | 42% (1.6e-01) |
| qnHYPN_W_Q3 | 12% (5.8e-01) | -4% (8.3e-01) | 16% (5.1e-01) |
| qnHYPN_W_Q4 | -3% (8.4e-01) | -13% (3.0e-01) | 11% (5.2e-01) |
| qnHYPN_entropy_Q1 | 15% (2.2e-02*) | 1% (8.4e-01) | 14% (4.5e-02*) |
| qnHYPN_entropy_Q2 | 15% (3.3e-02*) | -2% (7.7e-01) | 17% (3.5e-02*) |
| qnHYPN_entropy_Q3 | 16% (3.8e-02*) | -0% (1.0e+00) | 16% (3.9e-02*) |
| qnHYPN_entropy_Q4 | 11% (1.3e-01) | -3% (6.2e-01) | 14% (5.0e-02) |
| qnQEEG_mean_alpha_N1_Q1 | -5% (5.0e-01) | -1% (8.3e-01) | -4% (5.9e-01) |
| qnQEEG_mean_alpha_N1_Q2 | -22% (9.8e-03*) | -8% (2.3e-01) | -14% (7.8e-02) |
| qnQEEG_mean_alpha_N1_Q3 | -18% (2.2e-02*) | -4% (5.7e-01) | -15% (2.8e-02*) |
| qnQEEG_mean_alpha_N1_Q4 | -21% (3.9e-03*) | -8% (2.2e-01) | -14% (5.5e-02) |
| qnQEEG_mean_alpha_N2_Q1 | -14% (9.1e-03*) | -4% (3.8e-01) | -11% (5.3e-02) |
| qnQEEG_mean_alpha_N2_Q2 | -11% (7.9e-02) | 0% (9.5e-01) | -11% (4.2e-02*) |
| qnQEEG_mean_alpha_N2_Q3 | -13% (2.4e-02*) | -2% (6.1e-01) | -11% (2.5e-02*) |
| qnQEEG_mean_alpha_N2_Q4 | -12% (3.9e-02*) | -4% (3.6e-01) | -8% (1.4e-01) |
| qnQEEG_mean_alpha_N3_Q1 | -15% (6.9e-02) | 6% (4.4e-01) | -19% (1.8e-02*) |
| qnQEEG_mean_alpha_N3_Q2 | -21% (1.2e-02*) | 1% (9.0e-01) | -22% (5.6e-03*) |
| qnQEEG_mean_alpha_N3_Q3 | -2% (8.4e-01) | -2% (7.8e-01) | 0% (9.8e-01) |
| qnQEEG_mean_alpha_N3_Q4 | -17% (9.9e-02) | -10% (2.5e-01) | -8% (4.2e-01) |
| qnQEEG_mean_alpha_REM_Q1 | -3% (6.8e-01) | -5% (3.3e-01) | 2% (7.8e-01) |
| qnQEEG_mean_alpha_REM_Q2 | -11% (8.1e-02) | -10% (5.6e-02) | -2% (8.1e-01) |
| qnQEEG_mean_alpha_REM_Q3 | -12% (3.5e-02*) | -8% (1.0e-01) | -4% (5.0e-01) |
| qnQEEG_mean_alpha_REM_Q4 | -9% (1.0e-01) | -9% (5.8e-02) | -0% (9.7e-01) |
| qnQEEG_mean_alpha_W_Q1 | -14% (1.2e-01) | -3% (7.1e-01) | -12% (2.1e-01) |
| qnQEEG_mean_alpha_W_Q2 | -18% (2.4e-02*) | -4% (5.5e-01) | -15% (5.6e-02) |
| qnQEEG_mean_alpha_W_Q3 | -25% (8.6e-04*^) | -1% (8.5e-01) | -24% (9.2e-04*^) |
| qnQEEG_mean_alpha_W_Q4 | -23% (2.8e-03*) | -8% (2.2e-01) | -16% (4.4e-02*) |
| qnQEEG_mean_beta_N1_Q1 | -2% (8.0e-01) | 4% (5.6e-01) | -6% (4.4e-01) |
| qnQEEG_mean_beta_N1_Q2 | -2% (8.3e-01) | 7% (4.4e-01) | -9% (3.3e-01) |
| qnQEEG_mean_beta_N1_Q3 | -14% (1.1e-01) | 8% (2.9e-01) | -20% (1.5e-02*) |
| qnQEEG_mean_beta_N1_Q4 | -6% (4.4e-01) | -9% (3.3e-01) | 3% (8.0e-01) |
| qnQEEG_mean_beta_N2_Q1 | -0% (9.7e-01) | 5% (4.5e-01) | -5% (5.6e-01) |
| qnQEEG_mean_beta_N2_Q2 | -1% (9.3e-01) | 17% (1.7e-02*) | -15% (5.4e-02) |
| qnQEEG_mean_beta_N2_Q3 | -2% (8.3e-01) | 14% (4.1e-02*) | -14% (3.1e-02*) |
| qnQEEG_mean_beta_N2_Q4 | -4% (5.9e-01) | -3% (7.7e-01) | -1% (9.2e-01) |
| qnQEEG_mean_beta_N3_Q1 | -37% (2.3e-01) | 27% (9.3e-02) | -50% (5.9e-02) |
| qnQEEG_mean_beta_N3_Q2 | -31% (1.4e-01) | 20% (1.2e-02*) | -42% (2.7e-02*) |
| qnQEEG_mean_beta_N3_Q3 | -7% (7.3e-01) | 1% (9.7e-01) | -8% (7.6e-01) |
| qnQEEG_mean_beta_N3_Q4 | -33% (2.5e-01) | -4% (7.2e-01) | -30% (2.5e-01) |
| qnQEEG_mean_beta_REM_Q1 | 1% (9.6e-01) | 4% (5.7e-01) | -4% (7.3e-01) |
| qnQEEG_mean_beta_REM_Q2 | 5% (5.9e-01) | 5% (4.3e-01) | -0% (9.8e-01) |
| qnQEEG_mean_beta_REM_Q3 | 8% (3.4e-01) | 6% (3.6e-01) | 2% (7.6e-01) |
| qnQEEG_mean_beta_REM_Q4 | -4% (6.4e-01) | 8% (1.8e-01) | -11% (1.2e-01) |
| qnQEEG_mean_beta_W_Q1 | -6% (6.8e-01) | 11% (3.9e-01) | -15% (2.9e-01) |
| qnQEEG_mean_beta_W_Q2 | -15% (1.4e-01) | 12% (2.9e-01) | -25% (4.1e-02*) |
| qnQEEG_mean_beta_W_Q3 | -11% (2.5e-01) | 1% (9.2e-01) | -12% (4.5e-01) |
| qnQEEG_mean_beta_W_Q4 | -21% (1.0e-01) | -5% (7.0e-01) | -17% (3.2e-01) |
| qnQEEG_mean_delta_N1_Q1 | 4% (4.0e-01) | 1% (8.1e-01) | 3% (5.1e-01) |
| qnQEEG_mean_delta_N1_Q2 | 6% (2.1e-01) | 1% (7.4e-01) | 5% (2.7e-01) |
| qnQEEG_mean_delta_N1_Q3 | 10% (2.0e-02*) | -1% (7.4e-01) | 11% (6.5e-03*) |
| qnQEEG_mean_delta_N1_Q4 | 10% (1.1e-02*) | 3% (2.7e-01) | 7% (1.0e-01) |
| qnQEEG_mean_delta_N2_Q1 | 3% (1.3e-01) | 1% (4.5e-01) | 2% (3.7e-01) |
| qnQEEG_mean_delta_N2_Q2 | 3% (2.2e-01) | -0% (8.0e-01) | 3% (1.2e-01) |
| qnQEEG_mean_delta_N2_Q3 | 5% (4.6e-02*) | 1% (6.7e-01) | 4% (3.5e-02*) |
| qnQEEG_mean_delta_N2_Q4 | 5% (5.0e-02*) | 2% (2.7e-01) | 3% (3.0e-01) |
| qnQEEG_mean_delta_N3_Q1 | 4% (2.9e-02*) | -1% (3.2e-01) | 5% (2.8e-03*) |
| qnQEEG_mean_delta_N3_Q2 | 4% (8.7e-03*) | -0% (7.1e-01) | 5% (1.2e-03*^) |
| qnQEEG_mean_delta_N3_Q3 | -0% (9.3e-01) | 0% (8.9e-01) | -0% (8.6e-01) |
| qnQEEG_mean_delta_N3_Q4 | 5% (1.3e-01) | 2% (3.2e-01) | 2% (3.7e-01) |
| qnQEEG_mean_delta_REM_Q1 | 1% (6.8e-01) | 2% (3.5e-01) | -1% (8.3e-01) |
| qnQEEG_mean_delta_REM_Q2 | 5% (1.1e-01) | 3% (1.8e-01) | 2% (4.9e-01) |
| qnQEEG_mean_delta_REM_Q3 | 4% (1.1e-01) | 3% (2.3e-01) | 2% (5.4e-01) |
| qnQEEG_mean_delta_REM_Q4 | 4% (8.6e-02) | 3% (1.4e-01) | 1% (6.0e-01) |
| qnQEEG_mean_delta_W_Q1 | 2% (6.1e-01) | -2% (6.2e-01) | 4% (3.7e-01) |
| qnQEEG_mean_delta_W_Q2 | 8% (2.7e-02*) | 1% (7.0e-01) | 7% (4.5e-02*) |
| qnQEEG_mean_delta_W_Q3 | 9% (2.2e-02*) | -0% (9.1e-01) | 9% (1.8e-02*) |
| qnQEEG_mean_delta_W_Q4 | 12% (3.8e-03*) | 3% (3.2e-01) | 9% (3.6e-02*) |
| qnQEEG_mean_gamma_N1_Q1 | 23% (1.6e-01) | 46% (1.2e-03*^) | -16% (2.0e-01) |
| qnQEEG_mean_gamma_N1_Q2 | 21% (4.3e-01) | 38% (7.3e-02) | -12% (4.5e-01) |
| qnQEEG_mean_gamma_N1_Q3 | -6% (7.8e-01) | 35% (5.1e-02) | -31% (9.2e-02) |
| qnQEEG_mean_gamma_N1_Q4 | 21% (1.8e-01) | 13% (2.7e-01) | 7% (6.5e-01) |
| qnQEEG_mean_gamma_N2_Q1 | 25% (1.3e-01) | 47% (2.0e-04*^) | -15% (2.1e-01) |
| qnQEEG_mean_gamma_N2_Q2 | 23% (2.4e-01) | 62% (1.0e-04*^) | -24% (3.8e-02*) |
| qnQEEG_mean_gamma_N2_Q3 | 33% (1.6e-01) | 62% (1.2e-03*^) | -18% (8.8e-02) |
| qnQEEG_mean_gamma_N2_Q4 | 29% (8.1e-02) | 41% (1.0e-03*^) | -9% (4.3e-01) |
| qnQEEG_mean_gamma_N3_Q1 | -37% (4.1e-01) | 87% (3.0e-02*) | -66% (7.2e-02) |
| qnQEEG_mean_gamma_N3_Q2 | -21% (4.8e-01) | 63% (1.1e-05*^) | -52% (5.7e-02) |
| qnQEEG_mean_gamma_N3_Q3 | 6% (8.3e-01) | -11% (7.4e-01) | 19% (7.5e-01) |
| qnQEEG_mean_gamma_N3_Q4 | -14% (7.1e-01) | 32% (9.3e-02) | -35% (3.1e-01) |
| qnQEEG_mean_gamma_REM_Q1 | 32% (1.5e-01) | 41% (6.3e-03*) | -7% (6.4e-01) |
| qnQEEG_mean_gamma_REM_Q2 | 44% (5.3e-02) | 37% (1.2e-02*) | 6% (6.5e-01) |
| qnQEEG_mean_gamma_REM_Q3 | 42% (1.9e-02*) | 39% (1.2e-03*^) | 2% (8.4e-01) |
| qnQEEG_mean_gamma_REM_Q4 | 12% (4.4e-01) | 44% (1.2e-04*^) | -22% (4.2e-02*) |
| qnQEEG_mean_gamma_W_Q1 | 3% (8.8e-01) | 62% (3.0e-03*) | -36% (7.9e-03*) |
| qnQEEG_mean_gamma_W_Q2 | -3% (8.8e-01) | 64% (1.5e-03*) | -41% (2.9e-03*) |
| qnQEEG_mean_gamma_W_Q3 | -4% (8.3e-01) | 45% (2.0e-02*) | -34% (1.2e-02*) |
| qnQEEG_mean_gamma_W_Q4 | 16% (4.0e-01) | 43% (6.3e-03*) | -19% (1.7e-01) |
| qnQEEG_mean_sigma_N1_Q1 | -18% (4.1e-03*) | -6% (2.9e-01) | -13% (6.0e-02) |
| qnQEEG_mean_sigma_N1_Q2 | -9% (2.6e-01) | 5% (4.3e-01) | -13% (8.5e-02) |
| qnQEEG_mean_sigma_N1_Q3 | -17% (9.9e-03*) | -5% (4.2e-01) | -13% (4.0e-02*) |
| qnQEEG_mean_sigma_N1_Q4 | -21% (3.6e-03*) | 0% (9.6e-01) | -21% (3.4e-03*) |
| qnQEEG_mean_sigma_N2_Q1 | -4% (4.4e-01) | -5% (2.7e-01) | 1% (9.0e-01) |
| qnQEEG_mean_sigma_N2_Q2 | -3% (5.9e-01) | -3% (5.9e-01) | -1% (9.2e-01) |
| qnQEEG_mean_sigma_N2_Q3 | -4% (5.5e-01) | -9% (8.0e-02) | 6% (4.2e-01) |
| qnQEEG_mean_sigma_N2_Q4 | -5% (4.6e-01) | -7% (1.6e-01) | 3% (6.8e-01) |
| qnQEEG_mean_sigma_N3_Q1 | -14% (1.1e-01) | -1% (9.2e-01) | -13% (1.6e-01) |
| qnQEEG_mean_sigma_N3_Q2 | -17% (1.1e-01) | 2% (8.0e-01) | -19% (3.3e-02*) |
| qnQEEG_mean_sigma_N3_Q3 | 4% (7.2e-01) | -12% (1.1e-01) | 18% (1.5e-01) |
| qnQEEG_mean_sigma_N3_Q4 | -4% (7.8e-01) | -15% (1.4e-01) | 13% (3.8e-01) |
| qnQEEG_mean_sigma_REM_Q1 | -3% (7.6e-01) | 3% (6.0e-01) | -6% (5.1e-01) |
| qnQEEG_mean_sigma_REM_Q2 | -11% (1.2e-01) | -0% (1.0e+00) | -11% (7.4e-02) |
| qnQEEG_mean_sigma_REM_Q3 | -13% (6.4e-02) | -2% (6.7e-01) | -11% (9.5e-02) |
| qnQEEG_mean_sigma_REM_Q4 | -13% (4.3e-02*) | 0% (9.7e-01) | -14% (2.9e-02*) |
| qnQEEG_mean_sigma_W_Q1 | -9% (3.7e-01) | -1% (9.2e-01) | -8% (4.0e-01) |
| qnQEEG_mean_sigma_W_Q2 | -15% (3.7e-02*) | -2% (7.2e-01) | -13% (8.2e-02) |
| qnQEEG_mean_sigma_W_Q3 | -13% (8.3e-02) | -0% (9.4e-01) | -12% (7.4e-02) |
| qnQEEG_mean_sigma_W_Q4 | -12% (7.9e-02) | -4% (5.1e-01) | -8% (2.8e-01) |
| qnQEEG_mean_theta_N1_Q1 | -0% (9.8e-01) | -6% (1.1e-01) | 6% (2.3e-01) |
| qnQEEG_mean_theta_N1_Q2 | 2% (6.7e-01) | -5% (2.1e-01) | 8% (2.1e-01) |
| qnQEEG_mean_theta_N1_Q3 | 2% (6.7e-01) | -0% (9.8e-01) | 2% (6.5e-01) |
| qnQEEG_mean_theta_N1_Q4 | -1% (8.6e-01) | -1% (8.3e-01) | 0% (1.0e+00) |
| qnQEEG_mean_theta_N2_Q1 | -2% (6.3e-01) | -3% (2.5e-01) | 1% (6.8e-01) |
| qnQEEG_mean_theta_N2_Q2 | -3% (3.9e-01) | -3% (3.1e-01) | -0% (9.7e-01) |
| qnQEEG_mean_theta_N2_Q3 | -6% (5.0e-02*) | -2% (4.6e-01) | -4% (1.6e-01) |
| qnQEEG_mean_theta_N2_Q4 | -4% (1.6e-01) | -2% (5.1e-01) | -3% (3.8e-01) |
| qnQEEG_mean_theta_N3_Q1 | -8% (8.0e-02) | 2% (5.3e-01) | -10% (1.9e-02*) |
| qnQEEG_mean_theta_N3_Q2 | -11% (2.8e-02*) | 0% (9.8e-01) | -11% (2.3e-02*) |
| qnQEEG_mean_theta_N3_Q3 | 1% (8.0e-01) | 3% (5.3e-01) | -1% (8.1e-01) |
| qnQEEG_mean_theta_N3_Q4 | -9% (1.8e-01) | -2% (6.7e-01) | -7% (2.6e-01) |
| qnQEEG_mean_theta_REM_Q1 | -2% (5.5e-01) | -5% (7.7e-02) | 3% (5.0e-01) |
| qnQEEG_mean_theta_REM_Q2 | -5% (9.2e-02) | -4% (1.1e-01) | -1% (6.9e-01) |
| qnQEEG_mean_theta_REM_Q3 | -4% (1.3e-01) | -4% (9.6e-02) | -0% (9.2e-01) |
| qnQEEG_mean_theta_REM_Q4 | -2% (5.4e-01) | -5% (2.1e-02*) | 4% (2.6e-01) |
| qnQEEG_mean_theta_W_Q1 | 6% (2.6e-01) | -1% (8.7e-01) | 7% (2.3e-01) |
| qnQEEG_mean_theta_W_Q2 | -5% (2.7e-01) | -9% (2.0e-02*) | 4% (4.3e-01) |
| qnQEEG_mean_theta_W_Q3 | 3% (4.2e-01) | -2% (4.7e-01) | 6% (1.9e-01) |
| qnQEEG_mean_theta_W_Q4 | -1% (8.1e-01) | -3% (3.3e-01) | 2% (6.1e-01) |
| wnSPIND_spindle_W_AMP_all | -6% (1.8e-01) | -7% (5.4e-02) | 1% (8.3e-01) |
| wnSPIND_spindle_W_COUPL_ANGLE_all | -22% (1.2e-01) | 11% (5.5e-01) | -30% (6.8e-02) |
| wnSPIND_spindle_W_COUPL_MAG_all | -5% (5.9e-01) | 5% (4.3e-01) | -9% (2.5e-01) |
| wnSPIND_spindle_W_COUPL_OVERLAP_PCT_all | -2% (5.3e-01) | -2% (5.3e-01) | -1% (8.7e-01) |
| wnSPIND_spindle_W_DENS_all | -0% (9.5e-01) | -4% (3.0e-01) | 4% (4.4e-01) |
| wnSPIND_spindle_W_DISPERSION_all | -7% (6.5e-02) | -4% (2.5e-01) | -4% (3.1e-01) |
| wnSPIND_spindle_W_FRQ_all | -0% (3.4e-01) | -1% (7.3e-04*^) | 1% (8.4e-02) |
| wnSPIND_spindle_W_FWHM_all | 2% (3.5e-01) | 1% (4.1e-01) | 1% (7.6e-01) |
| wnSPIND_spindle_W_N_all | -1% (8.5e-01) | -9% (1.0e-01) | 8% (2.7e-01) |
| wnSPIND_spindle_W_Q_all | -2% (2.0e-01) | -0% (9.4e-01) | -2% (2.4e-01) |

The Benjamini-Hochberg correction was applied across all tests in the table simultaneously, as the pairwise comparisons involved overlapping subsets of participants. *Original *p*<0.05; ^*p*<0.05 after Benjamini-Hochberg correction for false discovery rate. BMI, body mass index; IH, idiopathic hypersomnia; NT2, narcolepsy type 2; Prop, proportion; REM, rapid eye movement; ssi, stage shift index; tst, total sleep time (min); "XtoY", transition from stage X to stage Y. The sleep feature names are abbreviated as follows: the prefix represents the feature set that the feature originates from: wnSM is whole-night hypnogram features; qnSM is quarter-night hypnogram features; qnTP is quarter-night stage transition probability features; qnHYPN is quarter-night hypnodensity features; qnQEEG is quarter-night quantitative electroencephalogram features; wnSPIND is whole-night spindle features. The suffix represents the time resolution of the feature: all is whole night; Q1/2/3/4 is quarter 1/2/3/4 of the night.

**Supplementary Table S4.** Difference in means, expressed as a percentage relative to the second cluster in each pair, along with the original (uncorrected) *p-*values from a *t*-test on the difference, for demographics and sleep features.

| **Feature name** | **Difference in means, expressed as a percentage relative to the second cluster in each pair (*t*-test *p-*value)** | | |
| --- | --- | --- | --- |
|  | **Clusters NT1-C1 vs NT1-C2** | **Clusters NT2-C1 vs NT2-C2** | **Clusters IH-C1 vs  IH-C2** |
| Age (not used in clustering) | 13% (9.2e-02) | 26% (2.3e-03*^) | 1% (9.0e-01) |
| BMI (not used in clustering) | -4% (3.5e-01) | 10% (3.6e-02*) | -2% (5.8e-01) |
| Sex (prop. male) (not used in clustering) | 31% (7.2e-02) | 25% (2.4e-01) | -30% (3.9e-01) |
| Orexin-A (pg/mL) (not used in clustering) | 1% (9.5e-01) | -5% (5.2e-01) | 27% (3.9e-02*) |
| wnSM_REMonSet | 13% (6.8e-01) | -9% (5.7e-01) | -0% (9.7e-01) |
| wnSM_propN1_all | 60% (1.2e-04*^) | 56% (1.1e-03*^) | 8% (5.4e-01) |
| wnSM_propN2_all | -19% (2.8e-05*^) | -4% (2.8e-01) | -1% (7.6e-01) |
| wnSM_propN3_all | -32% (1.5e-06*^) | -21% (8.3e-04*^) | -3% (6.8e-01) |
| wnSM_propR_all | -1% (9.2e-01) | -4% (5.1e-01) | -2% (7.5e-01) |
| wnSM_propW_all | 100% (6.9e-09*^) | 116% (5.9e-07*^) | 19% (2.9e-01) |
| wnSM_ssi_all | 33% (2.3e-06*^) | 43% (2.2e-06*^) | 4% (5.0e-01) |
| wnSM_tst_all | -13% (1.2e-06*^) | -9% (7.9e-04*^) | -4% (2.4e-01) |
| qnSM_propN1_Q1 | 114% (1.0e-05*^) | 49% (2.1e-02*^) | -10% (5.8e-01) |
| qnSM_propN1_Q2 | 80% (2.9e-04*^) | 116% (1.6e-04*^) | 19% (3.6e-01) |
| qnSM_propN1_Q3 | 48% (4.8e-03*^) | 72% (3.3e-03*^) | 34% (9.8e-02) |
| qnSM_propN1_Q4 | 32% (4.7e-02*) | 23% (2.2e-01) | 2% (9.2e-01) |
| qnSM_propN2_Q1 | -13% (4.6e-02*) | 11% (9.8e-02) | 1% (8.7e-01) |
| qnSM_propN2_Q2 | -21% (7.8e-04*^) | -8% (1.7e-01) | -5% (3.7e-01) |
| qnSM_propN2_Q3 | -17% (4.5e-03*^) | -7% (2.3e-01) | 0% (9.5e-01) |
| qnSM_propN2_Q4 | -24% (1.1e-03*^) | -9% (8.5e-02) | -0% (9.3e-01) |
| qnSM_propN3_Q1 | -37% (1.3e-05*^) | -30% (3.1e-05*^) | -2% (7.8e-01) |
| qnSM_propN3_Q2 | -38% (2.1e-03*^) | -19% (1.7e-01) | -1% (9.2e-01) |
| qnSM_propN3_Q3 | -22% (1.9e-01) | -18% (3.2e-01) | -23% (1.9e-01) |
| qnSM_propN3_Q4 | -19% (2.2e-01) | 23% (4.5e-01) | 26% (3.2e-01) |
| qnSM_propR_Q1 | 14% (3.2e-01) | 4% (8.1e-01) | -2% (9.2e-01) |
| qnSM_propR_Q2 | -2% (8.2e-01) | -1% (9.1e-01) | 5% (6.4e-01) |
| qnSM_propR_Q3 | -6% (5.2e-01) | -15% (1.0e-01) | -7% (4.6e-01) |
| qnSM_propR_Q4 | -2% (8.1e-01) | 0% (9.6e-01) | -2% (8.5e-01) |
| qnSM_propW_Q1 | 159% (2.7e-06*^) | 179% (3.2e-04*^) | 24% (4.7e-01) |
| qnSM_propW_Q2 | 105% (4.6e-06*^) | 142% (5.4e-05*^) | 29% (2.5e-01) |
| qnSM_propW_Q3 | 65% (5.2e-04*^) | 175% (1.1e-04*^) | 53% (1.6e-01) |
| qnSM_propW_Q4 | 96% (2.0e-06*^) | 30% (1.8e-01) | -9% (7.2e-01) |
| qnSM_ssi_Q1 | 32% (6.6e-04*^) | 51% (2.0e-05*^) | -2% (8.7e-01) |
| qnSM_ssi_Q2 | 37% (1.9e-04*^) | 42% (8.2e-05*^) | 11% (2.5e-01) |
| qnSM_ssi_Q3 | 28% (4.3e-04*^) | 47% (8.2e-05*^) | 13% (1.9e-01) |
| qnSM_ssi_Q4 | 36% (4.5e-05*^) | 35% (1.5e-03*^) | -2% (8.6e-01) |
| qnSM_tst_Q1 | -14% (8.3e-06*^) | -9% (1.3e-03*^) | -4% (3.0e-01) |
| qnSM_tst_Q2 | -15% (2.6e-05*^) | -9% (5.5e-04*^) | -4% (2.6e-01) |
| qnSM_tst_Q3 | -11% (7.1e-04*^) | -11% (5.5e-04*^) | -5% (1.4e-01) |
| qnSM_tst_Q4 | -11% (9.7e-05*^) | -5% (7.0e-02) | -1% (7.7e-01) |
| qnTM_N1toN1_Q1 | 27% (1.6e-02*^) | -28% (4.9e-02*) | -6% (6.7e-01) |
| qnTM_N1toN1_Q2 | 14% (1.7e-01) | 46% (7.3e-02) | 13% (5.3e-01) |
| qnTM_N1toN1_Q3 | 14% (9.6e-02) | -3% (8.6e-01) | 27% (2.1e-01) |
| qnTM_N1toN1_Q4 | -3% (7.1e-01) | -12% (4.8e-01) | 6% (7.0e-01) |
| qnTM_N1toN2_Q1 | -26% (6.2e-03*^) | 19% (1.3e-01) | 3% (7.7e-01) |
| qnTM_N1toN2_Q2 | -17% (1.0e-01) | -10% (5.0e-01) | -7% (6.3e-01) |
| qnTM_N1toN2_Q3 | -23% (3.0e-02*^) | 8% (5.7e-01) | -15% (2.2e-01) |
| qnTM_N1toN2_Q4 | -21% (7.6e-02) | 16% (2.5e-01) | 5% (6.4e-01) |
| qnTM_N1toN3_Q1 | n/a | -100% (4.4e-01) | n/a |
| qnTM_N1toN3_Q2 | n/a | n/a | -100% (3.7e-01) |
| qnTM_N1toN3_Q3 | n/a | n/a | n/a |
| qnTM_N1toN3_Q4 | n/a | -100% (4.4e-01) | n/a |
| qnTM_N1toR_Q1 | -8% (7.9e-01) | -20% (6.4e-01) | 11% (8.7e-01) |
| qnTM_N1toR_Q2 | -29% (1.8e-01) | 40% (5.4e-01) | 72% (1.6e-01) |
| qnTM_N1toR_Q3 | -15% (4.4e-01) | -39% (2.6e-01) | 9% (8.2e-01) |
| qnTM_N1toR_Q4 | 37% (8.9e-02) | -37% (1.4e-01) | -59% (7.4e-02) |
| qnTM_N1toW_Q1 | 87% (5.5e-03*^) | 254% (7.0e-04*^) | 7% (8.6e-01) |
| qnTM_N1toW_Q2 | 90% (4.6e-04*^) | 65% (2.2e-01) | 137% (1.5e-02*^) |
| qnTM_N1toW_Q3 | 45% (5.7e-03*^) | 102% (2.8e-02*) | 35% (4.0e-01) |
| qnTM_N1toW_Q4 | 70% (5.0e-04*^) | 80% (3.2e-02*) | 30% (3.5e-01) |
| qnTM_N2toN1_Q1 | 267% (4.6e-03*^) | 48% (1.6e-01) | -31% (2.8e-01) |
| qnTM_N2toN1_Q2 | 189% (1.2e-03*^) | 61% (1.5e-01) | -30% (4.7e-01) |
| qnTM_N2toN1_Q3 | 168% (7.7e-04*^) | 131% (5.0e-03*^) | 136% (4.6e-02*) |
| qnTM_N2toN1_Q4 | 196% (3.5e-05*^) | 45% (2.6e-01) | 7% (8.3e-01) |
| qnTM_N2toN2_Q1 | -5% (2.8e-02*^) | -2% (1.2e-01) | 0% (8.5e-01) |
| qnTM_N2toN2_Q2 | -5% (1.1e-04*^) | -3% (3.7e-03*^) | -0% (7.4e-01) |
| qnTM_N2toN2_Q3 | -5% (6.7e-05*^) | -4% (1.1e-03*^) | -3% (5.0e-02) |
| qnTM_N2toN2_Q4 | -7% (5.5e-06*^) | -3% (7.6e-04*^) | -0% (8.9e-01) |
| qnTM_N2toN3_Q1 | -17% (3.6e-01) | -23% (2.1e-01) | -1% (9.2e-01) |
| qnTM_N2toN3_Q2 | -27% (1.7e-01) | -27% (2.9e-01) | -6% (8.1e-01) |
| qnTM_N2toN3_Q3 | 33% (2.7e-01) | -8% (7.4e-01) | -6% (7.8e-01) |
| qnTM_N2toN3_Q4 | 51% (1.3e-01) | 24% (4.9e-01) | 17% (5.3e-01) |
| qnTM_N2toR_Q1 | -34% (1.0e-01) | -15% (5.1e-01) | 11% (5.6e-01) |
| qnTM_N2toR_Q2 | -24% (1.9e-01) | 45% (1.1e-01) | 4% (8.1e-01) |
| qnTM_N2toR_Q3 | -37% (7.2e-02) | 16% (5.1e-01) | 17% (4.9e-01) |
| qnTM_N2toR_Q4 | 42% (4.1e-01) | 13% (4.4e-01) | -2% (9.2e-01) |
| qnTM_N2toW_Q1 | 62% (2.5e-02*^) | 129% (1.4e-04*^) | 5% (8.3e-01) |
| qnTM_N2toW_Q2 | 66% (1.5e-04*^) | 100% (9.8e-07*^) | 18% (3.2e-01) |
| qnTM_N2toW_Q3 | 46% (2.3e-03*^) | 115% (1.6e-03*^) | 80% (3.8e-02*) |
| qnTM_N2toW_Q4 | 41% (1.7e-02*^) | 73% (3.6e-05*^) | -1% (9.3e-01) |
| qnTM_N3toN1_Q1 | 841% (2.7e-01) | 437% (2.0e-01) | -90% (4.5e-01) |
| qnTM_N3toN1_Q2 | 67% (7.3e-01) | -100% (2.4e-01) | 42% (7.6e-01) |
| qnTM_N3toN1_Q3 | 190% (5.3e-01) | 9% (9.6e-01) | n/a |
| qnTM_N3toN1_Q4 | n/a | -63% (5.2e-01) | n/a |
| qnTM_N3toN2_Q1 | 76% (2.3e-01) | 38% (2.7e-01) | -11% (6.4e-01) |
| qnTM_N3toN2_Q2 | -36% (1.7e-01) | 53% (2.3e-01) | -0% (9.9e-01) |
| qnTM_N3toN2_Q3 | 140% (1.5e-01) | 67% (2.1e-01) | -12% (7.7e-01) |
| qnTM_N3toN2_Q4 | 42% (5.0e-01) | 50% (4.1e-01) | -42% (4.4e-01) |
| qnTM_N3toN3_Q1 | -10% (7.0e-03*^) | -5% (4.0e-02*) | 1% (6.1e-01) |
| qnTM_N3toN3_Q2 | -36% (8.5e-07*^) | -7% (3.2e-01) | 4% (3.2e-01) |
| qnTM_N3toN3_Q3 | -8% (4.4e-01) | -18% (8.8e-02) | -6% (5.7e-01) |
| qnTM_N3toN3_Q4 | -5% (6.4e-01) | -13% (4.8e-01) | 8% (6.7e-01) |
| qnTM_N3toR_Q1 | n/a | -39% (6.2e-01) | 1430% (5.5e-02) |
| qnTM_N3toR_Q2 | -17% (8.9e-01) | -79% (2.1e-01) | -57% (6.0e-01) |
| qnTM_N3toR_Q3 | -100% (3.4e-01) | n/a | -100% (5.0e-01) |
| qnTM_N3toR_Q4 | -100% (3.4e-01) | 71% (6.9e-01) | 1044% (1.8e-01) |
| qnTM_N3toW_Q1 | -5% (8.3e-01) | 107% (4.5e-02*) | 56% (1.8e-02*) |
| qnTM_N3toW_Q2 | -69% (1.7e-03*^) | -13% (7.0e-01) | 43% (1.2e-01) |
| qnTM_N3toW_Q3 | -12% (8.2e-01) | 30% (5.3e-01) | -16% (6.4e-01) |
| qnTM_N3toW_Q4 | -52% (3.1e-01) | 171% (2.8e-01) | -22% (6.4e-01) |
| qnTM_RtoN1_Q1 | 19% (5.8e-01) | 16% (7.8e-01) | 61% (4.4e-01) |
| qnTM_RtoN1_Q2 | -10% (6.9e-01) | -50% (1.9e-01) | 25% (6.5e-01) |
| qnTM_RtoN1_Q3 | 6% (9.0e-01) | -50% (1.4e-01) | -7% (8.4e-01) |
| qnTM_RtoN1_Q4 | 57% (1.4e-01) | -10% (7.6e-01) | -6% (8.6e-01) |
| qnTM_RtoN2_Q1 | -49% (3.8e-02*) | -56% (4.7e-02*) | -3% (9.3e-01) |
| qnTM_RtoN2_Q2 | 48% (6.1e-01) | -27% (2.2e-01) | 41% (3.7e-01) |
| qnTM_RtoN2_Q3 | 15% (6.8e-01) | 74% (4.2e-01) | -52% (7.0e-02) |
| qnTM_RtoN2_Q4 | -8% (7.7e-01) | 48% (2.6e-01) | 29% (4.2e-01) |
| qnTM_RtoN3_Q1 | n/a | n/a | -100% (5.0e-01) |
| qnTM_RtoN3_Q2 | n/a | n/a | n/a |
| qnTM_RtoN3_Q3 | -100% (3.4e-01) | n/a | n/a |
| qnTM_RtoN3_Q4 | n/a | n/a | -100% (5.0e-01) |
| qnTM_RtoR_Q1 | 1% (8.7e-01) | 9% (3.1e-01) | -6% (5.4e-01) |
| qnTM_RtoR_Q2 | -13% (6.8e-03*^) | -2% (1.5e-01) | -0% (1.0e+00) |
| qnTM_RtoR_Q3 | -3% (4.1e-01) | -4% (1.5e-01) | -2% (3.5e-01) |
| qnTM_RtoR_Q4 | -5% (1.3e-03*^) | -5% (6.5e-02) | 1% (3.9e-01) |
| qnTM_RtoW_Q1 | 104% (3.3e-03*^) | 108% (1.2e-02*^) | -17% (6.7e-01) |
| qnTM_RtoW_Q2 | 61% (1.1e-02*^) | 127% (7.1e-04*^) | 11% (7.1e-01) |
| qnTM_RtoW_Q3 | 15% (5.2e-01) | 104% (4.5e-03*^) | -2% (9.2e-01) |
| qnTM_RtoW_Q4 | 69% (2.9e-03*^) | 26% (2.2e-01) | -26% (1.2e-01) |
| qnTM_WtoN1_Q1 | -30% (2.7e-02*^) | 38% (1.0e-01) | 6% (7.7e-01) |
| qnTM_WtoN1_Q2 | -12% (3.9e-01) | 8% (6.8e-01) | 2% (8.9e-01) |
| qnTM_WtoN1_Q3 | -28% (5.1e-03*^) | -19% (2.2e-01) | -1% (9.4e-01) |
| qnTM_WtoN1_Q4 | -28% (3.3e-03*^) | -7% (6.1e-01) | -8% (5.5e-01) |
| qnTM_WtoN2_Q1 | -58% (4.9e-04*^) | -39% (4.4e-02*) | -10% (5.9e-01) |
| qnTM_WtoN2_Q2 | -64% (1.4e-04*^) | -43% (2.7e-03*^) | -13% (4.7e-01) |
| qnTM_WtoN2_Q3 | -50% (5.9e-03*^) | -4% (8.7e-01) | -9% (6.5e-01) |
| qnTM_WtoN2_Q4 | -55% (1.1e-02*^) | -20% (3.3e-01) | 14% (6.1e-01) |
| qnTM_WtoN3_Q1 | -94% (4.0e-02*) | -90% (3.1e-01) | 76% (5.2e-01) |
| qnTM_WtoN3_Q2 | -100% (1.5e-01) | -3% (9.8e-01) | -74% (4.6e-01) |
| qnTM_WtoN3_Q3 | -100% (3.4e-01) | -100% (4.4e-01) | n/a |
| qnTM_WtoN3_Q4 | n/a | -100% (4.4e-01) | n/a |
| qnTM_WtoR_Q1 | 51% (3.4e-01) | -55% (1.8e-01) | 84% (2.8e-01) |
| qnTM_WtoR_Q2 | -26% (3.7e-01) | -36% (3.2e-01) | 69% (2.6e-01) |
| qnTM_WtoR_Q3 | -25% (3.3e-01) | -51% (5.8e-02) | -3% (9.1e-01) |
| qnTM_WtoR_Q4 | -8% (6.8e-01) | 4% (8.9e-01) | -31% (2.3e-01) |
| qnTM_WtoW_Q1 | 60% (3.0e-06*^) | 72% (1.1e-03*^) | 5% (7.9e-01) |
| qnTM_WtoW_Q2 | 33% (5.8e-04*^) | 64% (3.5e-04*^) | 16% (3.7e-01) |
| qnTM_WtoW_Q3 | 32% (3.1e-05*^) | 45% (2.0e-02*^) | 10% (6.0e-01) |
| qnTM_WtoW_Q4 | 31% (9.8e-05*^) | 15% (3.1e-01) | 11% (4.6e-01) |
| qnHYPN_N1N2_Q1 | 56% (3.1e-05*^) | 107% (4.8e-10*^) | 48% (1.6e-04*^) |
| qnHYPN_N1N2_Q2 | 39% (2.2e-04*^) | 108% (2.5e-08*^) | 97% (4.2e-11*^) |
| qnHYPN_N1N2_Q3 | 32% (9.6e-04*^) | 95% (6.3e-07*^) | 103% (6.1e-14*^) |
| qnHYPN_N1N2_Q4 | -1% (9.0e-01) | 94% (4.3e-08*^) | 76% (1.2e-11*^) |
| qnHYPN_N1N3_Q1 | -52% (8.7e-03*^) | 126% (2.9e-03*^) | 348% (2.4e-17*^) |
| qnHYPN_N1N3_Q2 | -55% (2.9e-02*^) | 22% (4.5e-01) | 395% (1.2e-15*^) |
| qnHYPN_N1N3_Q3 | -61% (5.9e-03*^) | 67% (1.2e-01) | 343% (1.8e-07*^) |
| qnHYPN_N1N3_Q4 | -61% (7.0e-03*^) | 72% (7.8e-02) | 168% (1.2e-06*^) |
| qnHYPN_N1R_Q1 | -26% (3.8e-02*) | 12% (5.1e-01) | -16% (3.4e-01) |
| qnHYPN_N1R_Q2 | -32% (7.2e-03*^) | 48% (5.2e-03*^) | 22% (1.6e-01) |
| qnHYPN_N1R_Q3 | -34% (7.5e-04*^) | 28% (8.0e-02) | 9% (5.2e-01) |
| qnHYPN_N1R_Q4 | -29% (2.0e-03*^) | 35% (2.5e-02*^) | 21% (9.7e-02) |
| qnHYPN_N1_Q1 | 53% (1.9e-05*^) | 116% (2.0e-11*^) | 53% (1.3e-04*^) |
| qnHYPN_N1_Q2 | 50% (2.0e-05*^) | 155% (6.6e-13*^) | 117% (9.0e-15*^) |
| qnHYPN_N1_Q3 | 43% (1.7e-05*^) | 129% (4.5e-11*^) | 111% (1.1e-15*^) |
| qnHYPN_N1_Q4 | 25% (1.2e-02*^) | 108% (2.0e-09*^) | 81% (2.4e-11*^) |
| qnHYPN_N2N3_Q1 | -28% (1.2e-02*^) | -31% (4.2e-04*^) | -21% (3.6e-02*) |
| qnHYPN_N2N3_Q2 | -62% (1.1e-07*^) | -39% (1.4e-03*^) | -43% (2.2e-05*^) |
| qnHYPN_N2N3_Q3 | -49% (7.7e-04*^) | -44% (9.7e-04*^) | -45% (1.3e-03*^) |
| qnHYPN_N2N3_Q4 | -34% (2.3e-02*^) | -39% (5.1e-02) | -19% (3.2e-01) |
| qnHYPN_N2R_Q1 | -55% (6.7e-05*^) | -41% (2.5e-02*^) | -48% (7.9e-04*^) |
| qnHYPN_N2R_Q2 | -62% (7.4e-06*^) | -47% (9.1e-05*^) | -37% (1.5e-02*^) |
| qnHYPN_N2R_Q3 | -66% (4.7e-11*^) | -51% (4.1e-04*^) | -46% (1.3e-03*^) |
| qnHYPN_N2R_Q4 | -68% (2.5e-13*^) | -38% (1.4e-02*^) | -41% (9.3e-03*^) |
| qnHYPN_N2_Q1 | 10% (2.0e-01) | 3% (6.8e-01) | -10% (1.2e-01) |
| qnHYPN_N2_Q2 | -17% (1.1e-02*^) | -16% (8.2e-04*^) | -19% (4.8e-05*^) |
| qnHYPN_N2_Q3 | -17% (5.4e-03*^) | -24% (1.9e-05*^) | -14% (6.5e-03*^) |
| qnHYPN_N2_Q4 | -30% (3.5e-06*^) | -22% (4.3e-05*^) | -8% (1.3e-01) |
| qnHYPN_N3R_Q1 | -84% (1.4e-02*^) | 64% (6.0e-01) | -16% (5.2e-01) |
| qnHYPN_N3R_Q2 | -68% (2.2e-03*^) | -81% (1.1e-01) | -10% (7.3e-01) |
| qnHYPN_N3R_Q3 | -87% (5.7e-04*^) | -13% (7.8e-01) | -8% (8.6e-01) |
| qnHYPN_N3R_Q4 | -82% (1.2e-04*^) | -57% (6.3e-02) | -45% (2.9e-01) |
| qnHYPN_N3_Q1 | -65% (7.8e-12*^) | -56% (1.1e-09*^) | -36% (6.9e-04*^) |
| qnHYPN_N3_Q2 | -77% (2.0e-10*^) | -53% (7.2e-05*^) | -44% (1.3e-03*^) |
| qnHYPN_N3_Q3 | -72% (1.3e-07*^) | -41% (4.1e-02*) | -53% (5.1e-03*^) |
| qnHYPN_N3_Q4 | -65% (6.5e-05*^) | -14% (6.5e-01) | -19% (4.7e-01) |
| qnHYPN_R_Q1 | -21% (1.3e-01) | -26% (1.5e-01) | -54% (6.4e-04*^) |
| qnHYPN_R_Q2 | -25% (5.0e-02) | -30% (1.5e-02*^) | -45% (3.3e-05*^) |
| qnHYPN_R_Q3 | -32% (3.7e-03*^) | -37% (8.9e-05*^) | -56% (2.3e-09*^) |
| qnHYPN_R_Q4 | -19% (9.0e-02) | -30% (1.9e-03*^) | -54% (8.3e-08*^) |
| qnHYPN_WN1_Q1 | 113% (1.9e-06*^) | 240% (2.7e-12*^) | 148% (5.7e-10*^) |
| qnHYPN_WN1_Q2 | 122% (8.1e-09*^) | 324% (1.5e-13*^) | 297% (5.7e-17*^) |
| qnHYPN_WN1_Q3 | 113% (1.6e-09*^) | 309% (2.1e-11*^) | 286% (1.9e-16*^) |
| qnHYPN_WN1_Q4 | 104% (1.7e-08*^) | 202% (1.5e-09*^) | 167% (2.3e-12*^) |
| qnHYPN_WN2_Q1 | 15% (4.6e-01) | 291% (1.5e-09*^) | 378% (1.7e-18*^) |
| qnHYPN_WN2_Q2 | 15% (3.9e-01) | 205% (1.4e-06*^) | 497% (2.2e-21*^) |
| qnHYPN_WN2_Q3 | 3% (8.5e-01) | 226% (2.5e-07*^) | 435% (3.3e-18*^) |
| qnHYPN_WN2_Q4 | 1% (9.3e-01) | 186% (5.5e-07*^) | 276% (5.8e-16*^) |
| qnHYPN_WN3_Q1 | -84% (1.2e-03*^) | 240% (4.5e-03*^) | 2790% (8.2e-13*^) |
| qnHYPN_WN3_Q2 | -83% (2.5e-03*^) | 222% (2.7e-03*^) | 2017% (7.2e-12*^) |
| qnHYPN_WN3_Q3 | -86% (1.6e-03*^) | 335% (2.7e-03*^) | 930% (2.2e-07*^) |
| qnHYPN_WN3_Q4 | -88% (1.7e-03*^) | 426% (9.0e-03*^) | 353% (5.6e-03*^) |
| qnHYPN_WR_Q1 | 35% (1.3e-01) | 88% (1.6e-02*^) | 34% (2.8e-01) |
| qnHYPN_WR_Q2 | 28% (1.7e-01) | 147% (7.8e-08*^) | 156% (2.1e-08*^) |
| qnHYPN_WR_Q3 | 20% (1.9e-01) | 163% (5.0e-05*^) | 94% (3.7e-05*^) |
| qnHYPN_WR_Q4 | 41% (6.5e-03*^) | 127% (2.4e-05*^) | 107% (2.5e-06*^) |
| qnHYPN_W_Q1 | 107% (4.4e-06*^) | 247% (2.8e-10*^) | 266% (6.8e-13*^) |
| qnHYPN_W_Q2 | 121% (1.0e-08*^) | 311% (4.1e-13*^) | 348% (2.9e-19*^) |
| qnHYPN_W_Q3 | 105% (2.1e-07*^) | 306% (1.8e-11*^) | 256% (1.3e-11*^) |
| qnHYPN_W_Q4 | 134% (3.9e-10*^) | 147% (2.2e-07*^) | 104% (3.0e-05*^) |
| qnHYPN_entropy_Q1 | -0% (9.7e-01) | 45% (5.4e-09*^) | 71% (1.7e-20*^) |
| qnHYPN_entropy_Q2 | -4% (4.8e-01) | 46% (5.1e-08*^) | 85% (3.1e-24*^) |
| qnHYPN_entropy_Q3 | -0% (9.4e-01) | 52% (1.6e-08*^) | 75% (1.1e-18*^) |
| qnHYPN_entropy_Q4 | -2% (6.8e-01) | 56% (2.4e-10*^) | 64% (3.4e-16*^) |
| qnQEEG_mean_alpha_N1_Q1 | 39% (2.4e-04*^) | 20% (7.8e-02) | -33% (6.8e-05*^) |
| qnQEEG_mean_alpha_N1_Q2 | 45% (1.9e-06*^) | 66% (2.3e-07*^) | -22% (1.1e-02*^) |
| qnQEEG_mean_alpha_N1_Q3 | 52% (3.4e-08*^) | 57% (4.7e-06*^) | -21% (6.5e-03*^) |
| qnQEEG_mean_alpha_N1_Q4 | 48% (1.1e-06*^) | 38% (4.2e-04*^) | -20% (2.3e-02*) |
| qnQEEG_mean_alpha_N2_Q1 | 59% (2.7e-10*^) | 35% (2.9e-06*^) | -23% (2.1e-04*^) |
| qnQEEG_mean_alpha_N2_Q2 | 52% (1.1e-09*^) | 52% (2.9e-09*^) | -22% (4.5e-04*^) |
| qnQEEG_mean_alpha_N2_Q3 | 54% (3.5e-09*^) | 44% (5.2e-07*^) | -16% (5.0e-03*^) |
| qnQEEG_mean_alpha_N2_Q4 | 58% (1.0e-09*^) | 46% (1.9e-08*^) | -17% (9.7e-03*^) |
| qnQEEG_mean_alpha_N3_Q1 | 69% (9.0e-10*^) | 39% (8.1e-04*^) | -22% (3.1e-02*) |
| qnQEEG_mean_alpha_N3_Q2 | 51% (1.8e-04*^) | 39% (2.6e-03*^) | -28% (2.4e-03*^) |
| qnQEEG_mean_alpha_N3_Q3 | 107% (2.5e-08*^) | 34% (4.1e-03*^) | -23% (4.6e-02*) |
| qnQEEG_mean_alpha_N3_Q4 | 61% (1.5e-04*^) | 42% (1.0e-02*^) | -22% (6.0e-02) |
| qnQEEG_mean_alpha_REM_Q1 | 35% (3.0e-04*^) | 28% (2.1e-03*^) | -20% (9.2e-03*^) |
| qnQEEG_mean_alpha_REM_Q2 | 43% (1.1e-05*^) | 33% (1.4e-04*^) | -25% (1.9e-04*^) |
| qnQEEG_mean_alpha_REM_Q3 | 45% (2.2e-06*^) | 22% (4.1e-03*^) | -26% (1.5e-04*^) |
| qnQEEG_mean_alpha_REM_Q4 | 41% (8.1e-06*^) | 23% (1.4e-03*^) | -26% (1.3e-04*^) |
| qnQEEG_mean_alpha_W_Q1 | 66% (2.5e-07*^) | 78% (5.7e-08*^) | -26% (1.7e-02*) |
| qnQEEG_mean_alpha_W_Q2 | 55% (6.6e-07*^) | 78% (5.0e-09*^) | -21% (2.4e-02*) |
| qnQEEG_mean_alpha_W_Q3 | 61% (9.0e-08*^) | 53% (3.9e-06*^) | -21% (2.4e-02*) |
| qnQEEG_mean_alpha_W_Q4 | 54% (2.2e-06*^) | 34% (1.9e-03*^) | -23% (1.5e-02*^) |
| qnQEEG_mean_beta_N1_Q1 | 81% (2.3e-10*^) | 10% (3.6e-01) | -23% (1.4e-02*^) |
| qnQEEG_mean_beta_N1_Q2 | 86% (2.3e-11*^) | 51% (2.3e-03*^) | -30% (3.7e-03*^) |
| qnQEEG_mean_beta_N1_Q3 | 74% (1.1e-09*^) | 26% (2.7e-02*) | -28% (5.0e-03*^) |
| qnQEEG_mean_beta_N1_Q4 | 68% (8.7e-10*^) | 16% (1.1e-01) | -25% (7.2e-02) |
| qnQEEG_mean_beta_N2_Q1 | 106% (5.4e-14*^) | 35% (1.1e-03*^) | -19% (1.5e-02*^) |
| qnQEEG_mean_beta_N2_Q2 | 117% (2.7e-07*^) | 65% (1.2e-08*^) | -21% (2.1e-02*) |
| qnQEEG_mean_beta_N2_Q3 | 97% (5.0e-08*^) | 60% (8.6e-07*^) | -7% (4.2e-01) |
| qnQEEG_mean_beta_N2_Q4 | 102% (5.6e-07*^) | 44% (1.1e-05*^) | -19% (2.3e-01) |
| qnQEEG_mean_beta_N3_Q1 | 117% (4.1e-06*^) | 97% (5.6e-03*^) | -3% (7.6e-01) |
| qnQEEG_mean_beta_N3_Q2 | 173% (3.0e-02*^) | 44% (4.1e-04*^) | -19% (3.8e-02*) |
| qnQEEG_mean_beta_N3_Q3 | 126% (2.3e-07*^) | 39% (7.3e-03*^) | 5% (8.7e-01) |
| qnQEEG_mean_beta_N3_Q4 | 187% (6.4e-02) | 48% (9.9e-03*^) | -15% (3.3e-01) |
| qnQEEG_mean_beta_REM_Q1 | 97% (1.9e-13*^) | 36% (3.1e-03*^) | -12% (2.8e-01) |
| qnQEEG_mean_beta_REM_Q2 | 117% (2.9e-18*^) | 56% (1.4e-06*^) | -8% (3.3e-01) |
| qnQEEG_mean_beta_REM_Q3 | 109% (3.0e-17*^) | 40% (2.0e-04*^) | -15% (6.7e-02) |
| qnQEEG_mean_beta_REM_Q4 | 95% (1.1e-16*^) | 40% (3.1e-05*^) | -8% (3.4e-01) |
| qnQEEG_mean_beta_W_Q1 | 121% (1.3e-13*^) | 62% (2.0e-03*^) | -34% (1.9e-02*) |
| qnQEEG_mean_beta_W_Q2 | 111% (3.4e-12*^) | 82% (1.5e-06*^) | -27% (4.7e-02*) |
| qnQEEG_mean_beta_W_Q3 | 79% (2.8e-08*^) | 53% (4.0e-04*^) | -26% (2.3e-01) |
| qnQEEG_mean_beta_W_Q4 | 84% (1.2e-08*^) | 22% (9.5e-02) | -24% (2.2e-01) |
| qnQEEG_mean_delta_N1_Q1 | -25% (3.4e-10*^) | -7% (1.6e-01) | 28% (2.1e-08*^) |
| qnQEEG_mean_delta_N1_Q2 | -27% (2.4e-12*^) | -19% (9.6e-07*^) | 19% (4.3e-06*^) |
| qnQEEG_mean_delta_N1_Q3 | -28% (2.6e-15*^) | -15% (1.1e-04*^) | 17% (1.3e-05*^) |
| qnQEEG_mean_delta_N1_Q4 | -25% (2.0e-12*^) | -12% (8.1e-04*^) | 16% (1.5e-04*^) |
| qnQEEG_mean_delta_N2_Q1 | -19% (3.4e-14*^) | -7% (1.6e-04*^) | 10% (3.5e-06*^) |
| qnQEEG_mean_delta_N2_Q2 | -20% (8.6e-14*^) | -13% (1.1e-09*^) | 9% (1.2e-05*^) |
| qnQEEG_mean_delta_N2_Q3 | -19% (5.4e-13*^) | -11% (6.7e-06*^) | 6% (2.4e-03*^) |
| qnQEEG_mean_delta_N2_Q4 | -21% (3.6e-14*^) | -11% (6.1e-07*^) | 8% (3.3e-03*^) |
| qnQEEG_mean_delta_N3_Q1 | -9% (3.0e-10*^) | -6% (2.0e-04*^) | 3% (5.6e-02) |
| qnQEEG_mean_delta_N3_Q2 | -12% (2.3e-06*^) | -5% (1.8e-03*^) | 5% (3.9e-04*^) |
| qnQEEG_mean_delta_N3_Q3 | -14% (1.4e-09*^) | -4% (4.4e-02*) | 3% (1.6e-01) |
| qnQEEG_mean_delta_N3_Q4 | -10% (3.4e-04*^) | -7% (2.3e-02*^) | 6% (3.9e-02*) |
| qnQEEG_mean_delta_REM_Q1 | -16% (1.1e-06*^) | -9% (4.1e-03*^) | 15% (2.3e-05*^) |
| qnQEEG_mean_delta_REM_Q2 | -21% (1.2e-09*^) | -12% (5.1e-05*^) | 17% (4.9e-08*^) |
| qnQEEG_mean_delta_REM_Q3 | -20% (3.7e-12*^) | -7% (1.1e-02*^) | 19% (1.7e-08*^) |
| qnQEEG_mean_delta_REM_Q4 | -19% (3.3e-11*^) | -8% (7.4e-04*^) | 15% (4.4e-07*^) |
| qnQEEG_mean_delta_W_Q1 | -27% (1.4e-12*^) | -19% (1.9e-06*^) | 12% (4.8e-03*^) |
| qnQEEG_mean_delta_W_Q2 | -29% (5.3e-15*^) | -20% (2.6e-09*^) | 11% (3.5e-03*^) |
| qnQEEG_mean_delta_W_Q3 | -28% (6.8e-14*^) | -16% (9.9e-06*^) | 13% (1.2e-03*^) |
| qnQEEG_mean_delta_W_Q4 | -26% (5.5e-12*^) | -10% (6.9e-03*^) | 17% (5.9e-05*^) |
| qnQEEG_mean_gamma_N1_Q1 | 68% (1.2e-03*^) | 14% (4.5e-01) | -24% (1.4e-01) |
| qnQEEG_mean_gamma_N1_Q2 | 72% (9.2e-05*^) | 48% (1.6e-01) | -36% (4.9e-02*) |
| qnQEEG_mean_gamma_N1_Q3 | 56% (1.2e-03*^) | 30% (2.6e-01) | -38% (3.8e-02*) |
| qnQEEG_mean_gamma_N1_Q4 | 50% (4.2e-03*^) | 16% (3.6e-01) | -31% (4.8e-02*) |
| qnQEEG_mean_gamma_N2_Q1 | 136% (1.2e-05*^) | 48% (1.1e-02*^) | -11% (3.9e-01) |
| qnQEEG_mean_gamma_N2_Q2 | 154% (5.7e-03*^) | 72% (2.3e-03*^) | -13% (3.7e-01) |
| qnQEEG_mean_gamma_N2_Q3 | 132% (4.9e-03*^) | 90% (4.3e-03*^) | 9% (5.8e-01) |
| qnQEEG_mean_gamma_N2_Q4 | 162% (3.3e-03*^) | 36% (4.9e-02*) | -3% (8.1e-01) |
| qnQEEG_mean_gamma_N3_Q1 | 169% (4.5e-03*^) | 147% (4.0e-02*) | -2% (9.1e-01) |
| qnQEEG_mean_gamma_N3_Q2 | 340% (7.5e-02) | 33% (5.7e-02) | -10% (5.0e-01) |
| qnQEEG_mean_gamma_N3_Q3 | 161% (8.7e-04*^) | 34% (1.0e-01) | 30% (6.8e-01) |
| qnQEEG_mean_gamma_N3_Q4 | 460% (1.5e-01) | 38% (1.7e-01) | -13% (5.9e-01) |
| qnQEEG_mean_gamma_REM_Q1 | 100% (5.7e-07*^) | 34% (1.1e-01) | 18% (3.5e-01) |
| qnQEEG_mean_gamma_REM_Q2 | 127% (7.3e-10*^) | 70% (5.8e-03*^) | 25% (1.1e-01) |
| qnQEEG_mean_gamma_REM_Q3 | 129% (2.9e-12*^) | 46% (1.5e-02*^) | 2% (8.9e-01) |
| qnQEEG_mean_gamma_REM_Q4 | 109% (2.9e-10*^) | 36% (3.3e-02*) | 9% (4.7e-01) |
| qnQEEG_mean_gamma_W_Q1 | 130% (7.7e-06*^) | 69% (2.4e-02*^) | -29% (1.2e-01) |
| qnQEEG_mean_gamma_W_Q2 | 138% (3.5e-05*^) | 86% (3.2e-03*^) | -25% (2.1e-01) |
| qnQEEG_mean_gamma_W_Q3 | 100% (1.4e-04*^) | 77% (1.4e-02*^) | -19% (3.5e-01) |
| qnQEEG_mean_gamma_W_Q4 | 126% (1.3e-05*^) | 21% (3.4e-01) | -17% (3.3e-01) |
| qnQEEG_mean_sigma_N1_Q1 | 72% (1.3e-10*^) | 23% (8.5e-03*^) | -20% (7.8e-03*^) |
| qnQEEG_mean_sigma_N1_Q2 | 71% (6.0e-10*^) | 23% (2.4e-02*^) | -31% (3.3e-04*^) |
| qnQEEG_mean_sigma_N1_Q3 | 64% (1.0e-10*^) | 32% (1.6e-03*^) | -10% (2.1e-01) |
| qnQEEG_mean_sigma_N1_Q4 | 65% (1.2e-10*^) | 29% (3.7e-03*^) | -19% (1.6e-02*^) |
| qnQEEG_mean_sigma_N2_Q1 | 55% (1.0e-08*^) | 16% (2.0e-02*^) | -9% (2.3e-01) |
| qnQEEG_mean_sigma_N2_Q2 | 40% (2.1e-05*^) | 21% (7.2e-03*^) | -6% (4.3e-01) |
| qnQEEG_mean_sigma_N2_Q3 | 33% (2.1e-04*^) | 7% (4.0e-01) | 6% (5.0e-01) |
| qnQEEG_mean_sigma_N2_Q4 | 42% (2.3e-05*^) | 7% (3.5e-01) | 6% (4.5e-01) |
| qnQEEG_mean_sigma_N3_Q1 | 91% (9.0e-10*^) | 41% (3.9e-04*^) | 15% (2.2e-01) |
| qnQEEG_mean_sigma_N3_Q2 | 70% (3.9e-05*^) | 48% (7.2e-04*^) | -5% (5.2e-01) |
| qnQEEG_mean_sigma_N3_Q3 | 123% (1.3e-08*^) | 8% (4.8e-01) | 18% (1.6e-01) |
| qnQEEG_mean_sigma_N3_Q4 | 34% (3.3e-02*) | 14% (4.2e-01) | 14% (3.9e-01) |
| qnQEEG_mean_sigma_REM_Q1 | 63% (3.7e-10*^) | 33% (7.5e-04*^) | -22% (6.2e-03*^) |
| qnQEEG_mean_sigma_REM_Q2 | 78% (9.1e-12*^) | 53% (3.5e-07*^) | -24% (3.9e-04*^) |
| qnQEEG_mean_sigma_REM_Q3 | 83% (6.2e-14*^) | 41% (1.3e-05*^) | -23% (7.3e-04*^) |
| qnQEEG_mean_sigma_REM_Q4 | 73% (3.9e-14*^) | 53% (5.9e-09*^) | -20% (4.8e-03*^) |
| qnQEEG_mean_sigma_W_Q1 | 64% (1.5e-10*^) | 56% (2.0e-05*^) | -15% (1.6e-01) |
| qnQEEG_mean_sigma_W_Q2 | 65% (3.0e-10*^) | 51% (1.1e-06*^) | -18% (5.0e-02) |
| qnQEEG_mean_sigma_W_Q3 | 52% (2.6e-08*^) | 28% (8.0e-03*^) | -9% (2.6e-01) |
| qnQEEG_mean_sigma_W_Q4 | 59% (1.4e-09*^) | 16% (4.2e-02*) | -8% (3.6e-01) |
| qnQEEG_mean_theta_N1_Q1 | 1% (8.7e-01) | -9% (8.0e-02) | -13% (1.3e-02*^) |
| qnQEEG_mean_theta_N1_Q2 | 10% (1.2e-01) | 1% (8.9e-01) | -10% (1.1e-01) |
| qnQEEG_mean_theta_N1_Q3 | 9% (1.0e-01) | -5% (4.0e-01) | -13% (1.7e-02*) |
| qnQEEG_mean_theta_N1_Q4 | 3% (4.8e-01) | -2% (7.5e-01) | -4% (4.6e-01) |
| qnQEEG_mean_theta_N2_Q1 | 6% (1.7e-01) | -4% (3.5e-01) | -11% (2.1e-03*^) |
| qnQEEG_mean_theta_N2_Q2 | 11% (3.5e-02*) | 7% (7.9e-02) | -12% (1.2e-03*^) |
| qnQEEG_mean_theta_N2_Q3 | 7% (8.1e-02) | 4% (3.9e-01) | -12% (2.5e-03*^) |
| qnQEEG_mean_theta_N2_Q4 | 5% (2.1e-01) | 3% (4.2e-01) | -11% (2.3e-03*^) |
| qnQEEG_mean_theta_N3_Q1 | 22% (8.7e-05*^) | 12% (5.0e-02) | -12% (2.1e-02*) |
| qnQEEG_mean_theta_N3_Q2 | 26% (6.8e-04*^) | 13% (5.9e-02) | -18% (1.3e-03*^) |
| qnQEEG_mean_theta_N3_Q3 | 37% (4.0e-06*^) | 7% (3.0e-01) | -14% (3.5e-02*) |
| qnQEEG_mean_theta_N3_Q4 | 19% (1.2e-02*^) | 18% (5.8e-02) | -20% (4.3e-03*^) |
| qnQEEG_mean_theta_REM_Q1 | -13% (1.6e-03*^) | -5% (2.0e-01) | -13% (2.5e-03*^) |
| qnQEEG_mean_theta_REM_Q2 | -7% (7.2e-02) | -6% (1.2e-01) | -15% (1.7e-05*^) |
| qnQEEG_mean_theta_REM_Q3 | -7% (3.4e-02*) | -9% (9.6e-03*^) | -16% (1.7e-05*^) |
| qnQEEG_mean_theta_REM_Q4 | -6% (9.6e-02) | -8% (2.5e-02*^) | -12% (1.2e-04*^) |
| qnQEEG_mean_theta_W_Q1 | 8% (2.0e-01) | 6% (3.3e-01) | -10% (1.1e-01) |
| qnQEEG_mean_theta_W_Q2 | 6% (3.3e-01) | 7% (2.2e-01) | -10% (9.4e-02) |
| qnQEEG_mean_theta_W_Q3 | 5% (3.8e-01) | -2% (6.6e-01) | -12% (1.8e-02*) |
| qnQEEG_mean_theta_W_Q4 | 0% (9.6e-01) | -3% (4.9e-01) | -13% (6.3e-03*^) |
| wnSPIND_spindle_W_AMP_all | -12% (1.1e-02*^) | -14% (1.1e-02*^) | -14% (4.8e-03*^) |
| wnSPIND_spindle_W_COUPL_ANGLE_all | 99% (4.8e-02*) | 18% (4.4e-01) | -40% (9.4e-02) |
| wnSPIND_spindle_W_COUPL_MAG_all | 29% (2.4e-02*^) | -20% (2.6e-02*) | -44% (2.1e-07*^) |
| wnSPIND_spindle_W_COUPL_OVERLAP_PCT_all | -7% (7.9e-02) | 3% (5.0e-01) | -6% (1.2e-01) |
| wnSPIND_spindle_W_DENS_all | -12% (6.4e-02) | -18% (2.0e-03*^) | 22% (3.6e-04*^) |
| wnSPIND_spindle_W_DISPERSION_all | -19% (2.0e-03*^) | -7% (1.4e-01) | -1% (8.5e-01) |
| wnSPIND_spindle_W_FRQ_all | 1% (1.5e-01) | -1% (8.8e-02) | 0% (7.5e-01) |
| wnSPIND_spindle_W_FWHM_all | -7% (1.4e-04*^) | -7% (5.2e-03*^) | -2% (3.9e-01) |
| wnSPIND_spindle_W_N_all | -23% (1.9e-03*^) | -29% (3.9e-05*^) | 15% (6.9e-02) |
| wnSPIND_spindle_W_Q_all | -1% (4.6e-01) | -2% (1.7e-01) | -1% (5.4e-01) |

The Benjamini-Hochberg correction was applied separately within each diagnosis group (NT1, NT2, IH), as the comparisons within each group involved non-overlapping subsets of participants. *Original *p*<0.05; ^*p*<0.05 after Benjamini-Hochberg correction for false discovery rate. BMI, body mass index; IH, idiopathic hypersomnia; n/a, not applicable; NT1, narcolepsy type 1; NT2, narcolepsy type 2; Prop, proportion; REM, rapid eye movement; ssi, stage shift index; tst, total sleep time (min); "XtoY", transition from stage X to stage Y. Using the fifth row as an example, the table shows that the mean REM onset in cluster NT1-C1 was 13% higher than in NT1-C2. The sleep feature names are abbreviated as follows: the prefix represents the feature set that the feature originates from: wnSM is whole-night hypnogram features; qnSM is quarter-night hypnogram features; qnTP is quarter-night stage transition probability features; qnHYPN is quarter-night hypnodensity features; qnQEEG is quarter-night qEEG features; wnSPIND is whole-night spindle features. The suffix represents the time resolution of the feature: all is whole night; Q1/2/3/4 is quarter 1/2/3/4 of the night.
